# The asynchronous rise of Northern Hemisphere alpine floras reveals general responses of biotic assembly to orogeny and climate change

**DOI:** 10.1126/sciadv.adz1888

**Published:** 2025-12-19

**Authors:** Wenna Ding, Richard H. Ree, Michael R. May, Philipp Brun, Oskar Hagen, Dirk N. Karger, Alexander Skeels, Loïc Pellissier, Yaowu Xing, Niklaus E. Zimmermann

**Affiliations:** ^1^ State Key Laboratory of Plant Diversity and Specialty Crops, Xishuangbanna Tropical Botanical Garden, Chinese Academy of Sciences, Mengla, Yunnan 666303, China.; ^2^ Swiss Federal Institute for Forest, Snow and Landscape Research WSL, Birmensdorf, 8903 Zürich, Switzerland.; ^3^ Department of Collections, Conservation, and Research, Field Museum, 1400 S DuSable Lake Shore Drive, Chicago, IL 60605, USA.; ^4^ Department of Evolution and Ecology, University of California Davis, 2320 Storer Hall, One Shields Avenue, Davis, CA 95616, USA.; ^5^ Center for Critical Computational Studies (C^3^S), Goethe University Frankfurt, Theodor-W.-Adorno-Platz 1, 60629 Frankfurt am Main, Germany.; ^6^ Senckenberg Biodiversity and Climate Research Centre, Georg-Voigt-Straße 14-16, 60325 Frankfurt am Main, Germany.; ^7^ Research School of Biology, Australian National University, Canberra 0200, Australia.; ^8^ Department of Environmental Systems Science, Ecosystems and Landscape Evolution, Institute of Terrestrial Ecosystems, ETH-Zürich, Zürich 8092, Switzerland.; ^9^ Yunnan Key Laboratory of Forest Ecosystem Stability and Global Change, Xishuangbanna Tropical Botanical Garden, Chinese Academy of Sciences, Mengla, Yunnan 666303, China.

## Abstract

Understanding how biotic assembly processes responded to past geoclimatic changes is key to explaining the origins of mountain biodiversity and the causes of regional disparities in species richness. Here, we jointly reconstructed geographic ranges and biome-niche evolution for 34 diverse plant clades across five major Northern Hemisphere mountain systems and quantified how late Neogene cooling increased arctic-alpine habitat connections across regions. We reveal that, while alpine floras originated asynchronously and were assembled through distinct evolutionary processes over the past 30 million years, general biological responses to orogeny and environmental change are apparent. Across regions, in situ diversification was consistently elevated during heightened phases of tectonic activity. Over the past 5 million years, enhanced arctic-alpine connectivity facilitated biotic interchange and positioned the boreal-arctic region as a major biogeographic crossroads linking Eurasia and North America.

## Teaser

The asynchronous rise of Northern Hemisphere alpine floras was shaped by general responses to orogeny and climate change.

## INTRODUCTION

How Earth and life have evolved together is an enduring question in biology, motivating us to understand how and why the world’s mountain ranges harbor a disproportionate share of global terrestrial biodiversity (*1*, *2*). Through geological processes such as crustal deformation, surface uplift, and climate-driven erosional feedbacks, mountain systems generate profound topographic and climatic heterogeneity across spatial and temporal scales (*1*–*4*). These geomorphic dynamics have, over millions of years, created complex landscapes that set the stage for the core macroevolutionary processes to build the species richness of a mountain region (fig. S1): in situ diversification, colonization, and local recruitment to regional-specific biomes (*5*). Despite growing recognition of the evolutionary consequences of mountain building (*3*–*7*), the processes by which these diverse biotas were assembled and how assembly was influenced by tectonics and climate change remain unclear.

A key hypothesis is that in situ speciation—the formation of new species within the same geographic region and biome as their ancestors—is accelerated by regional tectonic and geomorphological activity (*4*–*6*). As mountain building progresses, the development of topographic relief can enhance diversification opportunities. However, once a dynamic topographic steady state is reached, rates of in situ speciation may stabilize or decline, even as lineage diversity continues to accumulate (*5*). In tectonically active regions, renewed crustal deformation or intensified climate-driven erosion can reactivate landscape evolution, potentially initiating further pulses of diversification by reshaping ecological gradients and geographic barriers (*3*–*5*). Another important hypothesis is that colonization (the establishment of resident species by dispersal from outside the region) is facilitated by the emergence of geographic or climatic corridors (*8*, *9*). The frequency and directionality of biotic interchange among regions are expected to depend on broader climatic regimes and connectivity patterns (*8*–*10*). The third process by which species can accumulate in a regional biome is local recruitment, in which lineages from adjacent biomes evolve new environmental tolerances (*5*, *11*). Rather than requiring long-distance dispersal, this process typically occurs when environmental change, such as uplift or climatic cooling, expands or shifts local habitat boundaries and species respond adaptively. Quantifying the relative contributions of these evolutionary processes and testing how they have responded to changes in geomorphology and climate are central for understanding the forces that have shaped contemporary distributions of mountain biodiversity.

Biological responses to tectonic uplift can differ depending on the magnitude of topographic development, with erosional highlands expected to exhibit faster and more persistent evolutionary responses compared to adjacent depositional lowlands (*6*). Although fossil-based empirical records serve as valuable proxies for reconstructing regional biodiversity, they are underrepresented in high-relief erosional landscapes and are limited in their ability to resolve fine-scale temporal linkages between tectonic uplift and evolutionary dynamics (*3*, *6*). Distinguishing between speciation, colonization, and local recruitment events, as well as tracking their variation in rate through time and space, is also inherently difficult in the fossil record (*4*). In this context, phylogenetic comparative frameworks that model these processes across biomes and regions provide a spatially and mechanistically explicit means to quantify the tempo and mode of biotic assembly in relation to geomorphic and environmental change.

In this study, we focus on the alpine biome, the ecological zone in mountains that emerges where terrain is elevated above regional climatic treelines, as an ideal system for disentangling the evolutionary consequences of tectonic and climatic forces. Globally, over 70% of alpine plant species are concentrated in mid-latitude mountains of the Northern Hemisphere (*5*, *12*–*14*): the Tibeto-Himalayan-Hengduan (THH) region, the European Alpine system, mountains of Central Asia (Tianshan-Pamir, Caucasus, and the Irano-Turanian Plateau), and the mountains of western North America. Each of these regions has a distinct geomorphological history. High mountains that reached above 4 km were established in southern and central Tibet by the Paleogene (*15*), with sustained high terrain and the progressive development of deeply dissected topography in the Hengduan Mountains throughout the Neogene (*15*–*17*), whereas most other mountain ranges—including the High Himalaya, Tianshan, Alps, and Caucasus—only reached comparable elevations from the Miocene onward (*18*–*27*). In the western North American Cordillera, elevations in the Eocene reached ~4 km, but its modern topography is the result of the Basin and Range extension and mid-Miocene collapse of the highlands (*28*–*31*). These orogenic phases unfolded alongside late Neogene climatic cooling and glacial expansion (*32*), contributing to the expansion of alpine biomes across mountain regions (*12*).

The role of orogenic and climatic forces in creating alpine habitats is widely recognized, but we lack a synthetic understanding of how these forces relate to the timing, modes, and drivers of alpine biotic assembly across mountain regions. For example, the alpine flora emerged in the Hengduan Mountains by the early Oligocene and has persisted since, with pulses of in situ speciation driven by ancient orogeny and intensification of the Asian monsoon that created complex landscapes (*5*). By contrast, the European Alps show a recent rapid accumulation of alpine lineages, indicating a young flora shaped by Pleistocene glacial intensification (*33*). How are the disparate histories of continental alpine floras linked by evolutionary diversification and biotic interchange and the influence of geoclimatic factors on those processes?

Here, we present a quantitative analysis based on time-calibrated phylogenies of 34 clades (8456 species, 55 genera, and 23 families) of flowering plants that broadly represent the most diverse alpine lineages in Northern Hemisphere mountains and the adjacent boreal-arctic region. We jointly inferred geographic range and biome-niche evolution to quantify the relative contributions of in situ speciation, colonization, and local recruitment to alpine floristic assembly. This approach enabled us to connect evolutionary dynamics with regional histories of orogeny and climate change across disparate mountain systems of the Northern Hemisphere. We also constructed a deep-time model of cold-habitat connectivity based on paleoclimate reconstructions to assess the role of climatic cooling in promoting floristic interchange among arctic-alpine habitats. Our approach identifies the key mechanisms that shaped regional biotas and yields general insights into how ancient tectonic events versus recent climatic changes differentially influenced mountain biodiversity patterns.

## RESULTS AND DISCUSSION

### Contrasting evolutionary assembly across alpine floras

**Fig. 1.**
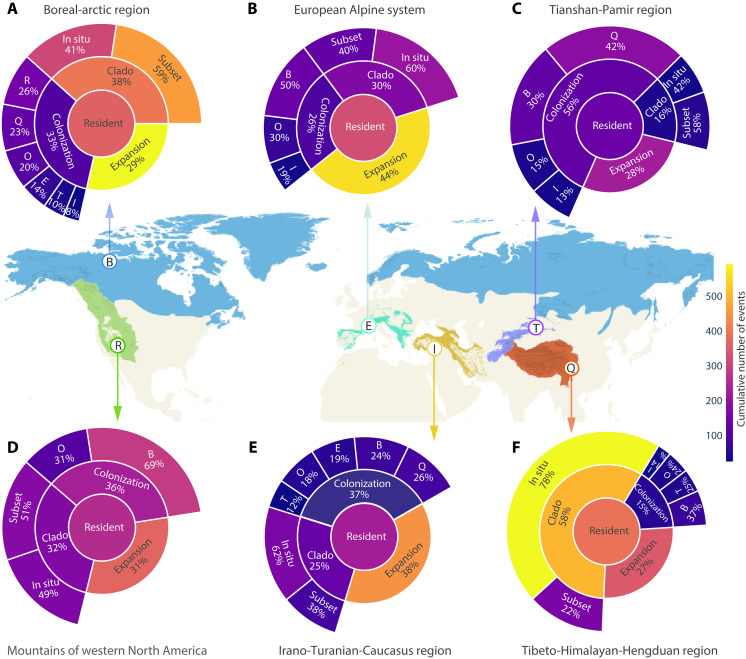
Evolutionary assembly of alpine floras across five major mountain systems and the boreal-arctic region in the Northern Hemisphere. (**A** to **F**) Biogeographic regions showing accumulated cladogenesis, niche expansion (local recruitment across biome boundaries), and colonization events, represented by median values from 1000 replicated joint biogeographic histories. Within each region, biotic assembly through cladogenesis involves in situ speciation, where new alpine species arise from alpine ancestors within the same area, and subset speciation, where new alpine species originate from ancestors spanning multiple alpine biomes. The percentage in each segment (A to F) quantifies the relative contribution of distinct evolutionary processes to the assembly of resident biotas. Outer segments associated with colonization indicate the source regions contributing lineages to the focal region. Spatial distribution of alpine areas (*69*) is mapped as gray shadows within each geographic range.

In this study, we used a joint model of geographic range and biome-niche evolution to infer spatial and temporal rates of fundamental processes governing the assembly of alpine floras: (i) cladogenesis, including in situ speciation (the origin of new alpine species from an alpine ancestor within the same geographic region) and subset speciation (the origin of a new alpine species from an ancestor distributed across alpine and adjacent nonalpine biomes in the same region); (ii) colonization by alpine lineages from other regions; and (iii) niche expansion (local recruitment) of resident lineages across biome boundaries within a region (fig. S1). The differential contributions of these processes can explain observed patterns of regional floristic similarity and distinctiveness across the Northern Hemisphere. In the alpine flora of the THH region, in situ speciation contributed most to biotic assembly over the past 27 million years (Ma) (fig. S2; median age from 1000 replicated biogeographic reconstructions), accounting for 78% of all cladogenesis events (Fig. 1F). The proliferation of alpine endemics via this mode outweighs cases of subset speciation, in which alpine endemics arise from widespread alpine ancestors. Overall, cladogenesis within the region accounts for 57% of alpine assembly events, nearly four times higher than colonization (15%), making the THH region a “speciation engine” fueled by active geodynamics and monsoon-driven diversification (*5*). The disproportionate role of cladogenesis in the assembly process underpins the THH’s exceptional species richness, endemism, and floristic distinctiveness among Northern Hemisphere alpine floras (*34*).

In the European Alpine system, biotic assembly has been dominated by niche expansion of species from lower elevations into the alpine biome (fig. S2), accounting for 44% of biotic assembly events (Fig. 1B). This helps explain the close floristic affinities between the alpine and greater regional temperate floras, in which over half of the common alpine species also occur below the treeline (*35*). The permeability of the alpine-nonalpine boundary in the European Alpine system reflects the relatively shallow ecological gradients that separate biomes in high-latitude mountains (*36*), favoring the preadaption of montane species to the regular freezing and short growing season of the alpine zone during climate fluctuations. In the Irano-Turanian-Caucasus region, niche expansion and colonization codominated alpine assembly, together accounting for ~75% of the total events (Fig. 1E), yielding a distinctive flora characterized by high proportions of species derived from drought-adapted temperate lineages in the regional species pool (*37*) and alpine colonists from adjacent regions. In the Tianshan-Pamir region, colonization dominated the assembly process, primarily involving lineages that migrated from the adjacent, older, and species-rich alpine flora of the THH region, which alone accounted for 43% of all colonization events into this region (Fig. 1C). In the mountains of western North America and the boreal-arctic region, no single process dominated biotic assembly (Fig. 1, A and D). This likely reflects a combination of their broad latitudinal and longitudinal extent, which facilitated colonization and biome expansion during past climatic fluctuations. In addition, the relatively old and tectonically subdued geological history of the western North American ranges may have limited opportunities for in situ diversification.

### Temporal dynamics of biotic interchange in the Northern Hemisphere

**Fig. 2.**
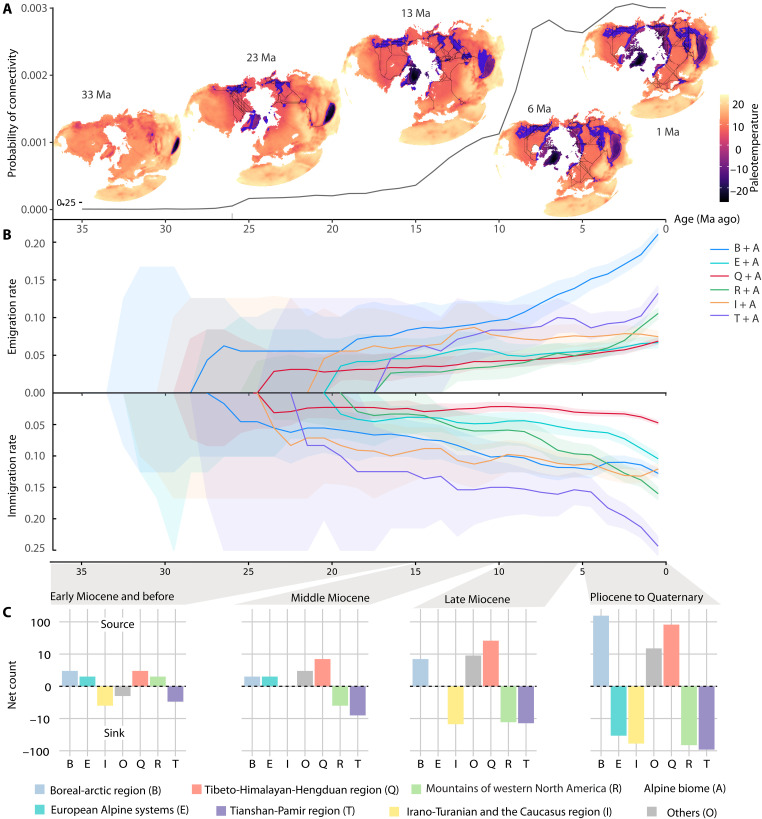
Temporal dynamics of alpine floristic interchange. (**A**) Northern Hemisphere arctic-alpine habitat connectivity over time. Arctic-alpine patches are shown in blue, and least cost paths connecting them are depicted as black lines. Probability of connectivity (PC) is a summary statistic of the overall connectedness of the network defined by arctic-alpine habitat patches (nodes) and least cost paths (edges). (**B**) Rolling estimates of emigration (above) and immigration (below) rates through time for each region. Solid lines indicate median value, and shaded regions indicate the 25 to 75% quantile intervals from 1000 replicated joint biogeographic histories designed to account for the uncertainty of ancestral reconstructions. (**C**) Regional migration balance showing relative differences between emigration and immigration events. Positive values indicate regions that functioned primarily as sources, while negative values represent sink regions. The logarithmic *y* axis enhances visibility across different magnitude scales while preserving proportional relationships between regions and time periods.

Quantification of alpine habitat connectivity over the past 35 Ma (Fig. 2A), based on paleotopographic and paleoclimate reconstructions (*12*), reveals that alpine floristic interchange among Northern Hemisphere regions tracked long-term changes in the availability and spatial continuity of suitable alpine habitats. This interval encompasses the transition from a warmhouse to a coolhouse Earth state following the Mid-Eocene Climatic Optimum (*32*, *38*), which marked a long-term shift toward colder climates that created conditions suitable for the establishment of alpine biotas. The THH and boreal-arctic regions emerged as the earliest sources of alpine lineages dispersing to other Northern Hemisphere mountain systems and continued to be major contributors to alpine floras (Fig. 2, B and C, and fig. S5), reflecting the prolonged history of alpine habitat in these regions (Fig. 2C). By contrast, the Irano-Turanian-Caucasus region, the Tianshan-Pamir region, and the mountains of western North America experienced a sustained net influx of taxa, with immigration exceeding emigration since the middle Miocene (Fig. 2C). Although colonization events occurred in the European Alpine system and the Irano-Turanian-Caucasus region as early as the early Miocene (Fig. 2B and fig. S2), distinct alpine habitats were not established there until the middle to late Miocene (figs. S12 to S19). This discrepancy may reflect the limited power of our paleoreconstructions to detect the earliest small areas of high-elevation habitat that received the first alpine colonizing lineages.

Since the late Miocene, alpine floristic interchange became more frequent, with a notable increase in bidirectional dispersal among regions (Fig. 2C and fig. S6). In particular, emigration from the boreal-arctic region to other mountain systems rose rapidly after 10 Ma, as arctic-alpine habitat connectivity increased among high-latitude ranges (Fig. 2, A and B). By the Pliocene, progressive global cooling further steepened the pole-to-equator temperature gradient (*39*), expanding the extent and connectivity of cold habitats (Fig. 2A). These changes promoted intensified floristic interchange centered on the boreal-arctic region, as evidenced by a marked rise in colonization from this region into adjacent mountain systems across Europe, western North America, and Central Asia over the past 5 Ma (figs. S8 and S9). Floristic interchange was amplified during the Quaternary, when repeated exposures of the Bering Land Bridge during glacial sea level lowstands (*40*, *41*) facilitated both intra- and intercontinental dispersal (Fig. 2, A and B, and fig. S6). The southward expansion of boreal and periglacial floristic elements during glacial periods, together with dispersal corridors established earlier in the Neogene, helps explain the close floristic affinities between the boreal-arctic region and temperate mountain floras across Eurasia and North America (*42*, *43*).

### Orogeny plays a key role in driving alpine plant diversification

**Fig. 3.**
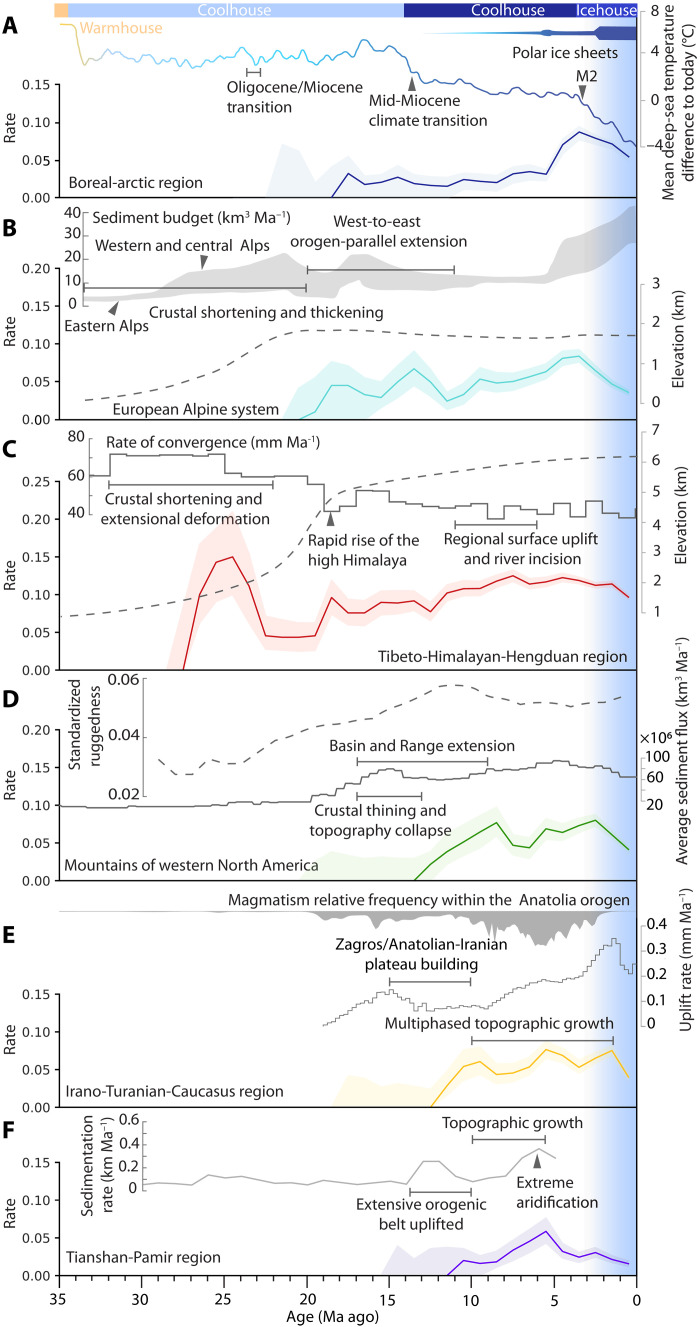
Rates of in situ speciation in relation to climate and geological history across the five major Northern Hemisphere mountain systems and the boreal-arctic region. (**A**) Global surface temperature trends inferred from benthic foraminiferal oxygen isotopes, alongside rough estimates of Northern Hemisphere ice volume (blue bar). M2 marks the first major Northern Hemisphere glacial event (*32*). (**B**) Tectonic activity in the European Alps represented by sediment budgets in the Western (gray-shaded area, top line) and Eastern Alps (bottom line) (*20*), with mean paleoaltimetry estimates (dashed line) (*47*). (**C**) India-Asia convergence velocity, surface elevation history of the Himalaya (dashed line, and episodic tectonic activities in the THH region (*16*–*18*, *79*). (**D**) Sediment flux within the Basin and Range Province reflects western North American tectonics and geomorphic evolution (*28*, *30*, *31*); the uplift history (dashed line) of western North America is reflected by the ruggedness changes (*3*). (**E**) Tectonic and topographic evolution of the Irano-Turanian-Caucasus region as indicated by magmatism and river profile inversion (*26*, *80*). Post–Arabia-Eurasia collision (~15 to 12 Ma) uplifted the Zagros and the Anatolian-Iranian Plateau (*26*, *50*), with subsequent northward shortening after 10 Ma driving multiphased uplift across the region (*24*, *26*, *50*). (**F**) Episodic uplift history in the Tianshan Mountains (*51*–*53*); elevated South Tianshan during 6 to 4 Ma causing extreme aridification in the Tarim Basin around 5.3 Ma (*55*).

Our analysis across clades, regions, and time reveals that the rise and diversification of alpine lineages are closely aligned with periods of orogenic uplift and concomitant climate changes (Fig. 3). In the THH region, the initial burst of alpine diversification around 27 Ma (Fig. 3C) was followed by the onset of local recruitment and colonization of alpine plants (fig. S2). This was temporally congruent with major landscape transformations driven by the southeastward extrusion of the Indochina Block toward the Hengduan Mountains and associated surface uplift (*16*, *17*, *44*), as well as the establishment of a modern-like Asian monsoon system (*45*), together providing a critical setting for the early diversification and ecological adaptation of alpine lineages. The second phase of rapid radiation of alpine lineages occurred from 19 to 17 Ma, during a period of active Himalayan orogeny yielding near-present high elevations above 5000 m (Fig. 3C), corroborating previous analyses of this region (*5*, *18*). Since about 12 Ma, the THH region has sustained elevated rates of in situ speciation—higher than in any other region in our study—through to the present, maintained by the coupled effects of an intensified East Asian summer monsoon and ongoing tectonic activity in the Hengduan Mountains (*5*, *16*, *46*), which act jointly to create greater habitat heterogeneity and ecological opportunities within highly dynamic landscapes.

In the European Alpine system, the rise in the rate of in situ speciation between 20 and 17 Ma (Fig. 3B) followed orogenic widening of the Alps and the accretion of the Aar Massif under ongoing continental collision (*19*, *20*, *47*). Although this extensional phase caused partial topographic collapse, moderate to high elevations were likely maintained, giving rise to complex terrain and environmental gradients that promoted lineage diversification. Alpine plants diversified rapidly during the middle Miocene, with in situ speciation rate peaking around 13.5 Ma (Fig. 3B), concurrent with increased seasonality and a rapid temperature drop by 14 to 13.5 Ma (*20*, *32*, *48*). At this time, the Alps had locally exceeded 4000 m and were characterized by pronounced topographic complexity (*21*). A subsequent pulse of in situ speciation rate around 10 Ma coincided with the northward propagation of uplift and deformation involving the Jura fold and thrust belt (*19*, *20*, *47*). In situ speciation rates peaked again around the Miocene-Pliocene boundary, concurrent with a marked rise in sediment discharge across the Alps (Fig. 3B), indicating intensified surface uplift and erosion. The rapid and regionally extensive uplift around ~5 Ma occurred not only within the Alps but also across much of the circum-Mediterranean orogenic system, fundamentally reorganizing paleogeographic configurations and topography (*19*, *20*). As part of the European Alpine system, the Carpathian and Balkans (Dinarides-Rhodope) were only moderately elevated, rising from near-lowland levels during the middle to late Miocene and continuing into the Quaternary (*19*, *20*). However, these mountains likely did not attain sufficiently high elevations to sustain well-developed alpine habitats, with few, if any, areas comparable to those of the Alps (*20*). This may account for their low diversity of alpine species and the predominance of widespread alpine taxa shared with the Alps (*49*).

The middle to late Miocene marked a pivotal period in the rise and diversification of alpine biodiversity across the major orographic belts of the Northern Hemisphere (Fig. 3). Following a climatic shift from the warm Miocene Climatic Optimum to a notable cooling trend with ice sheet expansion (*32*), pulses of increased in situ speciation rate occurred in Mediterranean orogenic belts and the mountains of western North America. In the Irano-Turania-Caucasus region, the rise of in situ speciation rate since ~12 Ma (Fig. 3E) corresponded with the uplift and deformation across the Zagros/Iranian plateau and the broader Arabia-Eurasia collision zone, which began at 15 to 12 Ma and contributed to increased regional topographic elevation (*50*). In situ speciation rate continued to accelerate toward 5 Ma during multiphased uplift across the Anatolian Plateau, the Alborz, and the Greater Caucasus, associated with the progressive northward transfer of collisional deformation into the continental interior (*22*–*24*, *26*). The latest peak of in situ speciation rate was contemporaneous with an increase in surface uplift around the middle Pleistocene (~1.6 Ma) on the margin of the Anatolian Plateau (*22*, *26*). In the Tianshan region, the onset of alpine plant diversification at ~11 to 10 Ma (Fig. 3F) coincided with a phase of rapid uplift and crustal thickening that culminated by 10 Ma (*27*, *51*–*53*). In situ speciation rate increased from 10 to 6 Ma, as the Tianshan was uplifted by more than 2800 m (*54*), with extreme aridification at 5.3 Ma providing evidence of rain shadows cast by the southern Tianshan (*54*, *55*). In the mountains of western North America, in situ speciation rate increased since ~14 to 13 Ma (Fig. 3D), following the collapse of the Nevadaplano and the lithospheric extension that formed the present-day Basin and Range (*28*–*31*). The development of a sustained alpine biome in this region therefore coincided with the establishment of topographic conditions resembling those of today, and lineage diversification peaked at ~8 to 9 Ma when regional ruggedness was greatest (Fig. 3D).

Across multiple regions, elevated rates of in situ speciation coincided with phases of active geodynamic changes in amplifying topographic heterogeneity and climatic gradients favoring niche differentiation and speciation. In all mountain systems except for the THH region, colonization and local recruitment generally occurred earlier than in situ speciation, likely reflecting a temporal lag between the availability of high-elevation terrain for species establishment and the development of topographic dissection favoring speciation. Thus, the timing of orogeny imposes a strong temporal constraint on the evolutionary assembly of mountain biotas. Whereas climate cooling during the late Miocene set broad ecological conditions, the attainment of critical topographic thresholds through mountain building consistently triggered diversification across the Northern Hemisphere’s alpine floras.

### Recent climate cooling had dual effects on biotic assembly

With increasing high-latitude cooling and polar ice growth, Earth entered a sustained icehouse climate at ~3.3 Ma, followed by intensified glacial-interglacial cycling during the Pleistocene (*32*). These shifts not only expanded arctic-alpine habitats but also amplified glacial erosion, particularly in tectonically active mountain regions (*56*–*58*). Climatically driven erosional processes initially enhanced topographic dissection through valley deepening and cirque formation, but over successive glaciations could also reduce overall relief, depending on latitude, thermal regime, and precipitation patterns (*58*, *59*). These landscape changes, coupled with the expansion of arctic-alpine habitats, likely contributed to a widespread and near-synchronous pulse of in situ speciation during the Pliocene-Pleistocene transition across mid- to high-latitude mountain systems, where sufficient elevations were attained. Notably, in situ speciation rates in the boreal-arctic region began to accelerate around 5 Ma and peaked near 3 Ma, concomitant with the expansion of tundra vegetation documented in the fossil record at that time (*60*). The geographically widespread increase in in situ speciation rates during this period suggests that climate cooling, interacting with regionally heterogeneous landscape changes, promoted alpine plant diversification around the Plio-Pleistocene boundary. However, this trend was not sustained as climatic deterioration intensified during the Pleistocene glacial cycles.

As extensive glaciation expanded across high latitudes during the Pleistocene, in situ speciation rates declined consistently across arctic-alpine biomes of the Northern Hemisphere (Fig. 3), while colonization rates increased, facilitated by the expansion of arctic-alpine habitat corridors (Fig. 2 and fig. S5). Declining speciation was most pronounced at high latitudes, where Pleistocene glaciation exerted stronger and more persistent impacts than in subtropical regions such as the THH, possibly due to the extensive ice cover and repeated glacial overriding that disrupted ecological continuity. At the same time, the dominance of glaciers at high latitudes, frozen to their beds and largely immobile (*58*), greatly reduced erosion and limited the development of rugged, ecologically fragmented terrain, further decreasing opportunities for lineage persistence and diversification. By contrast, climatic oscillations in the tropical northern Andes (*61*) triggered diversification through climate-driven vertical shifts in habitat zones, combined with the persistence of ecological corridors and the development of complex, glaciated terrain that promoted isolation and speciation (*62*). Overall, Pleistocene glacial cycles appear to have played a limited role in stimulating rapid alpine speciation at high latitudes and instead primarily facilitated floristic interchange. This was likely driven by downslope shifts of alpine habitats, which increased habitat continuity and expanded migration corridors across previously fragmented mountain systems and the boreal-arctic region, as reflected in the widespread circumpolar distributions of many genera and species today (*63*). This does not preclude Quaternary speciation altogether but suggests that it was less pronounced than the diversification pulses associated with earlier phases of orogeny. Consequently, studies focused solely on recent timescales may capture only a narrow snapshot of alpine biodiversity dynamics and risk overlooking the effects of deeper-time tectonic and climatic processes on the assembly of alpine floras.

### Summary

The asynchronous and heterogeneous modes by which Northern Hemisphere alpine floras arose reveal general evolutionary responses to orogeny and climate change. Across regions, in situ diversification is primarily driven by tectonic activity and colonization with the expansion of arctic-alpine connections in the late Neogene. These contrasting assembly dynamics help explain today’s notable regional disparities in alpine species diversity and floristic composition. By integrating biological processes and geodynamics across clades, regions, and time, our analysis lays the groundwork for extended predictive models for testing how Earth’s dynamic environment shapes the emergence of biodiversity. As climate and tectonic forces continue to reshape high-elevation environments, understanding the legacy effects of past Earth system interactions will be critical for forecasting the future of mountain biotas and guiding conservation in regions that harbor disproportionate evolutionary history.

## MATERIALS AND METHODS

### Data compilation and phylogenetic reconstruction

We selected 34 angiosperm clades, encompassing some of the most diverse alpine lineages across the five major mountain systems and the boreal-arctic region of the Northern Hemisphere, to cover broad taxonomic and geographic diversity. Fifteen time-calibrated phylogenies were compiled from published literature, and 19 clades were newly reconstructed (table S1). Together, these clades represent 55 genera across 23 angiosperm families, encompassing a total of 8456 species, 2847 of which are found in alpine regions.

DNA sequences used for phylogenetic reconstruction were obtained from GenBank. For each clade, the most commonly sequenced molecular markers were selected to maximize taxon coverage. Sequence alignments were performed separately for each clade using MAFFT v7.22, followed by manual adjustments in Geneious v9.0.5. Ambiguously aligned positions with uncertain homology were trimmed manually, while missing data were retained unless entire sequences were absent, in which case the taxon was excluded. We inferred maximum-likelihood (ML) trees using RAxML HPC2 (v8.2.12) on XSEDE via the CIPRES Science Gateway [General Time Reversible (GTR) + Γ substitution model, 1000 rapid bootstrap replicates]. We used these ML trees to inspect clade monophyly, which informed the topological constraints applied in subsequent time-calibrated Bayesian inference.

Time-calibrated phylogenies were reconstructed using BEAST2 v2.7.3 on XSEDE via CIPRES, applying an uncorrelated log-normal relaxed clock model, the GTR substitution model, and a birth-death prior on tree shape. Parameters and priors were configured in BEAUTi v2.6.6. We used uniform prior distributions to calibrate nodes constrained to be monophyletic, applying fossils to set minimum (younger) bounds and secondary calibrations, where fossil evidence of the clade was unavailable. We assessed Markov chain Monte Carlo (MCMC) convergence in Tracer v1.7.2 after discarding the first 20% of posterior samples, combined log and tree files with LogCombiner, and summarized maximum clade credibility trees with median node heights using TreeAnnotator v1.8.4. Full details of time calibrations are provided in the Supplementary Materials. The types of DNA markers and GenBank accession numbers are provided as separate tables in Dryad (*64*). Time-calibrated phylogenies generated in this study are deposited in the same Dryad archive.

### Delimitation of biogeographic regions

For each biogeographic region, we focused on the alpine species–rich mountain system (*12*), grouping adjacent ranges that share floristic affinities and a relevant geological background. We emphasized the primary centers of alpine diversity within each biogeographic region, aligning them with the major tectonic histories of the respective orogenic systems. The five major Northern Hemisphere mountain systems in our study are classified as follows: (i) the THH region including the Tibetan Plateau, the Himalaya, the Hengduan Mountains (*5*, *15*); (ii) the mountains of Central Asia (Tianshan, Pamir, and Hindu Kush) (*65*); (iii) the mountains of western North America including the Rocky Mountains and adjoining ranges of the Sierra Nevada, Cascade Range, and the Basin and Range Province (*28*–*31*); (iv) European Alpine system, comprising the Alps, Pyrenees, Carpathians, Dinarids, and Balkans following the classification of Kadereit (*49*), that excludes the Apennines in our study, as no alpine species are present there; (v) the Irano-Turanian-Caucasus region, including the Caucasus mountains, the Anatolian highlands, Iranian Plateau and the Zagros mountains (*23*–*26*, *50*). In addition, we delineated the boreal ecozone based on the classification of Bruelheide *et al.* (*66*). The boreal ecozone includes the Arctic and subarctic zone represented by vegetation types of tundra, taiga, and cold steppes.

We downloaded and filtered occurrence data for all species in the selected phylogenies using the R package gbif.range (*67*). We used the CoordinateCleaner package (*68*) to remove problematic records and outliers (excluding zero coordinates and coordinates located at country capitals, country centroids, and biodiversity institutions). We also removed records falling outside the accepted native ranges of species, as defined by the World Checklist of Vascular Plants (WCVP). We used the cleaned occurrences to assign species to mountain regions for subsequent biogeographic analysis. The SpeciesgeocodeR package was used to classify species occurrences to discrete biogeographic ranges, accounting for issues in geopositional data quality. Last, we manually verified each species’ assignment using the WCVP dataset, which provides expert-reviewed taxonomic data for vascular plants. For species lacking occurrence records in Global Biodiversity Information Facility (GBIF) or confirmation in WCVP, we determined their distributions based on published literature and regional floras.

### Delimitation of the alpine (tundra) biome

The alpine zone is defined as a vegetation belt above the climatic treeline and includes portions of the alpine/subalpine ecotone (*69*). As mountain ranges reach into the Arctic region, there is no sharp delimitation between what has been called arctic tundra and the alpine biome, which is exemplified by many “arctic-alpine” plant distributions.

We used multiple strategies to assign species to alpine and nonalpine biomes, considering that limited records for some alpine species above the treeline may bias this delineation. First, our delineation was based on mountain definitions and their classified bioclimatic belts (*70*). These mountain bioclimatic belts are available as a gridded data product, with each cell assigned to one of seven thermal belts at a resolution of 2′30″ (*70*). We combined the thermal belts above the treeline (i.e., “nival,” “upper alpine,” and “lower alpine”) to the “alpine” biome and the remaining thermal belts (“upper montane,” “lower montane,” “freezing,” and “no freezing”) to “nonalpine.” We determined whether species’ occurrences fell within alpine or nonalpine polygons for classification. Second, we determined the assignment of a species to the alpine biome based on each observation point’s vertical distance to the local upper climatic treeline, which represents the lower spatial boundary of the alpine biome. We used altitudinal distance to the treeline (tlh) values estimated from treeline sources to classify species as alpine or nonalpine (*71*). We also extracted the elevation and habitat descriptions with keywords (alpine, tundra, arctic, alpina, glacier, fellfield, treeline, timberline, and tree line) from the cleaned GBIF dataset for each species. We classified species as alpine, nonalpine, or “both,” based on the frequency distribution of their occurrence records across these environments. We applied two filtering thresholds, 5 and 25% of observations in alpine environments, which represent the minimum percentage of observations required for a classification as both or alpine, respectively. In a subsequent step, these assignments were manually cross-checked against floras and, where available, additional information about habitat preferences and elevational ranges.

For species from the European Alps, we validated the classification based on the Flora Alpina (*35*), to which numerous experts contributed. This Flora is an authoritative synthesis across many different national, regional, and local floras, presenting all 4491 vascular plant taxa of the European Alpine Arc, one of the floristically best-known areas in the world. We converted the nival and alpine categories to alpine and the other climatic belts to nonalpine (including the subalpine, montane, and colline belts). We separated subalpine from others because it is a poorly defined transition zone where trees still occasionally occur (*13*). Thus, a species that occurs in subalpine could be assigned to either alpine or nonalpine, depending on the species’ primary habitat preference. We also explored the sPlot (*66*) open dataset to distinguish alpine, polar, and boreal from other biome types. Specifically, we quantified the consistency of our automatic assignments in the European Alps against the independent floristic validation provided by the Flora Alpina monograph. Our results show that the 5% threshold yielded higher consistency than the 25% threshold, whereas the 25% threshold provided higher precision, reflecting a more conservative assignment but substantially underestimating alpine diversity (table S8). Likewise, the treeline-based (tlh) estimate showed higher consistency than the bioclimatic belt (mcb) estimate, although again at the cost of lower precision.

Last, we verified each species using the 25% bioclimatic belt estimation as a conservative assignment and extending to the 5% treeline-based estimation to capture additional potential alpine species. We then assessed whether assignments were consistent across sources, and when discrepancies occurred, we prioritized decisions from local floras or taxonomic publications as the most authoritative references. In cases where no independent resources were available, we applied both the 25% bioclimatic belt and the 5% treeline-based estimations for cross-assessment.

### Biogeographic analyses

We applied an updated biogeographic model (*5*) in which geographic range evolves with species birth (cladogenesis) and death based on the cladogenetic state–dependent speciation-extinction model (ClaSSE). In our model, we set seven free rate parameters representing the speciation rate for each region, along with one between-region speciation rate, one biome transition rate, and local extinction rates. We constrained anagenetic events so that range expansion or contraction and biome shifts could not occur simultaneously (*5*). This means transitions between states that share the same geographic range but differ in being alpine (A) versus nonalpine (a) must occur in two steps via the intermediate state of Aa (occurring in both alpine and nonalpine biomes). The constraint of constant biome occupancy across areas requires assuming that a biome shift occurs in all occupied geographic areas (fig. S1). We also constrained the biogeographic adjacency and set the maximum biogeographic occupancy at three to four regions to ensure the computational feasibility of the model. This constraint was derived from the maximum number of mountain ranges occupied by most of the species (97%) in our study taxa.

We implemented the model and simulations in RevBayes (*72*), using Rev-language functions developed for ClaSSE (*73*). The ClaSSE framework statistically accounts for unobserved speciation and extinction events as well as incomplete taxon sampling, a feature shown to mitigate biases in diversification analyses [e.g., (*73*)]. Clade-specific sampling fractions were specified on the basis of estimates from global and regional floras to correct for differences in taxonomic coverage across groups. Our model extends the ClaSSE framework to incorporate a compound state space encoding both geographic ranges and biome preferences. This yields 150 possible character states, allowing unprecedented resolution in modeling biogeographic dynamics. We used stochastic character mapping (*74*) to generate simulated histories consistent with the observed data from each clade. TensorPhylo plugin was applied to improve the performance of calculating likelihoods with a large state space (all possible region and biome combinations) (*75*). For each clade, we estimated Bayesian posterior distributions using MCMC sampling with proposal tuning during a 200-generation burnin phase, followed by 2000 to 6000 sampling generations depending on the clade analyzed. We retained post–burn-in samples only and ensured that all model parameters achieved effective sample sizes exceeding 200. Parameter sampling checkpoints were saved every five generations to facilitate recovery in the event of interruption. We saved these stochastic mappings as sequences of range and biome states along the branches and at nodes in the phylogeny, such that cladogenetic changes occurring at speciation events were distinguished from anagenetic changes along branches. Using custom Python scripts, we parsed the changes to generate rolling estimates of rates, of range and biome shifts, and of lineage diversification through time.

To enhance model utility and downstream analysis flexibility, we introduced an enriched and standardized output format based on JSON. While the default stochastic mapping format previously represented branch events simply as concatenated state-time strings, the JSON-based representation explicitly records comprehensive event metadata. Each stochastic mapping JSON file enumerates branches (indexed consistently with RevBayes), capturing start and end states and times, and provides a structured list of evolutionary events per branch. These recorded events explicitly categorize cladogenetic events occurring at nodes and along branches and separately identify anagenetic events. In addition, cladogenetic events explicitly record state transitions for both descendant lineages, with each event entry flags whether a state change has occurred. This structured JSON output is generic, highly extensible, and designed to enhance backward compatibility, enabling easier integration with downstream analyses. To efficiently compute likelihoods under thestate-dependent speciation-extinction (SSE) framework, we incorporated the TensorPhylo plugin, which uses a rejection-sampling algorithm akin to Nielsen’s original stochastic mapping method. This implementation enables fast forward simulation of character histories, while accounting for potential unobserved speciation events along branches. Our integrated pipeline, combining ClaSSE inference, structured mapping, and SSE simulation, provides a scalable and transparent framework for dissecting the coupled evolution of species ranges and ecological traits through deep time.

To evaluate the contributions of different evolutionary processes across space and time, we summarized the joint biogeographic histories of range and biome-niche evolution across 34 taxonomic groups using 1000 stochastic mappings sampled randomly from Bayesian posterior distributions to account for ancestral reconstruction uncertainty (*5*). From each region, we extracted the chronology of in situ speciation, subset speciation, colonization, biome transition, and local extinction events. We calculate rolling estimates of per capita in situ speciation rates as $\lambda \left(t\right)=\frac{s\left(t\right)}{n\left(t-1\right)}$, where *s*(*t*) is the number of in situ speciation events inferred in a region in a 1-Ma period *t* and *n*(*t* − 1) is the number of inferred lineages in the region in the previous period (the cumulative sum of in situ speciation, subset speciation, biome expansion, and colonization minus local extinction). We calculated rolling per capita rates of colonization between regions as $d_{\mathrm{ij}}\left(t\right)=\frac{c_{\mathrm{ij}}\left(t\right)}{n_{i}\left(t-1\right)}$, where $d_{\mathrm{ij}}\left(t\right)$ is the number of inferred colonization events of area *j* from area *i*. The same approach was applied to other processes. For all estimates, we plotted the median value and shaded areas indicate the 25 to 75% quantile intervals through time from 1000 replicated joint biogeographic histories. We implemented a masking strategy for each process and quantile (median, 25th, and 75th) that sets rate values to zero before the last occurrence of zero within each time series. This procedure was applied independently to each quantile trajectory to reduce noise in early ancestral reconstructions and to better represent the continuous evolutionary history of the extant alpine biome. Biotic interchange events between regions during specific geological periods were quantified using the median value of cumulative sums derived from 1000 replicated biogeographic histories (*5*). These values were calculated by summing colonization events across consecutive 1-Ma time windows throughout the target period for each region.

### Calculating connectivity among alpine-arctic habitats in the Northern Hemisphere

We calculated global connectivity based on paleo-topoclimate reconstructions at million-year intervals over the past 35 Ma (*12*) using a network-based approach (*76*–*78*). This period was chosen to capture the broad emergence and expansion of cold climates following the Mid-Eocene Climatic Optimum, marking the transition from a warmhouse to a coolhouse state as long-term global cooling began (*32*). At each time step, we first cropped the longitude/latitude grids of the paleoclimatic reconstructions to cover the focal Northern Hemisphere mountain systems (10°N to 90°N) and then reprojected them to North Pole Lambert azimuthal equal area projection [European Petroleum Survey Group (EPSG): 17299] with a horizontal resolution of 50 km, using bilinear interpolation. Next, we rounded temperatures of each pixel to whole degrees and defined the alpine biome as those pixels with annual mean temperatures ranging from 0 to 6°C. Then, using Graphab (*76*, *77*), we calculated structural connectivity for the network of alpine biome patches. We defined the resistance layer, which needs to be overcome to connect these patches, based on the absolute temperature deviations from those temperatures classified as the alpine biome. Graphab then identified least cost paths between any two alpine biome patches and established them as links, whenever the cumulative resistance along the least cost path was less then 250, which roughly corresponded to 70,000-km Euclidean distance (inferred from the 10-Ma network). As a global metric of the structural connectivity of the resulting network, we used the probability of connectivity (PC) (*78*), which measures the probability that two random pixels belonging to the alpine biome are interconnected. To calculate PC, we assumed the probability of movement to exponentially decay to 0.05 at a cost distance of 150, corresponding to roughly 4200-km Euclidean distance (inferred from the 10-Ma network). This approach calculates ecological resistance solely based on temperature differences and ignores physical barriers such as glaciers, ice sheets, or shifts in coastlines due to sea level change. While these obstacles may be relevant at shorter timescales, the million-year time steps considered here are far longer than glacial-interglacial cycles and thus our assessment smooths over these effects. To depict the long-term impact of the global cooling on the connectedness of alpine-arctic biomes, however, we believe our parsimonious approach is well suited.

## Supplementary Materials

### This PDF file includes:

Supplementary Text Figs. S1 to S41 Tables S1 to S8 References
