# Supplementary material for: The asynchronous rise of Northern Hemisphere alpine floras reveals general responses of biotic assembly to orogeny and climate change
Source: Sci Adv. 2025 Dec 19;11(51):eadz1888. doi: 10.1126/sciadv.adz1888 (PMC12716423; doi:10.1126/sciadv.adz1888)
Supplement: Supplementary file 1 — Supplementary Text Figs. S1 to S41 Tables S1 to S8 References [file sciadv.adz1888_sm.pdf]

Science Advances
AAAS

Supplementary Materials for

The asynchronous rise of Northern Hemisphere alpine floras reveals general responses of biotic assembly to orogeny and climate change

Wenna Ding *et al.*

Corresponding author: Wenna Ding, dingwenna@xtbg.ac.cn; Richard H. Ree, rree@fieldmuseum.org; Yaowu Xing, ywxing@xtbg.org.cn

Sci. Adv. **11**, eadz1888 (2025)
DOI: 10.1126/sciadv.adz1888

This PDF file includes:

Supplementary Text
Figs. S1 to S41
Tables S1 to S8
References

Supplementary Text

Phylogeny and molecular dating: time calibrations and topological constraints

For each clade, the ingroup comprised taxa targeted for the biogeographic analysis, while the outgroup consisted of closely related taxa used to root the phylogenetic trees. The broader "outgroup scope" was defined as the least-inclusive clade containing both ingroup and outgroup taxa. Uniform prior distributions were applied to calibrated node ages, except where noted, with minimum (younger) bounds set using fossil evidence wherever possible. For eudicot angiosperm nodes lacking more specific constraints, a conservative maximum (older) bound of 125 Ma—the age of the earliest known eudicot fossil—was applied (*81*). When reliable fossils were unavailable, we used secondary calibrations derived from published fossil-calibrated phylogenies of related clades. The DNA markers used to construct dated phylogenies and the justification of node age constraints applied for divergence-time analyses are provided below.

In group Caryophyllaceae; outgroup scope: Caryophyllales

DNA markers were used to construct a dated phylogeny: one nuclear marker (ITS) and four plastid markers (matK, rbcL, rps16, trnL-trnF).

Crown age of Caryophyllaceae: 48.6–125 Ma. The lower bound was constrained based on the inflorescence fossil of *Caryophylloflora paleogenica* from the late middle Eocene fossil, which was placed within Caryophyllaceae, either subfamily Alsinoideae or Caryophylloidea. This relatively simple pollen type first appeared in Australia and New Zealand in the Campanian as the oldest known record of the Caryophyllaceae (*82*). The upper (older) bound 125 Ma adopted the Caryophyllales crown age inferred from the fossil-calibrated angiosperm phylogeny that represents 85% of extant families and all orders (*83*).

Stem age of *Silene*: 11.6–125 Ma. The lower bound age was constrained based on the earliest fossil record of *Silene*, *S. novorossica* from the late Miocene, Odessa region, Ukraine (*84*).

Stem age of *Moehringia*: 23.03–125 Ma. The lower bound age was constrained based on the earliest fossil record *M. miocaenica* from late Oligocene of Europe (*85*).

Stem age of *Stellaria*: 5.33–125 Ma. The lower bound age was constrained based on the earliest fossil record of *Stellaria*, *S. media* from the late Miocene of Asia a nd Europe (*86*).

Ingroup: Juncaceae; outgroup scope: Poles

DNA markers were used to construct a dated phylogeny: one nuclear marker (ITS) and three plastid markers (matK, rbcL, trnL-trnF).

Root age (the crown age of Poales): 100–158 Ma. The lower bound was constrained based on the earliest known grass fossils (silicified epidermal pieces and phytoliths) with affinity to Poaceae from the late Early Cretaceous (Albian, 113–101 Ma) of the Mazongshan area in northwestern China (*87*).

Crown age of Juncaceae+ Cyperaceae: 47–100 Ma. The lower bound was constrained based on the fossil cyperacean infructescences (*Volkeria messelensis*) with *in situ* pollen from the Middle Eocene (Geisaltalian, 47 Ma) of Messel, Germany (*88*).

Crown age of Juncaceae: 33.9 Ma–98 Ma. The lower bound was constrained based on fossil plants of Juncaceae found from New Zealand since the Oligocene (*89*). The upper bound is inferred from big angiosperm phylogenies and fossil-based estimation of angiosperm ages (*83, 90*).

Crown age of *Carex* +*Cyperus*: 15.97–98.0 Ma. The lower bound was constrained by the oldest fossil record of *Carex* from Early Miocene Lübbenau, Brandenburg, Germany (*91*). The upper bound of the family age was inferred from big angiosperm phylogenies and fossil-based estimation of angiosperm ages (*83, 90*).

Ingroup: *Ranunculus*; outgroup scope: Ranunculoideae

DNA markers were used to construct a dated phylogeny: one nuclear marker (ITS) and three plastid markers (matK, psbJ-petA, rbcL).

The constraints of the *Ranunculus* were derived from the 95% HPD (highest posterior density) of node age inferred from the fossil-calibrated backbone of Ranunculaceae (*92*).

Root age: 63.44–75.08 Ma.

Crown age of *Ranunculus*: 20.33–31.88 Ma.

Ranunculus +*Krapfia*+ *Laccopetalum*: 24.37–36.33 Ma.

Crown age of Ranunculeae: 42.72–54.92 Ma.

Ingroup: *Festuca*; outgroup scope: Poeae

DNA markers were used to construct a dated phylogeny: one nuclear marker (ITS) and three plastid markers (matK, rbcL, trnL-trnF).

The constraints of the *Festuca* are derived from the 95% CI intervals inferred from the fossil calibrated backbone of Pooideae (*93*).

Root age (crown age of Poeae): 28.7–34.9 Ma.

Crown age of Loliinae: 17.5–22 Ma.

Crown age of Loliinae +Parapholis: 21.9–24.8 Ma.

Ingroup: *Poa*; outgroup scope: Poeae

DNA markers were used to construct a dated phylogeny: one nuclear marker (ITS) and three plastid markers (matK, rbcL, trnL-trnF).

The constraints of the *Festuca* are derived from the 95% CI intervals inferred from the fossil-calibrated backbone of Pooideae (*93*).

Root age (crown age of Poeae): 28.7–34.9 Ma.

Crown age of *Poa*+*Phleum*: 19.2–19.6 Ma.

Crown age of *Poa*+*Phleum* +*Alopecurus*+*Apera*+*Beckmannia*+*Milium*+*Deschampsia*: 26.3–26.6 Ma.

Ingroup: Potentilleae; outgroup scope: Rosoideae

DNA markers were used to construct a dated phylogeny: one nuclear marker (ITS) and three plastid markers (matK, rbcL, trnL-trnF).

Root (crown age of *Rosa* + Potentilleae): 48.6–100 Ma. The lower bound is constrained based on the oldest fossil record of *Rosa* (*R. germerensis*) from the Eocene (55.8–48.6 Ma) in North America (*94*). The upper bound is based on the maximum node age of Rosaceae from the fossil-calibrated angiosperm phylogeny that represents 85% of extant families and all orders (*83*).

Crown age of *Potentilla*: 11.6–100 Ma. The lower bound is based on fossils of *Potentilla* from brown coal strata in Lausitz, Germany, formed in Early–Middle Miocene (11.6–23.0 Ma) (*95*). The oldest *Potentilla* fossil is reclassified by Wolfe and Schorn (*96*) from the Creede Flora in North America, but it was disputed by Töpel et al. (2012) after examining this fossil (*97*). Therefore, the younger European fossil was adopted for calibrating the genus.

Alchemilla +*Schistophyllidium*: offset 24.1 Ma, mean 1, sd 1 (*98*).

Drymocallis +*Chamaerhodos*: offset 15.1 Ma, mean 1, sd 1 (*98*).

Crown age of tribe *Kerriae*: 49.4–125 Ma. The lower bound is based on the earliest fossil record for the tribe, assigned to the genus *Neviusia*, from the middle Eocene of British Columbia (*99*).

Ingroup: Trifolieae; outgroup scope: Fabaceae

DNA markers were used to construct a dated phylogeny: one nuclear marker (ITS) and four plastid

markers (matK, rbcL, trnL, trnL-trnF).

Ingroup of Trifolieae applied a secondary calibration with a median of 24.7 and a standard deviation of 2.3 (*Trifolium* + *Ononis*) (*100*). Similarly, the root age (*Trifolium*+ *Astragalus* + *Glycyrrhiza*) was constrained with a mean of 39 and a standard deviation of 2.4 (*100*).

Ingroup: Caraganeae; outgroup scope: Fabaceae

DNA markers were used to construct a dated phylogeny: one nuclear marker (ITS) and five plastid markers (matK, psbA-trnH, rbcL, trnL-trnF, trnS-trnG).

Secondary calibration was applied based on the previous study (*100*) in which many Fabaceae fossils were used as constraints. This calibration was implemented with a normal distribution and the central 95% ranges were given as follows (*100*).

Root age (*Caragana*+ *Sesbania*): 49.1–52.1 Ma.

Caragana+ *Hedysarum*: 24.4–34.2 Ma.

Caragana+ *Astragalus*: 28.6–37.4 Ma.

Ingroup: *Galegeae*; outgroup scope: Fabaceae

DNA markers were used to construct a dated phylogeny: one nuclear marker (ITS) and three plastid markers (matK, rbcL, trnL-trnF).

There are no available fossils in *Oxytropis* and related genera (*Astragalus*, *Colutea*, etc.) that can be used for time-calibration. Secondary calibration was applied from a previous study (*100*). This calibration was done with a normal distribution following the original results.

Root age (*Glycyrrhiza*+*Trifolium*: 39 Ma, standard deviation of 2.4.

Crown age of *Colutea*+*Astragalus*: 14.8 Ma, standard deviation of 2.

Crown age of *Oxytropis*+*Astragalus*: 12.3–20.7 Ma (95% HPD) (*101*).

Ingroup: *Campanula*; outgroup scope: Campanuloideae

DNA markers were used to construct a dated phylogeny: one nuclear marker (ITS) and four plastid markers (matK, petD, rbcL, trnL-trnF).

The time-constraints applied to this secondary calibration under a uniform prior were selected based on the estimated age and 95% confidence intervals (CIs) of clades resolved as sister to *Campanula* s.l. in (*102*) and adjusted from (*103*).

Root age: 50–63 Ma.

Crown age of *Campanula*: 24.8–47.9 Ma.

Ingroup: Liliaceae; outgroup scope: Liliales

DNA markers were used to construct a dated phylogeny: one nuclear marker (ITS) and five plastid markers (matK, psbA-trnH, rbcL, rpl16, trnL-trnF).

Stem age of Liliaceae: 100–171.7 Ma. The upper bound of the stem age was constrained by the node age of Liliales estimated from phylogenetics trees of 80 plastid genes (*83*), and the lower bound was constrained by the lower bound of Liliales splitting into its constituent families (*104*).

Crown age of *Calochortus*: 13.9–32.96 Ma (*105*).

Crown age of Lilieae: 24.5–50.2 Ma (*105*).

Crown age of Medeoloideae: 22.52–58.94 Ma (*105*).

Crown age of Tupileae: 39.5–66.9 Ma (*105*).

Ingroup: *Salix*; outgroup: Salicaceae

DNA markers were used to construct a dated phylogeny: one nuclear marker (ITS) and four plastid markers (matK, rbcL, rpoB-trnC, trnL-trnF).

The upper bound was constrained by the split age of Lacistemataceae+Salicaceae estimated from phylogenetics trees of 80 plastid genes (*83*), consistent with the age estimated from fossils dataset (*90*).

Stem age of *Pupulus* and *Salix*: 48–74.8 Ma. The lower bound is based on the early Middle Eocene foliage and fruits *Populus tidwellii* from North America, which have characterized stem lineages within the *Populus* and *Salix* clade (*106*).

Crown age of *Salix*: 23–74.8 Ma. The lower bound was constrained based on the earliest reliable *Salix* fossils with both catkins and leaves from the late Oligocene in Alaska (*107*).

Ingroup: *Nepeta*; outgroup: Nepetinae

DNA markers were used to construct a dated phylogeny: one nuclear marker (5.8S) and five plastid markers (matK, rbcL, rpl32-trnL, trnL-trnF, ycf1).

To estimate divergence times, we constrained multiple nodes using secondary calibration points taken from 95% highest posterior density (HPD) for major clades of Lamiaceae (*108*) and (*109*) with uniform priors.

Root (crown age of tribe Nepetinae): 26.2–39.5 Ma.

Crown age of *Nepeta*+*Lophanthus*+*Drepanocaryum*: 10.5–24.4 Ma.

Crown age of *Agastache*+*Dracocephalum*+*Glechoma*+*Meehania*: 18.23-31.0 Ma.

Ingroup: *Crocus*; outgroup: Iridaceae

DNA markers were used to construct a dated phylogeny: one nuclear marker (5.8S) and five plastid markers (matK, ndhF, rbcL, rpoC1, rps4).

We used posterior age estimates (95% highest posterior density (HPD) intervals) from the secondary calibration of (*110*) to constrain three nodes with normally distributed priors.

Root age (the crown node of Iridaceae): 60.6–82.9 Ma. The crown node of Iridaceae referred to the divergence of *Isophysis* from the rest of the other genera.

Crown age of Crocoideae + Nivenioideae+Aristeoideae+*Geosiris*: 42.2–64.5 Ma. *Geosiris* diverged from its sister clade (Aristeoideae, Nivenioideae and Crocoideae) in the early Eocene.

Crown age of Crocoideae + Nivenioideae: 27.6–46.5 Ma.

Crown age of *Iris*+*Moraea*+*Ferraria*+*Diete*s: 23.3–50.7 Ma.

Crown age of *Herbertia*+*Solenomelus*: 35.4–57.3 Ma.

Ingroup: *Stipa*; outgroup: Stipodae

DNA markers were used to construct a dated phylogeny: one nuclear marker (5.8S) and four plastid markers (matK, ndhF, rbcL, trnL-trnF).

The constraints of the *Stipa* were derived from the 95% CI intervals inferred from the fossil calibrated backbone of Pooideae (*93*). We also constrained the tree topography based on the phylogeny of Pooideae from five trees inferred by ASTRAL (*93*).

Root age (crown age of Stipodae): 36.8–37.2 Ma.

Crown age of *Nassella*+*Jarava*: 10.3–10.6 Ma.

Crown age of *Stipa*: 12.5-13.0 Ma; or stem age of *Stipa*: 12.5–30.2 Ma.

Sensitivity analysis

The effect of phylogenetic uncertainty in tree topology and branch lengths across multiple trees per clade was not explicitly evaluated in our biogeographic analyses. Instead, we focused on integrating over uncertainty associated with inferring when, where, and how ancestral transitions in range and biome affinity, and speciation mode, occurred on phylogenetic trees. To do so, we used Bayesian stochastic mapping to generate posterior samples of candidate histories, from which we derived region-specific inferences of rate variation through time (see MATERIALS AND METHODS). Owing to the computational burden of generating these posterior samples, we thus did not integrate over phylogenetic uncertainty in tree topology or branch lengths, and instead based our inferences

on the maximum clade credibility (MCC) tree for each focal clade. Importantly, we did not rely on a single phylogeny: 34 clades were analyzed independently, each with its unique evolutionary history. This multi-clade design reduces the influence of potential biases in any one clade and integrates across a diversity of evolutionary trajectories, thereby mitigating phylogenetic uncertainty at the macroevolutionary scale.

To further evaluate robustness to incomplete sampling bias, we conducted sensitivity analyses by randomly subsampling two of the best-sampled clades (*Campanula* and *Saxifraga*) to 50% and 20% of their total diversity (fig. S3; fig. S4). These reduced datasets yielded reconstructed biogeographic histories and rate estimates that closely matched those from the full analyses (fig. S2). The core results regarding the dominant evolutionary processes, their relative importance across regions, and pulses of *in situ* speciation rate change remained unchanged. Only subtle temporal shifts were observed: an earlier onset of biome expansion in the boreal–arctic region under 50% sampling, and in both the boreal–arctic region and the European Alpine system under 20% sampling. These results indicate that reduced sampling does not meaningfully affect our inferences and provide reassurance that our conclusions are robust despite variation in sampling coverage across clades.

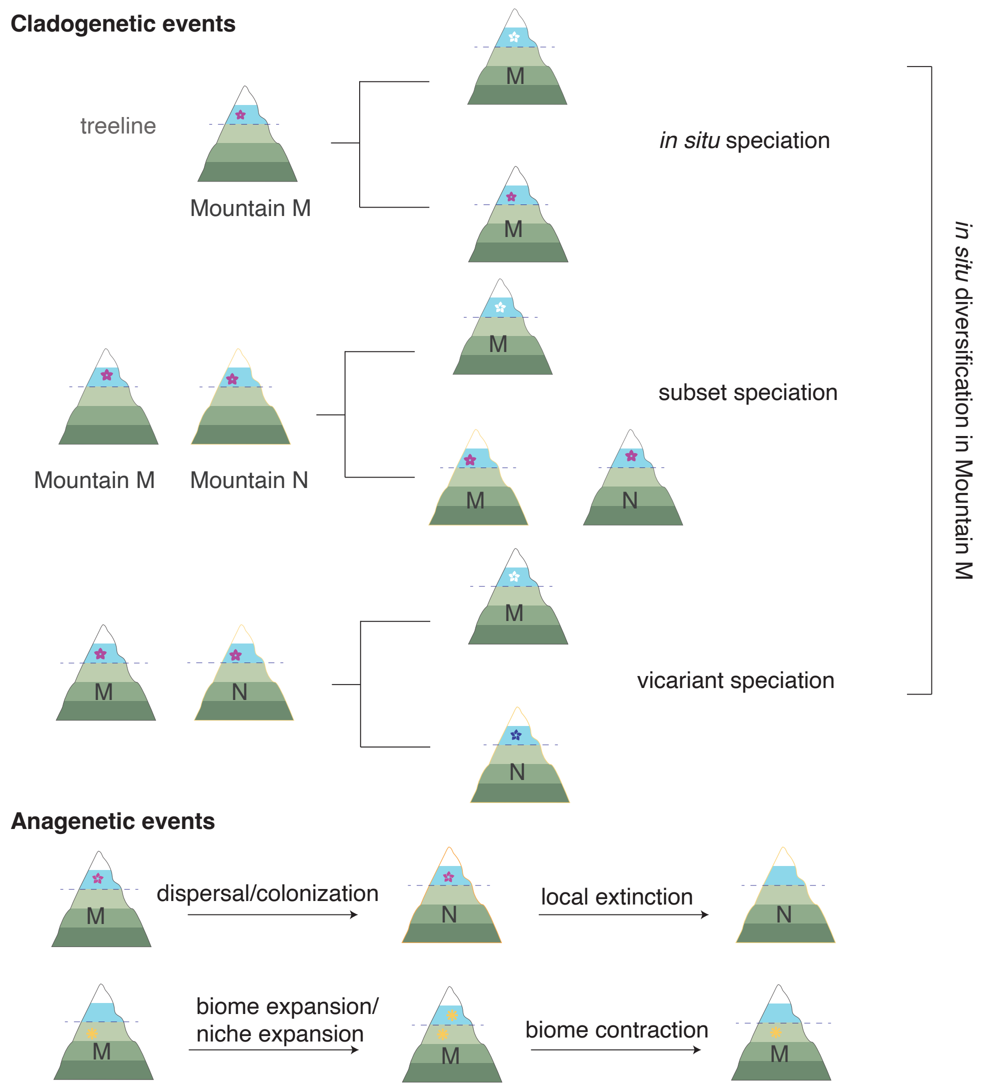

Figure S1: **Schematic illustration of cladogenetic and anagenetic processes included in the joint geographic range and biome-niche evolution model.** The mountain shown represents a relatively broad mountain system consistent with our study. Cladogenetic changes occur at speciation events. *In situ* speciation refers to the speciation that occurs *in situ* when the ancestral range contains one area, both daughter lineages inherit that area. Subset speciation occurs when the ancestral species occupies wide geographic ranges or cross biomes that give rise to two daughter species, in which one retains the full ancestral range or niche while the other becomes restricted to a specific subset of the ancestral range. Vicariant speciation refers to one daughter lineage inheriting a subset of the ancestral areas while the other daughter inherits all remaining ancestral areas, but this process adds no more species to each of the geographic pools. Anagenetic changes occur along the branches of a phylogeny, within a lineage. There are three types of anagenetic events, dispersal, (local) extinction, and biome expansion/contraction.

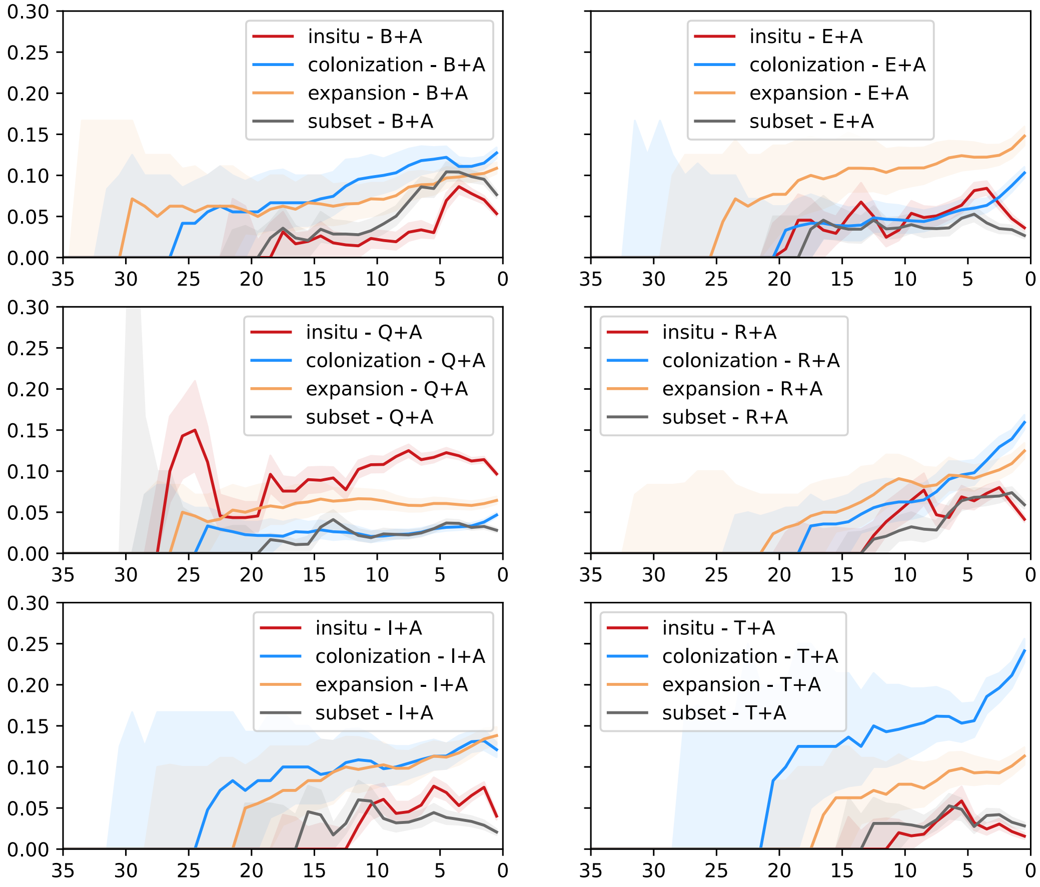

Figure S2: **Assembly history of alpine floras across the Northern Hemisphere and rolling estimates of associated rates through time.** Solid lines show the median estimates, while shaded bands represent the interquartile range (25th–75th percentiles) based on 1,000 replicated biogeographic histories that incorporate uncertainty in ancestral reconstructions. To reduce noise in early ancestral reconstructions and reflect the continuous history of the alpine biome, rates were masked to zero before the last absence. B = Boreal-arctic region, E = European Alpine system, Q = Tibeto-Himalayan-Hengduan region, R = Mountains of western North America, T = Tianshan-Pamir region, I = Irano-Turanian-Caucasus region, A = the alpine biome.

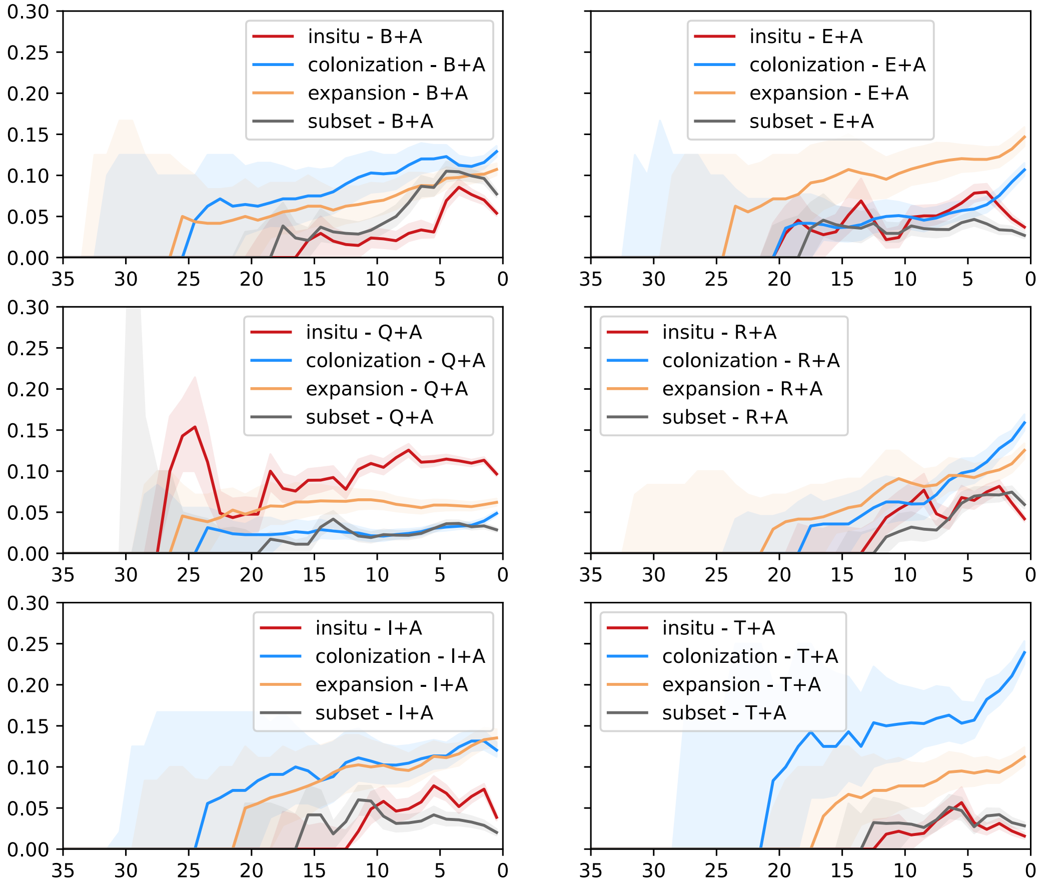

Figure S3: **Assembly history of alpine floras across the Northern Hemisphere and rolling estimates of associated rates through time.** For this sensitivity analysis, *Campanula* and *Saxifraga* were randomly resampled to 50% of their original diversity, while all other clades were kept unchanged. Solid lines show median estimates, and shaded bands represent the interquartile range (25th–75th percentiles) based on 1,000 replicated biogeographic histories that incorporate uncertainty in ancestral reconstructions. To reduce noise in early ancestral reconstructions and to reflect the continuous history of the alpine biome, rates were masked to zero prior to the last absence. Labels follow those in fig.S2.

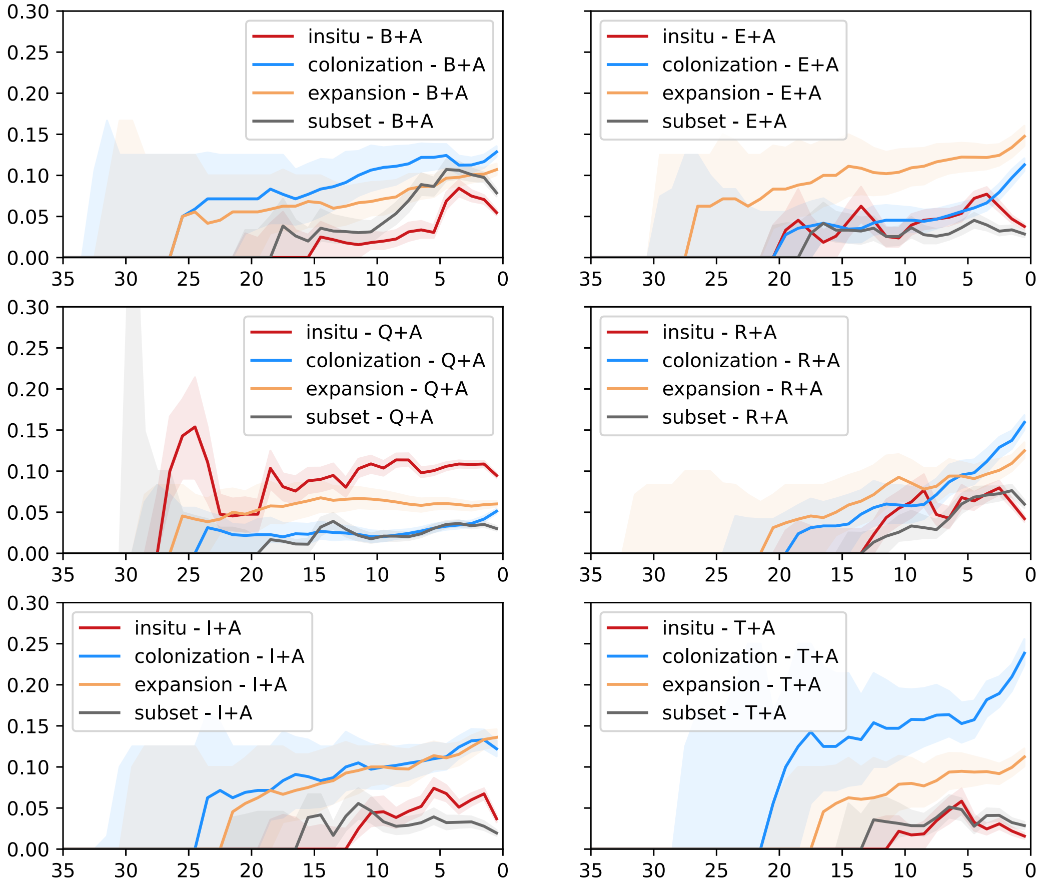

Figure S4: **Assembly history of alpine floras across the Northern Hemisphere and rolling estimates of associated rates through time.** For this sensitivity analysis, *Campanula* and *Saxifraga* were randomly resampled to 20% of their original diversity, while all other clades were kept unchanged. Solid lines show the median estimates, while shaded bands represent the interquartile range (25th–75th percentiles) based on 1,000 replicated biogeographic histories that incorporate uncertainty in ancestral reconstructions. To reduce noise in early ancestral reconstructions and reflect the continuous history of the alpine biome, rates were masked to zero before the last absence. Labels follow those in fig.S2.

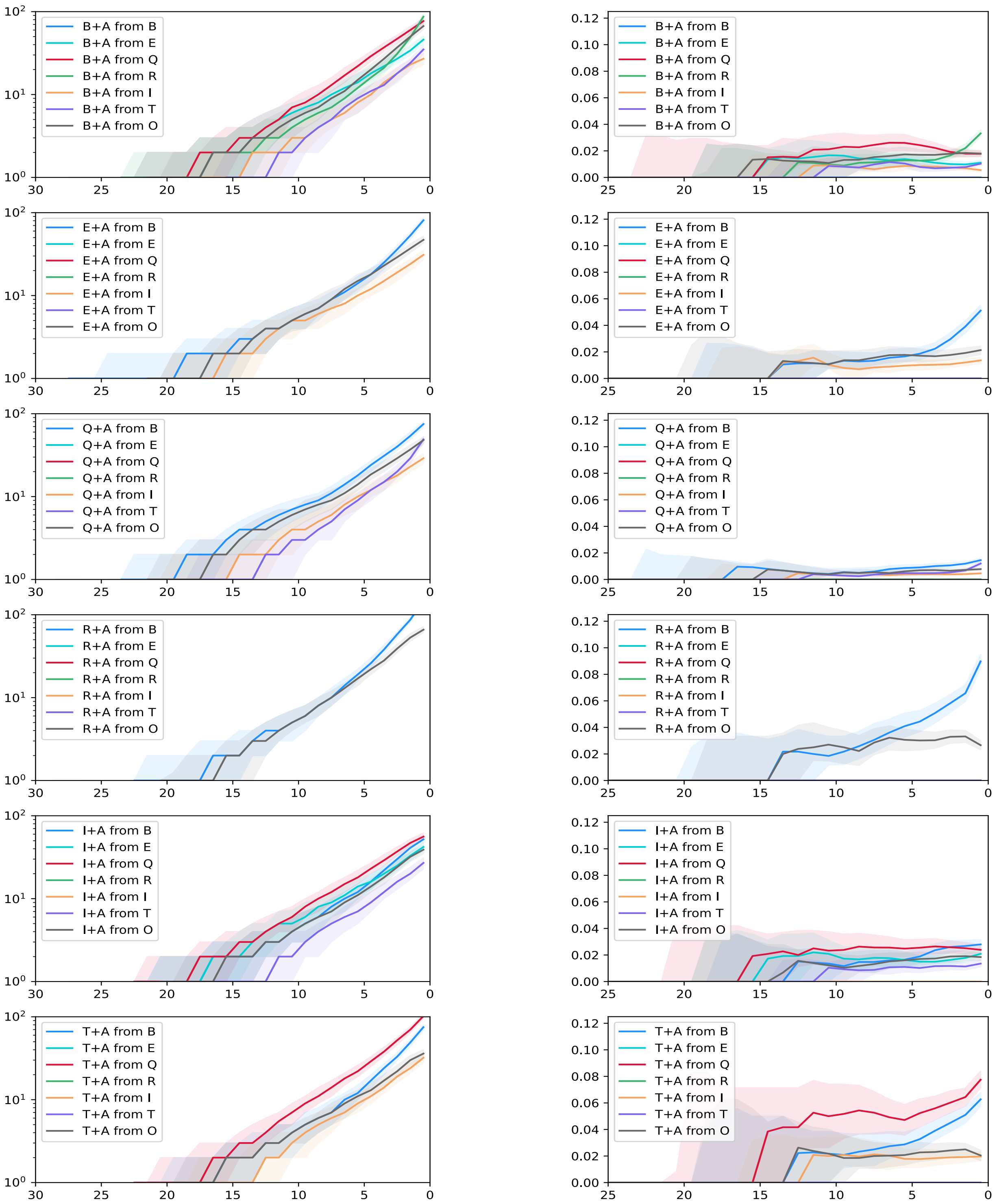

Figure S5: **Cumulative colonization events (left) and rolling estimates of colonization rates (right) from each region through time**. Solid lines show the median estimates, while shaded bands represent the interquartile range (25th–75th percentiles) based on 1,000 replicated biogeographic histories that incorporate uncertainty in ancestral reconstructions. Labels follow those in fig.S2, and O = Other regions.

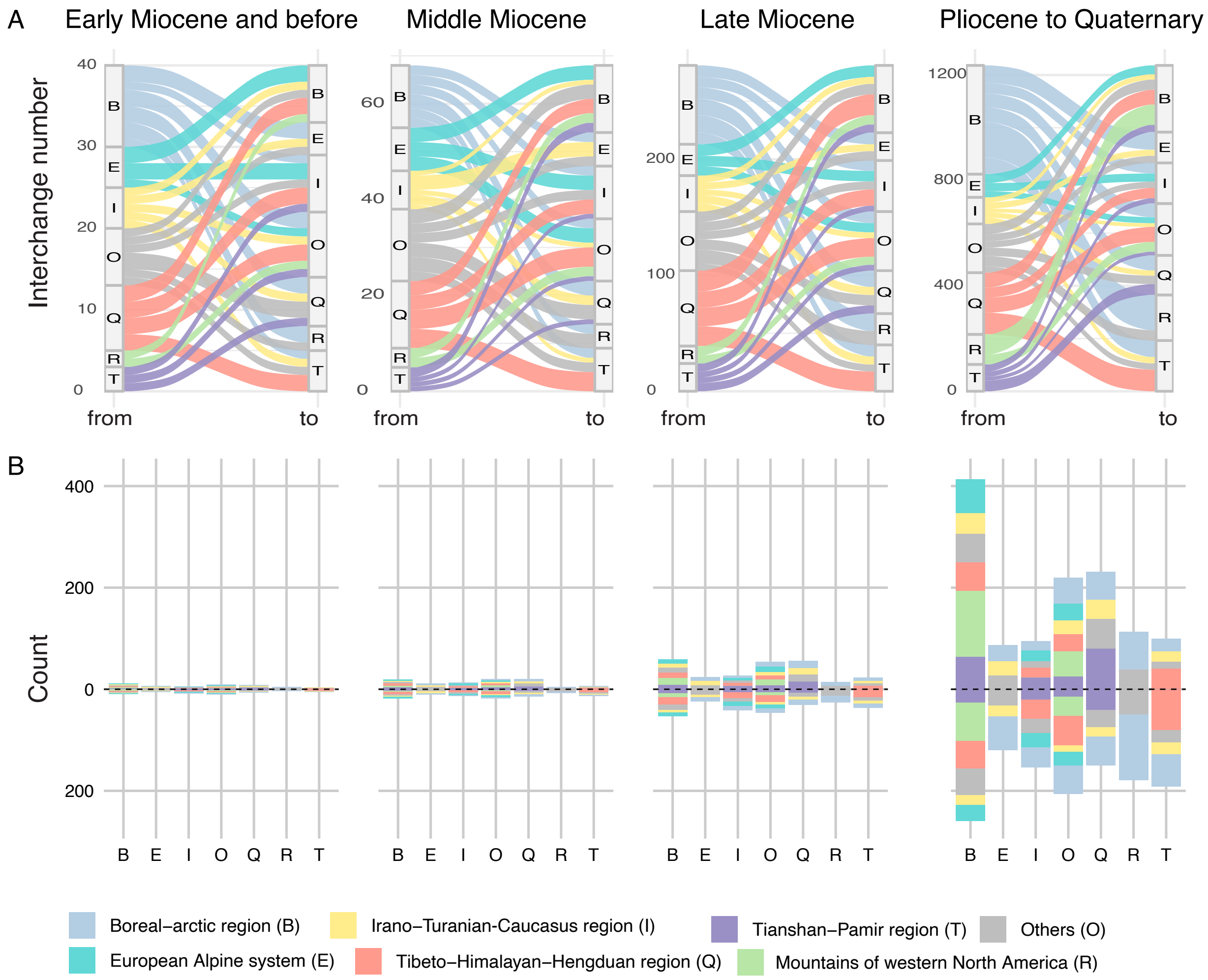

Figure S6: **Temporal dynamics of biotic interchange across major mountain systems and the boreal-arctic region in the Northern Hemisphere.** A. Arctic-alpine plant dispersal from source regions (left) to recipient regions (right) across four key geological intervals. Flow line widths are proportional to total dispersal events for each time period. B. Stacked bar plots show the cumulative number of emigration (above dash line) and immigration (below dash line) events between regions across four geological time periods. Each bar represents a focal region (x-axis), with colored segments indicating the contributing source or recipient regions. Horizontal dashed lines mark zero, separating emigration (above) from immigration (below). These diagrams highlight the increasing intensity and complexity of interregional alpine plant dispersal over time, with especially pronounced exchange involving the boreal-arctic region during the Pliocene–Quaternary.

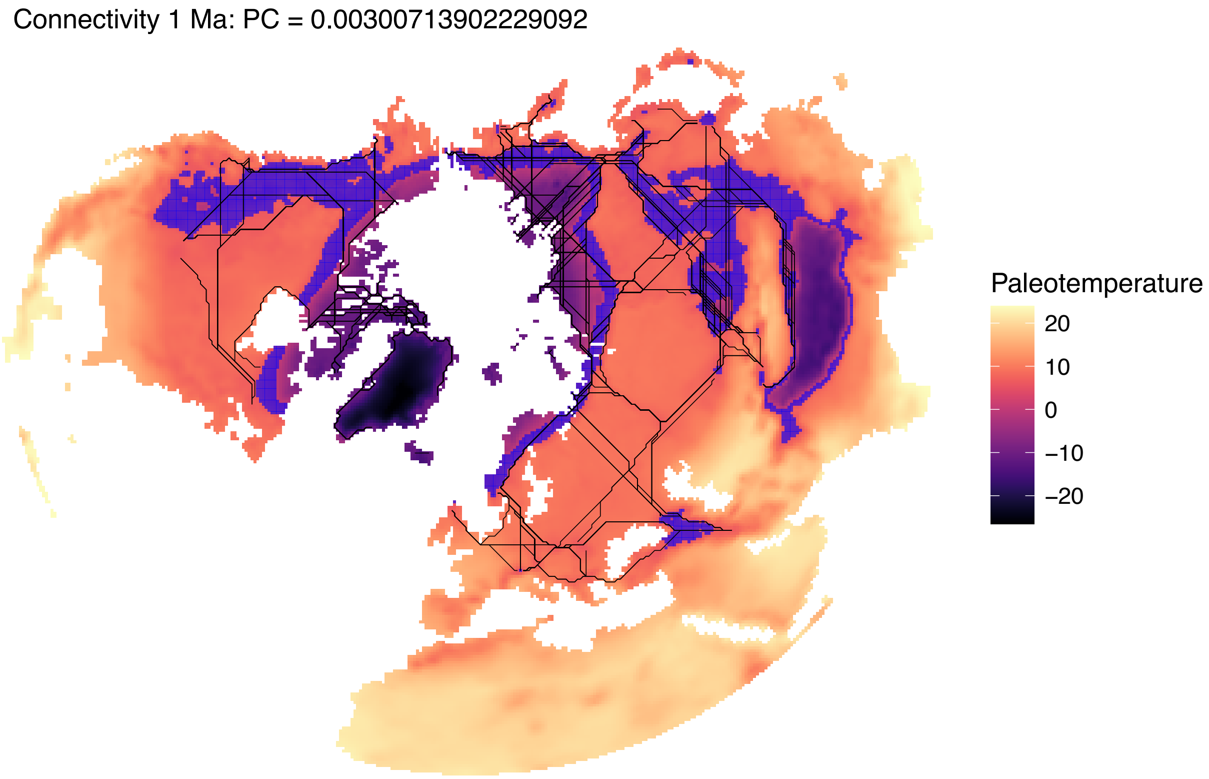

Figure S7: **Estimated distribution of arctic–alpine habitats and their connectivity across the Northern Hemisphere at 1 Ma.** Arctic-alpine habitats are shown in blue, and least cost paths connecting them are depicted as black lines. PC refers to probability of connectivity, which is a summary statistic of the global connectedness of the network defined by arctic-alpine habitat patches (nodes) and least cost paths (edges).

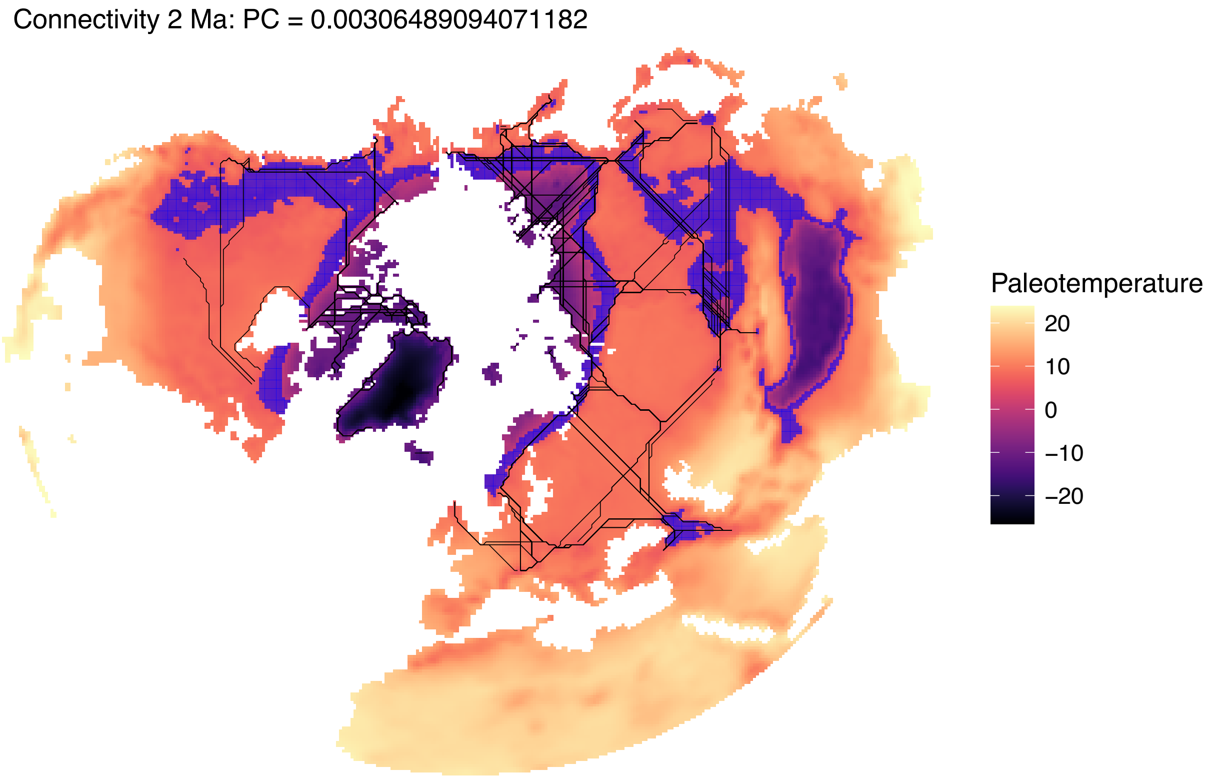

Figure S8: **Estimated distribution of arctic–alpine habitats and their connectivity across the Northern Hemisphere at 2 Ma.** Arctic-alpine habitats are shown in blue, and least cost paths connecting them are depicted as black lines. PC refers to probability of connectivity, which is a summary statistic of the global connectedness of the network defined by arctic-alpine habitat patches (nodes) and least cost paths (edges).

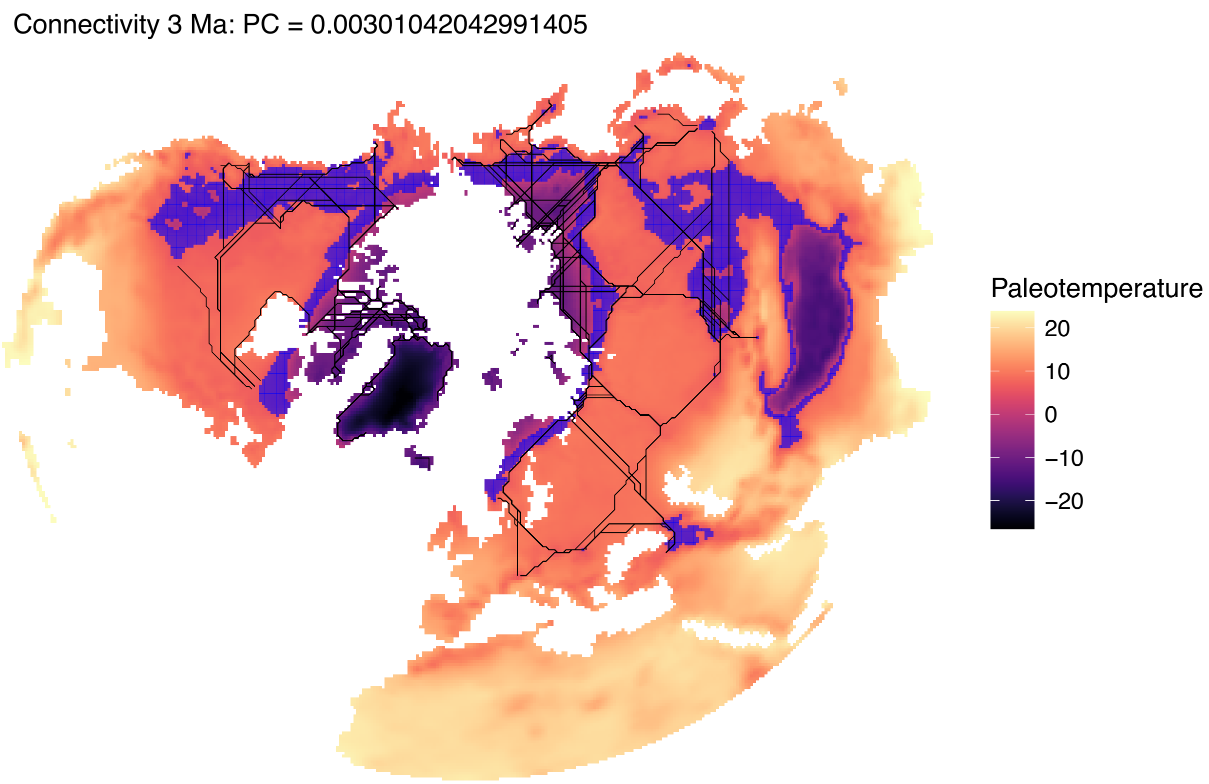

Figure S9: **Estimated distribution of arctic–alpine habitats and their connectivity across the Northern Hemisphere at 3 Ma.** Arctic-alpine habitats are shown in blue, and least cost paths connecting them are depicted as black lines. PC refers to probability of connectivity, which is a summary statistic of the global connectedness of the network defined by arctic-alpine habitat patches (nodes) and least cost paths (edges).

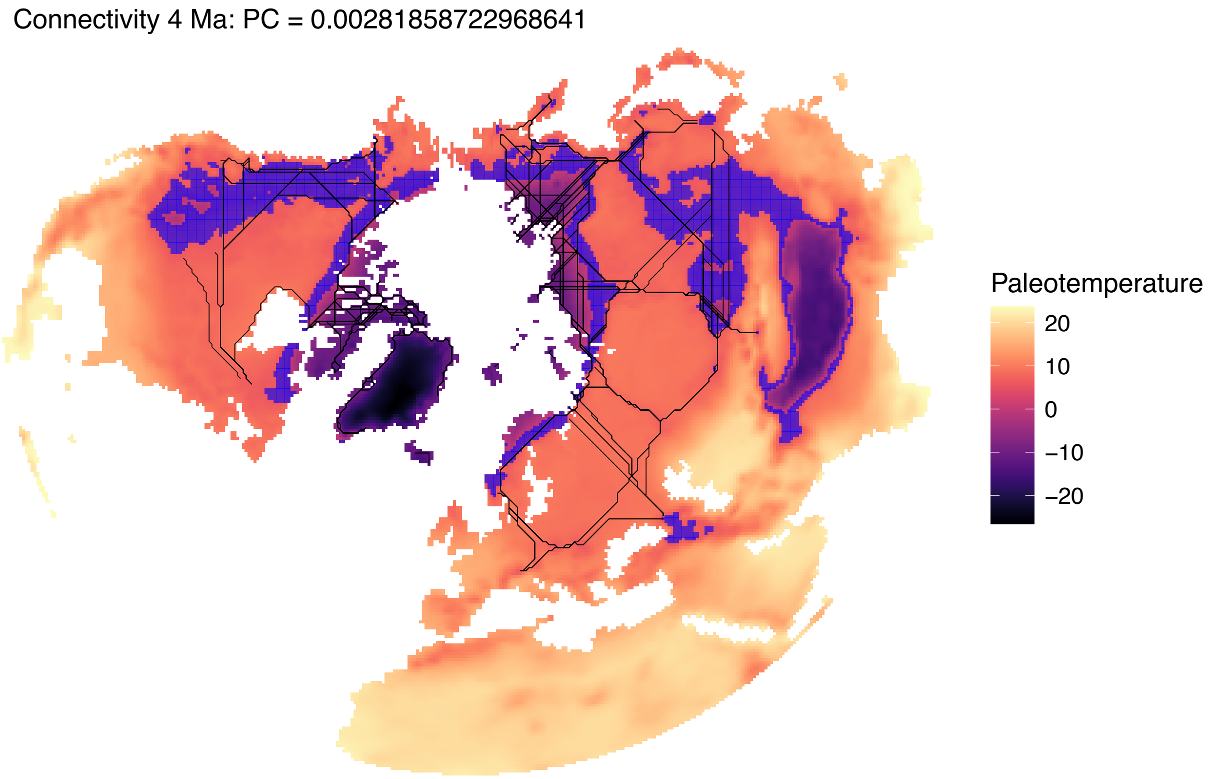

Figure S10: **Estimated distribution of arctic–alpine habitats and their connectivity across the Northern Hemisphere at 4 Ma.** Arctic-alpine habitats are shown in blue, and least cost paths connecting them are depicted as black lines. PC refers to probability of connectivity, which is a summary statistic of the global connectedness of the network defined by arctic-alpine habitat patches (nodes) and least cost paths (edges).

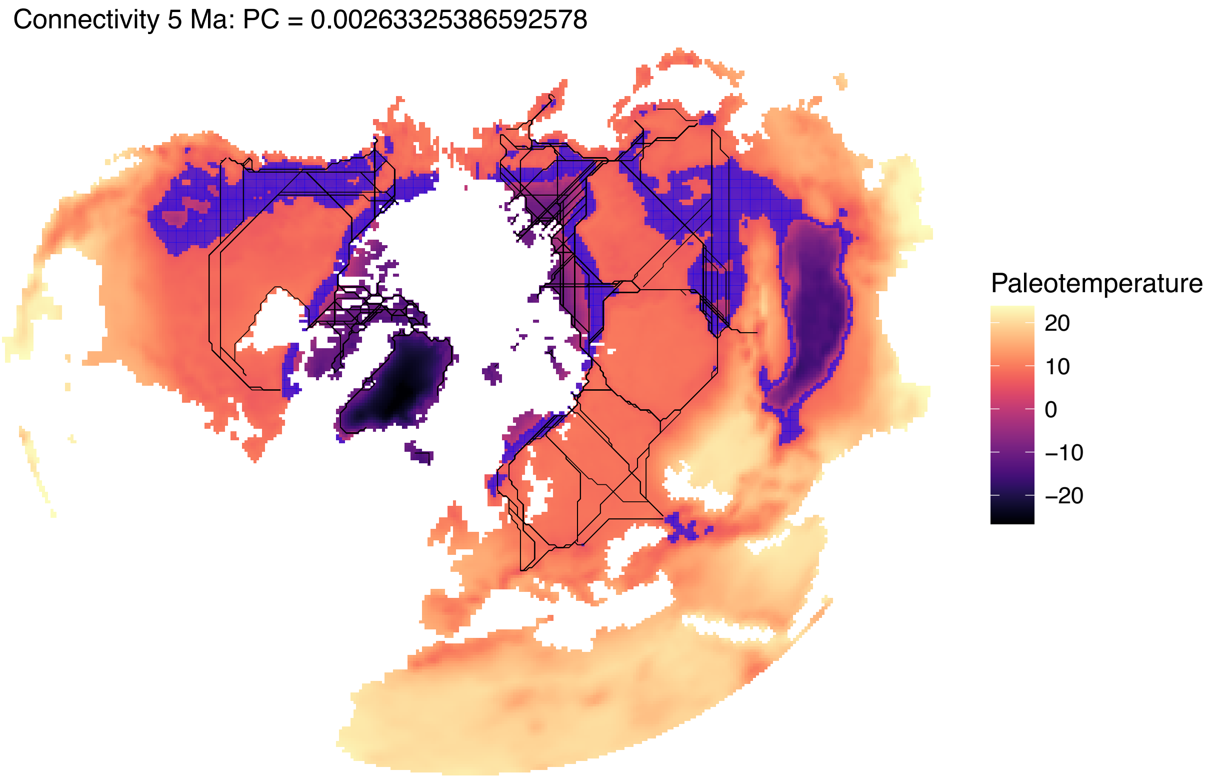

Figure S11: **Estimated distribution of arctic–alpine habitats and their connectivity across the Northern Hemisphere at 5 Ma.** Arctic-alpine habitats are shown in blue, and least cost paths connecting them are depicted as black lines. PC refers to probability of connectivity, which is a summary statistic of the global connectedness of the network defined by arctic-alpine habitat patches (nodes) and least cost paths (edges).

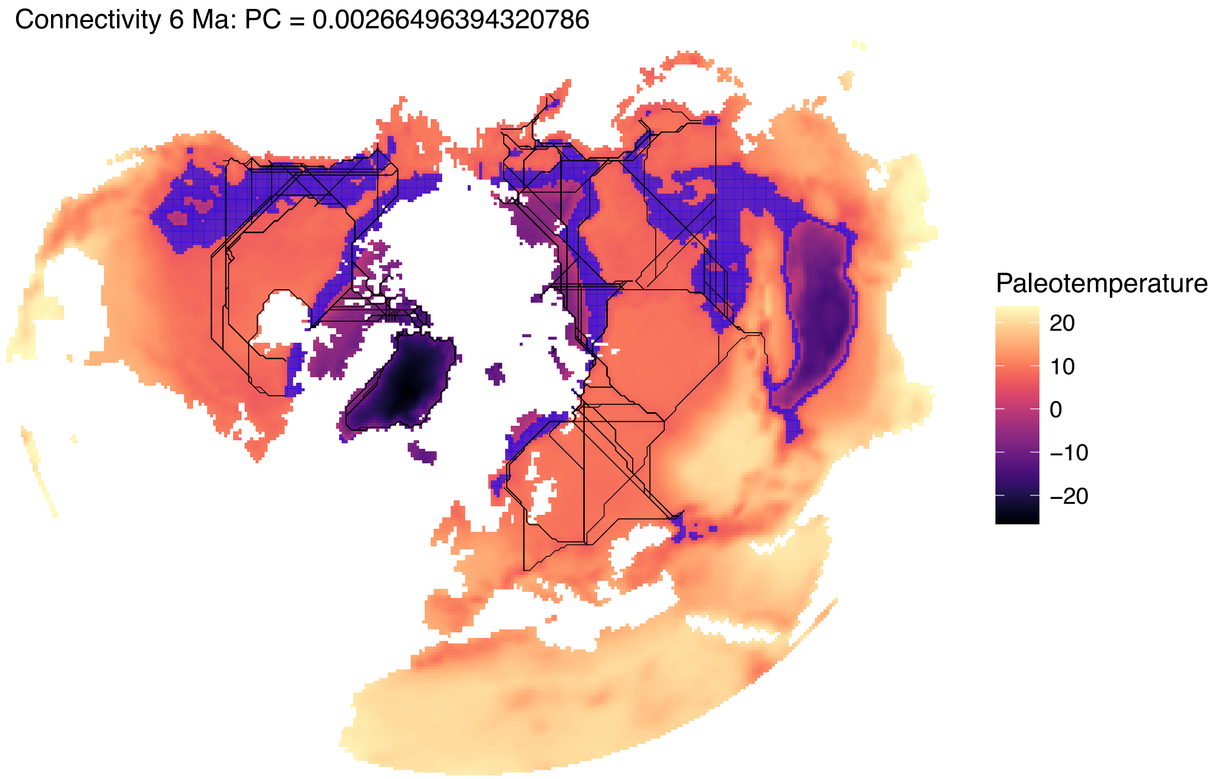

Figure S12: **Estimated distribution of arctic–alpine habitats and their connectivity across the Northern Hemisphere at 6 Ma.** Arctic-alpine habitats are shown in blue, and least cost paths connecting them are depicted as black lines. PC refers to probability of connectivity, which is a summary statistic of the global connectedness of the network defined by arctic-alpine habitat patches (nodes) and least cost paths (edges).

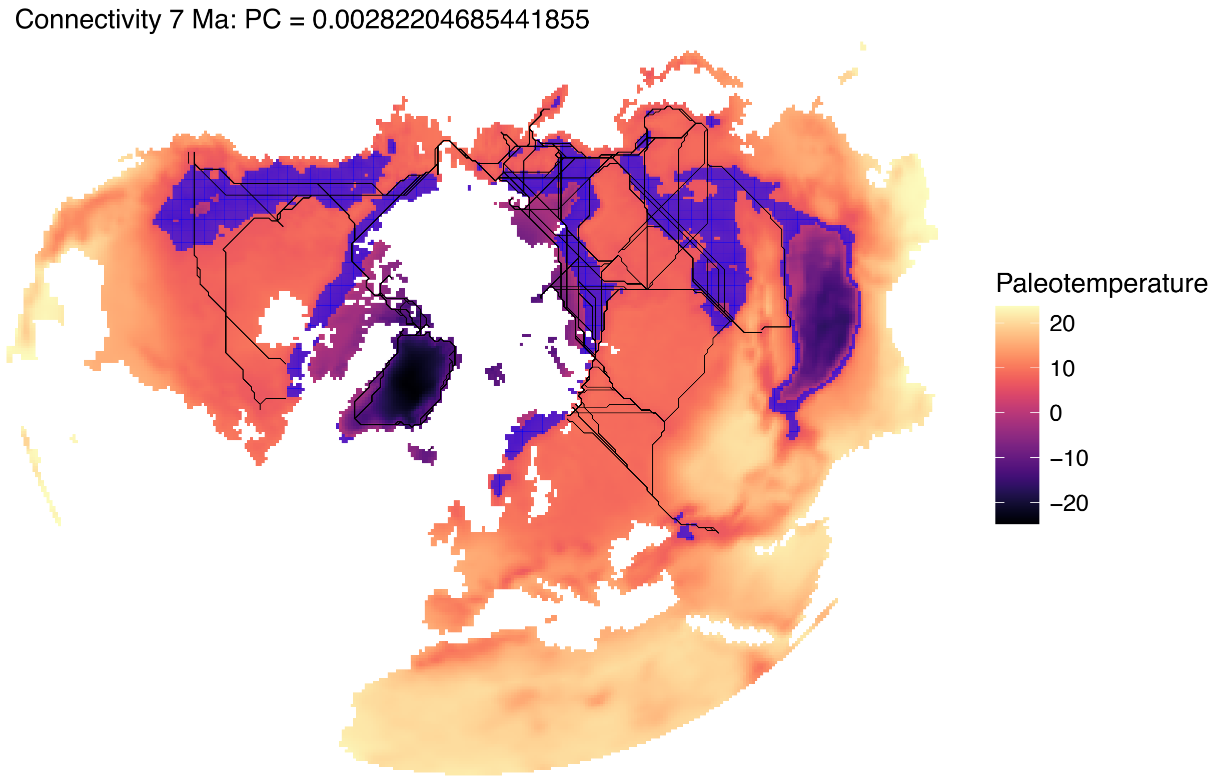

Figure S13: **Estimated distribution of arctic–alpine habitats and their connectivity across the Northern Hemisphere at 7 Ma.** Arctic-alpine habitats are shown in blue, and least cost paths connecting them are depicted as black lines. PC refers to probability of connectivity, which is a summary statistic of the global connectedness of the network defined by arctic-alpine habitat patches (nodes) and least cost paths (edges).

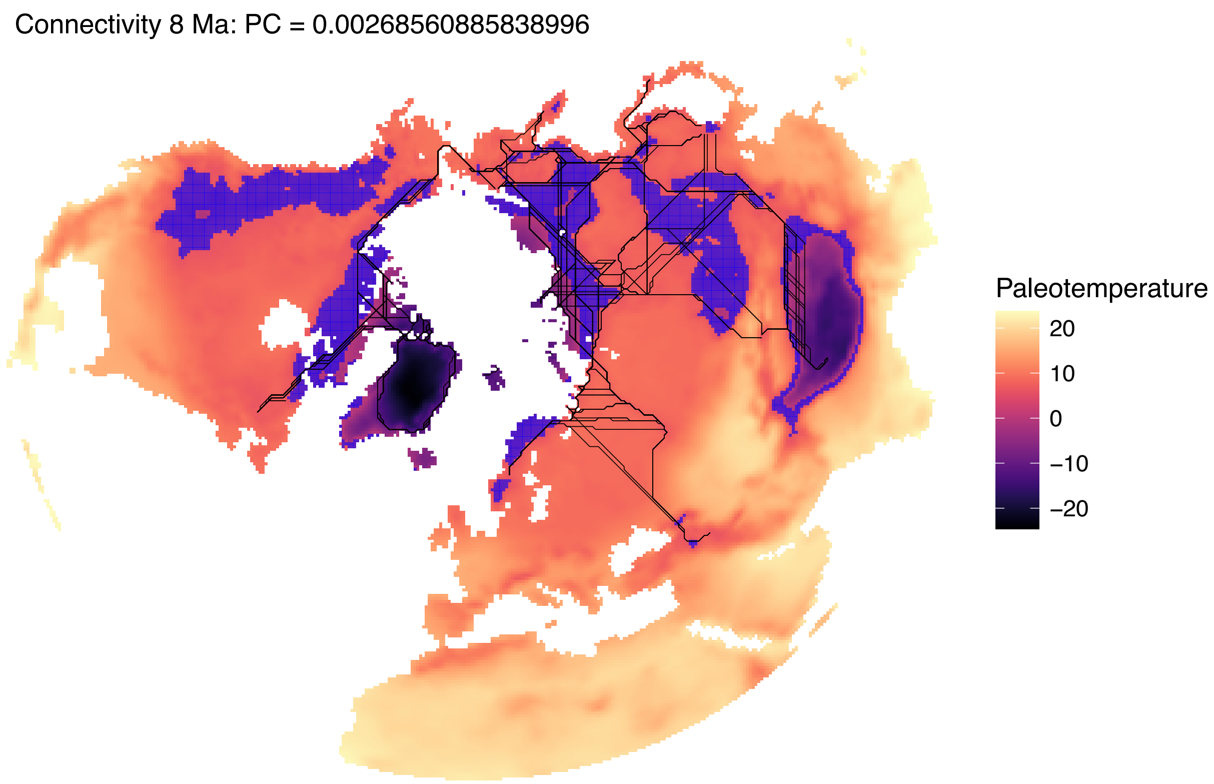

Figure S14: **Estimated distribution of arctic–alpine habitats and their connectivity across the Northern Hemisphere at 8 Ma.** Arctic-alpine habitats are shown in blue, and least cost paths connecting them are depicted as black lines. PC refers to probability of connectivity, which is a summary statistic of the global connectedness of the network defined by arctic-alpine habitat patches (nodes) and least cost paths (edges).

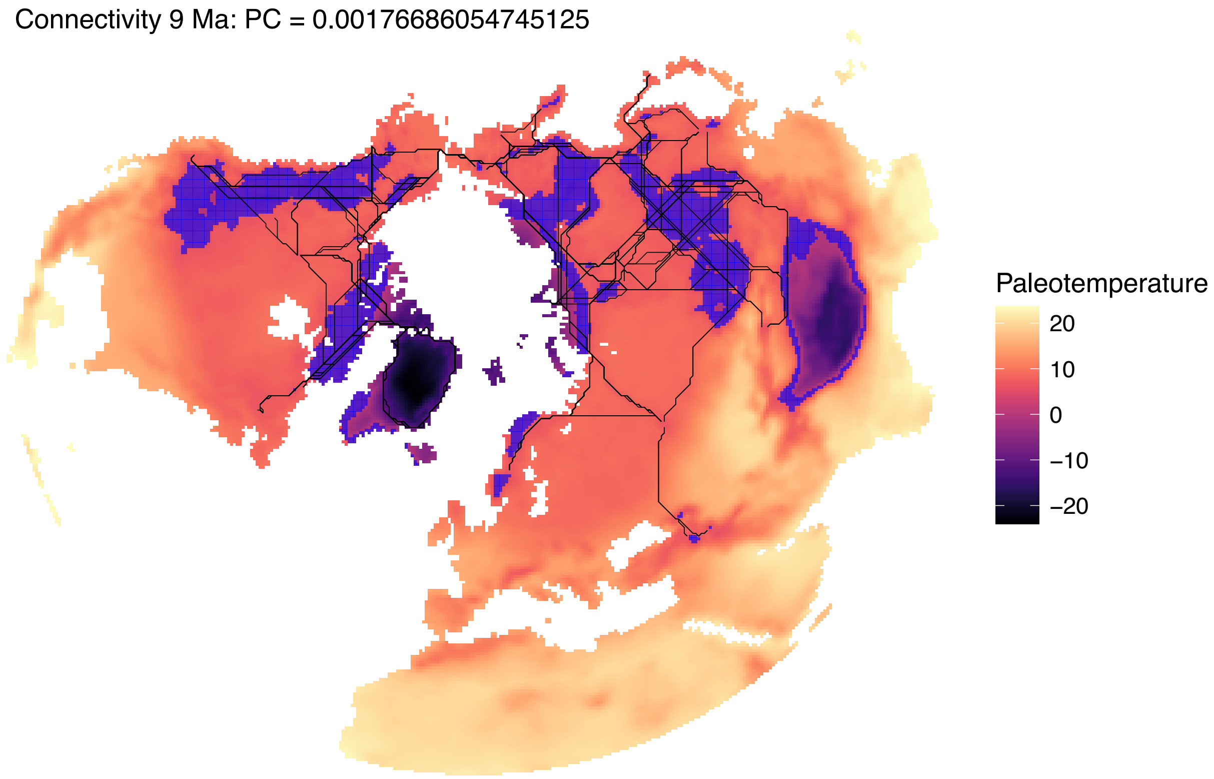

Figure S15: **Estimated distribution of arctic–alpine habitats and their connectivity across the Northern Hemisphere at 9 Ma.** Arctic-alpine habitats are shown in blue, and least cost paths connecting them are depicted as black lines. PC refers to probability of connectivity, which is a summary statistic of the global connectedness of the network defined by arctic-alpine habitat patches (nodes) and least cost paths (edges).

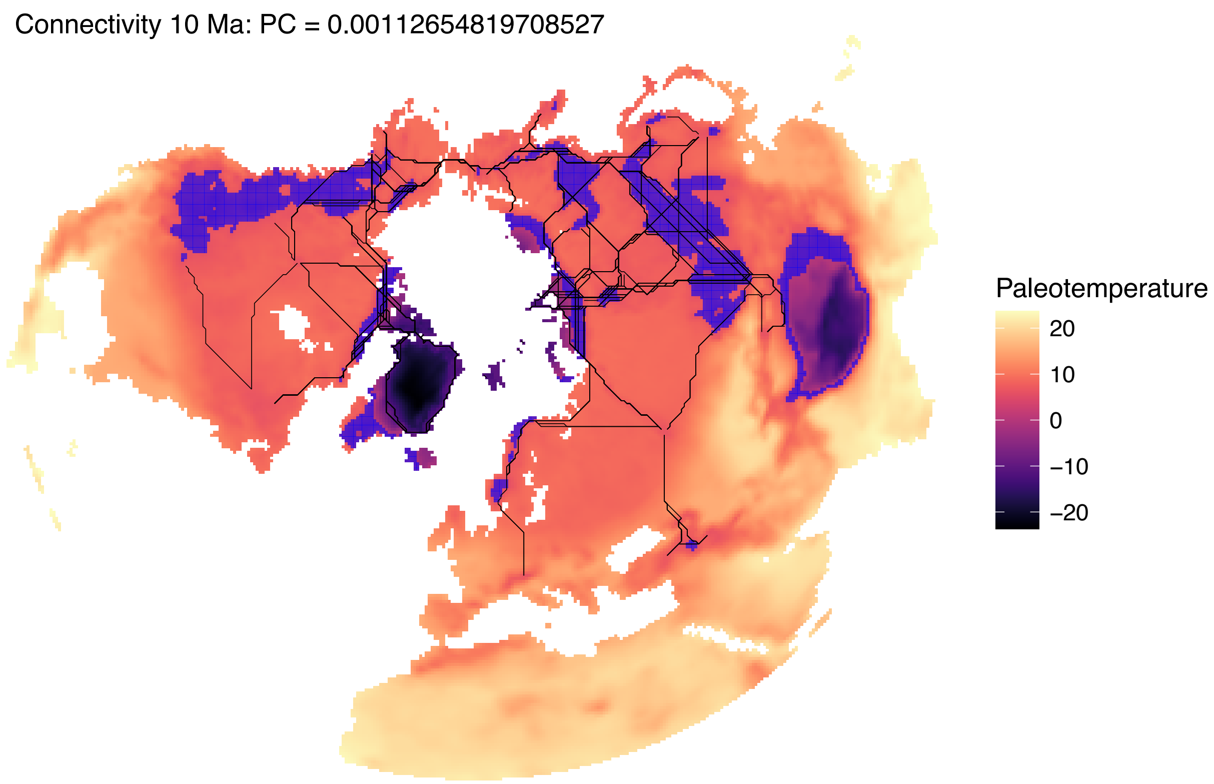

Figure S16: **Estimated distribution of arctic–alpine habitats and their connectivity across the Northern Hemisphere at 10 Ma.** Arctic-alpine habitats are shown in blue, and least cost paths connecting them are depicted as black lines. PC refers to probability of connectivity, which is a summary statistic of the global connectedness of the network defined by arctic-alpine habitat patches (nodes) and least cost paths (edges).

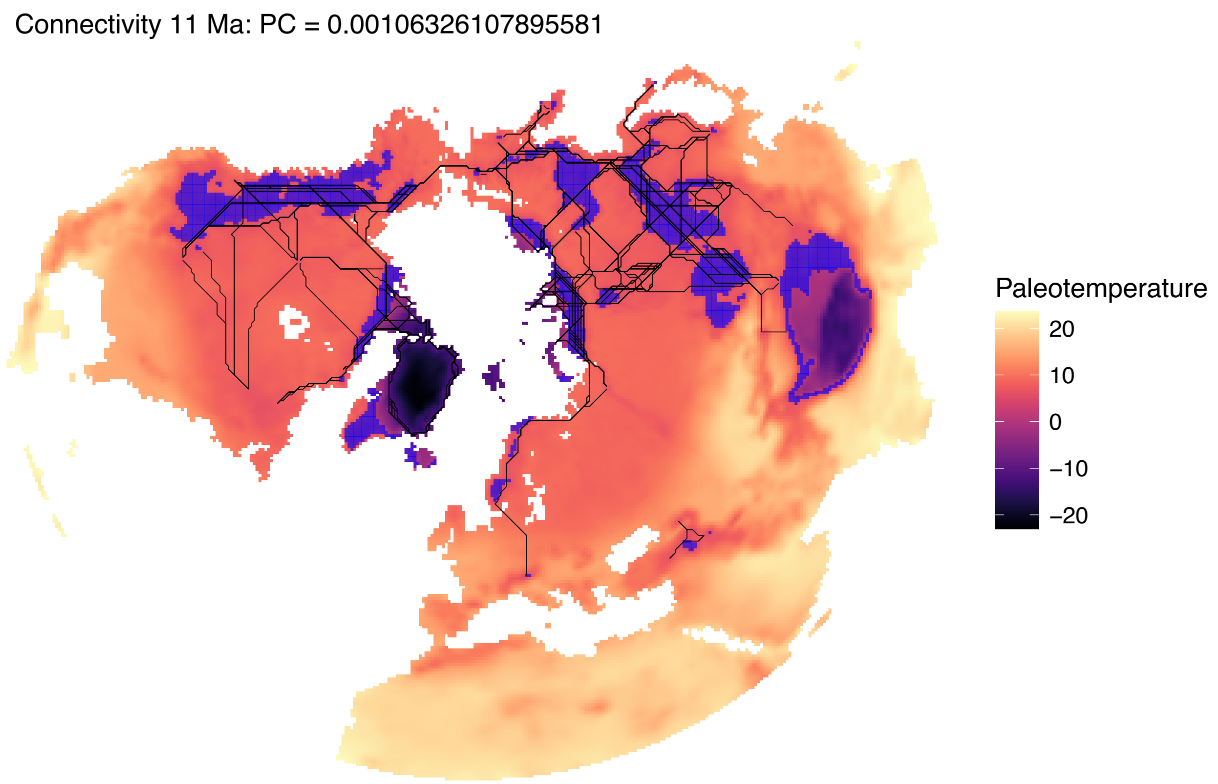

Figure S17: **Estimated distribution of arctic–alpine habitats and their connectivity across the Northern Hemisphere at 11 Ma.** Arctic-alpine habitats are shown in blue, and least cost paths connecting them are depicted as black lines. PC refers to probability of connectivity, which is a summary statistic of the global connectedness of the network defined by arctic-alpine habitat patches (nodes) and least cost paths (edges).

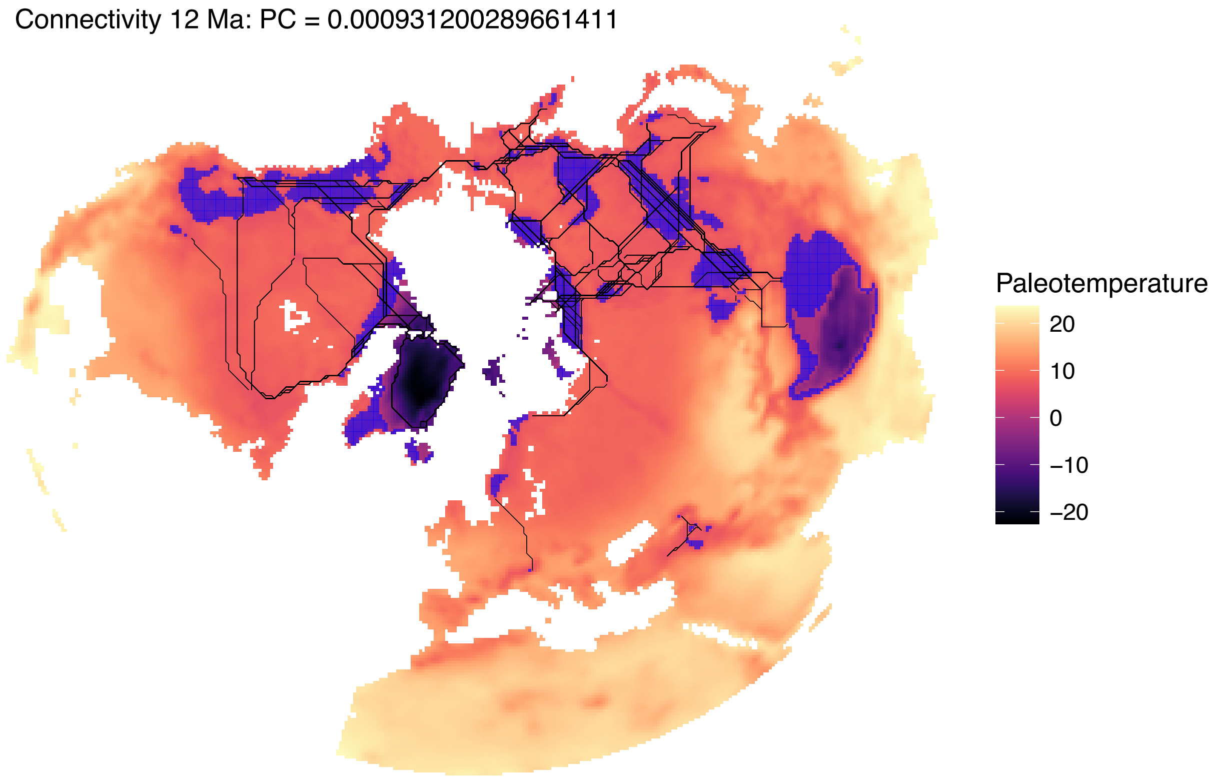

Figure S18: **Estimated distribution of arctic–alpine habitats and their connectivity across the Northern Hemisphere at 12 Ma.** Arctic-alpine habitats are shown in blue, and least cost paths connecting them are depicted as black lines. PC refers to probability of connectivity, which is a summary statistic of the global connectedness of the network defined by arctic-alpine habitat patches (nodes) and least cost paths (edges).

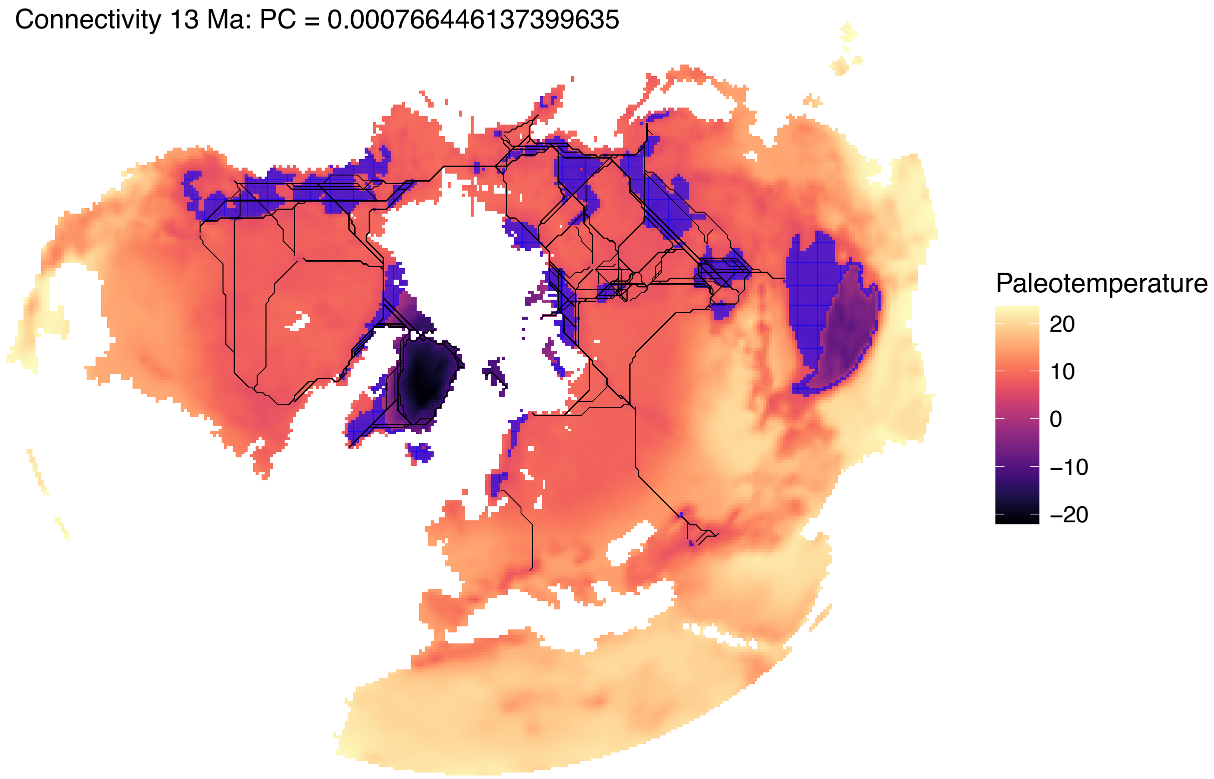

Figure S19: **Estimated distribution of arctic–alpine habitats and their connectivity across the Northern Hemisphere at 13 Ma.** Arctic-alpine habitats are shown in blue, and least cost paths connecting them are depicted as black lines. PC refers to probability of connectivity, which is a summary statistic of the global connectedness of the network defined by arctic-alpine habitat patches (nodes) and least cost paths (edges).

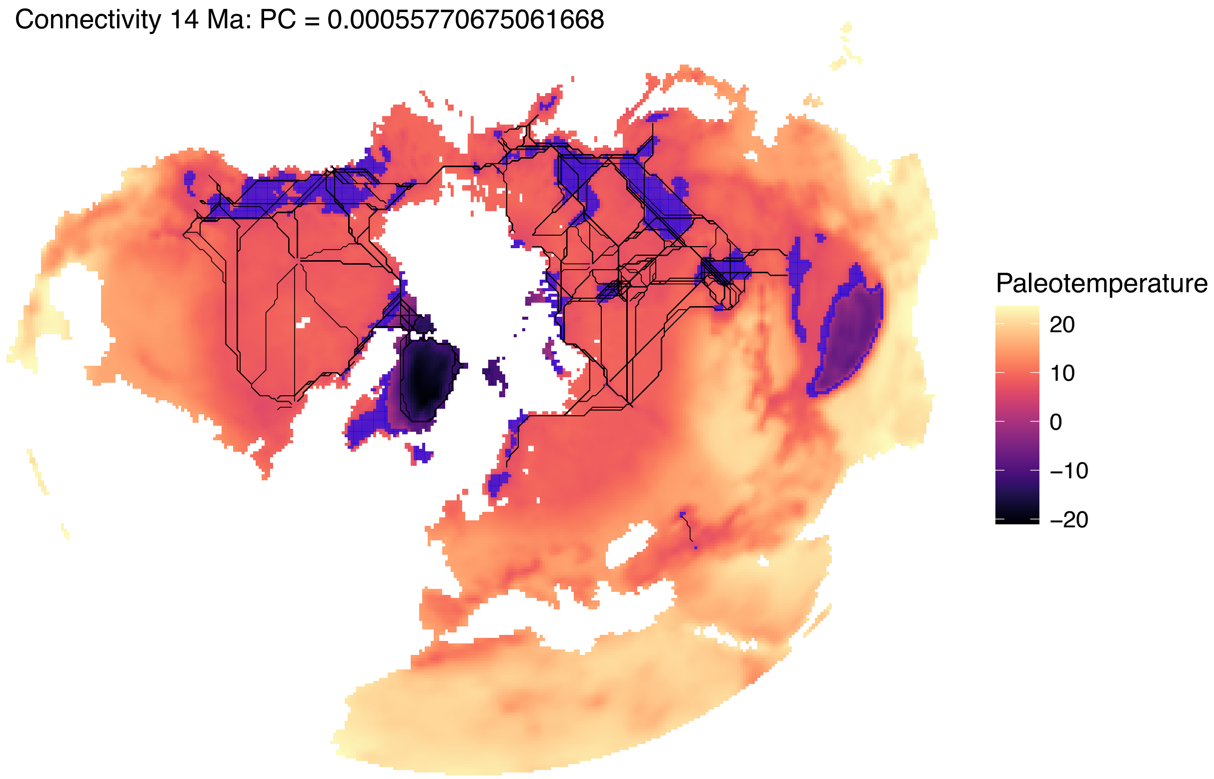

Figure S20: **Estimated distribution of arctic–alpine habitats and their connectivity across the Northern Hemisphere at 14 Ma.** Arctic-alpine habitats are shown in blue, and least cost paths connecting them are depicted as black lines. PC refers to probability of connectivity, which is a summary statistic of the global connectedness of the network defined by arctic-alpine habitat patches (nodes) and least cost paths (edges).

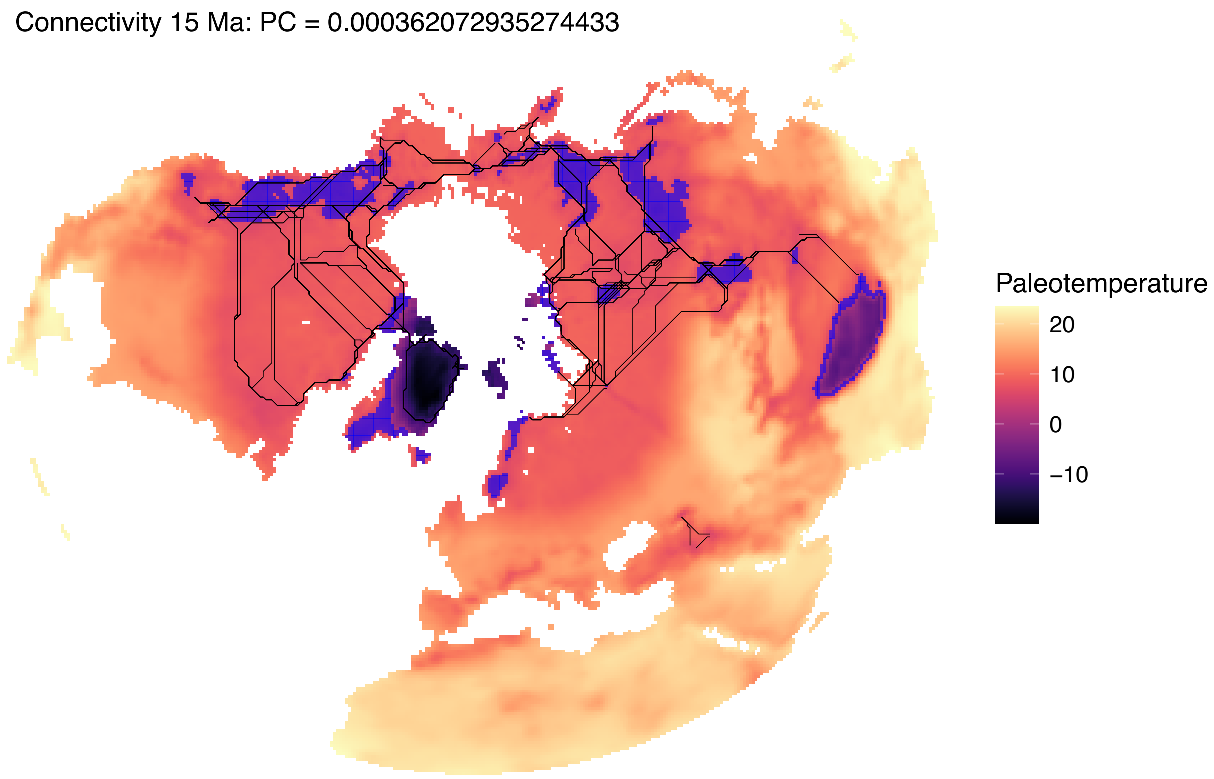

Figure S21: **Estimated distribution of arctic–alpine habitats and their connectivity across the Northern Hemisphere at 15 Ma.** Arctic-alpine habitats are shown in blue, and least cost paths connecting them are depicted as black lines. PC refers to probability of connectivity, which is a summary statistic of the global connectedness of the network defined by arctic-alpine habitat patches (nodes) and least cost paths (edges).

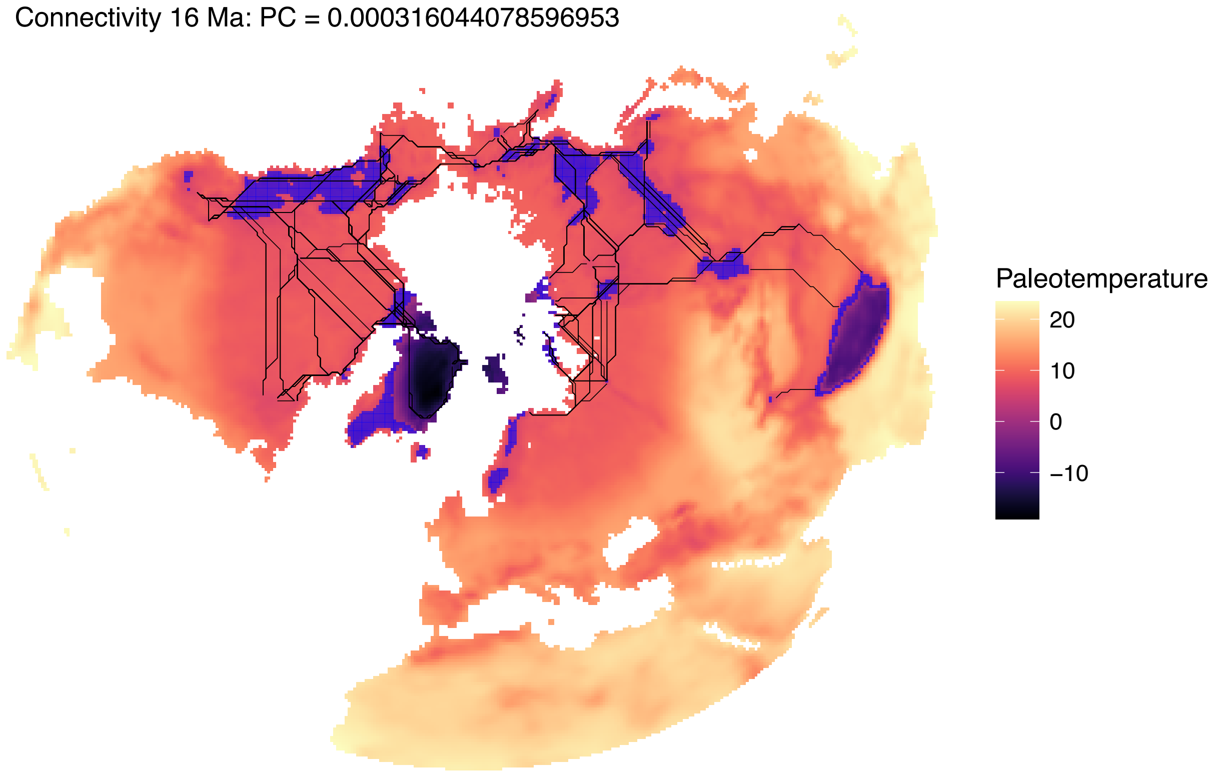

Figure S22: **Estimated distribution of arctic–alpine habitats and their connectivity across the Northern Hemisphere at 16 Ma.** Arctic-alpine habitats are shown in blue, and least cost paths connecting them are depicted as black lines. PC refers to probability of connectivity, which is a summary statistic of the global connectedness of the network defined by arctic-alpine habitat patches (nodes) and least cost paths (edges).

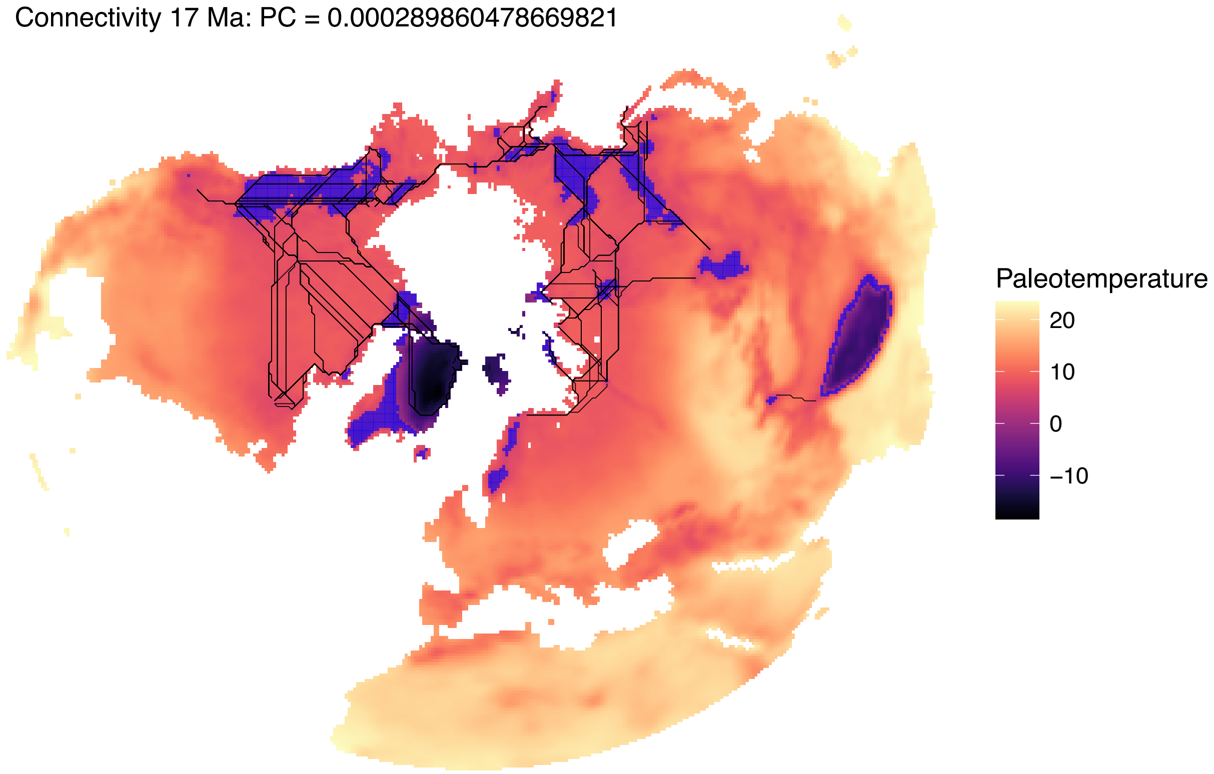

Figure S23: **Estimated distribution of arctic–alpine habitats and their connectivity across the Northern Hemisphere at 17 Ma.** Arctic-alpine habitats are shown in blue, and least cost paths connecting them are depicted as black lines. PC refers to probability of connectivity, which is a summary statistic of the global connectedness of the network defined by arctic-alpine habitat patches (nodes) and least cost paths (edges).

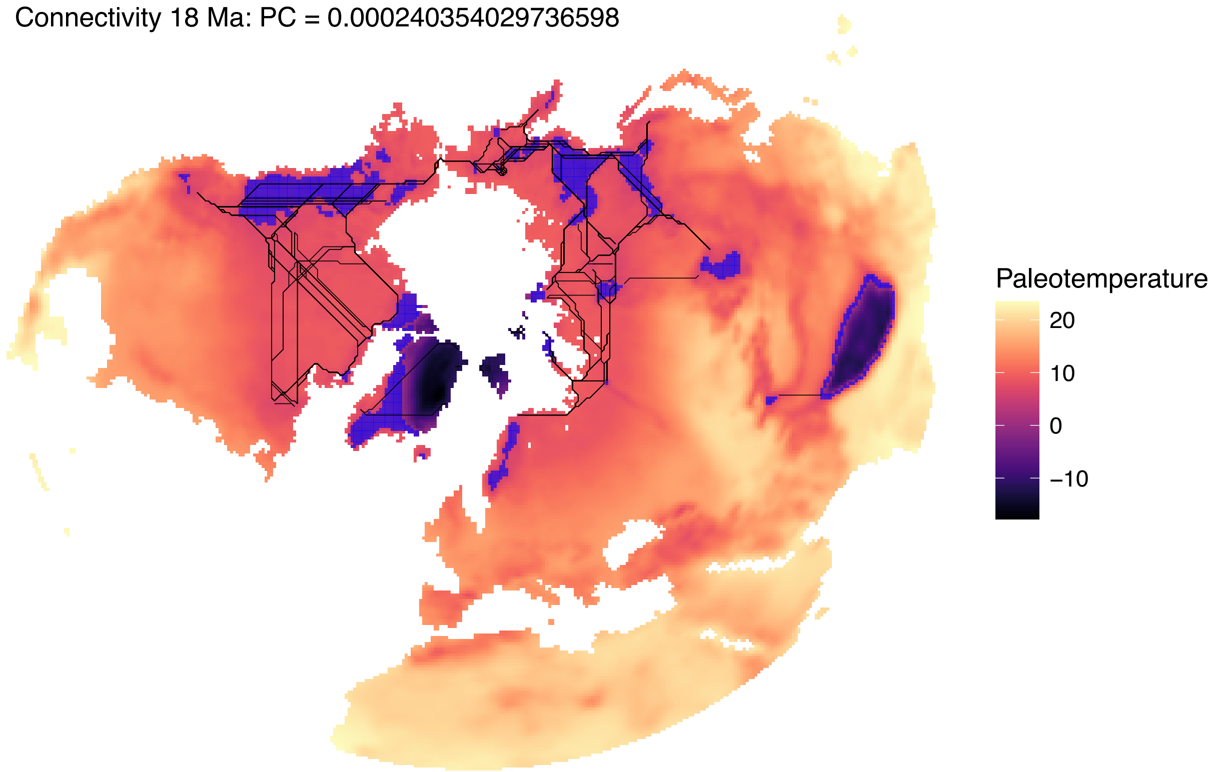

Figure S24: **Estimated distribution of arctic–alpine habitats and their connectivity across the Northern Hemisphere at 18 Ma.** Arctic-alpine habitats are shown in blue, and least cost paths connecting them are depicted as black lines. PC refers to probability of connectivity, which is a summary statistic of the global connectedness of the network defined by arctic-alpine habitat patches (nodes) and least cost paths (edges).

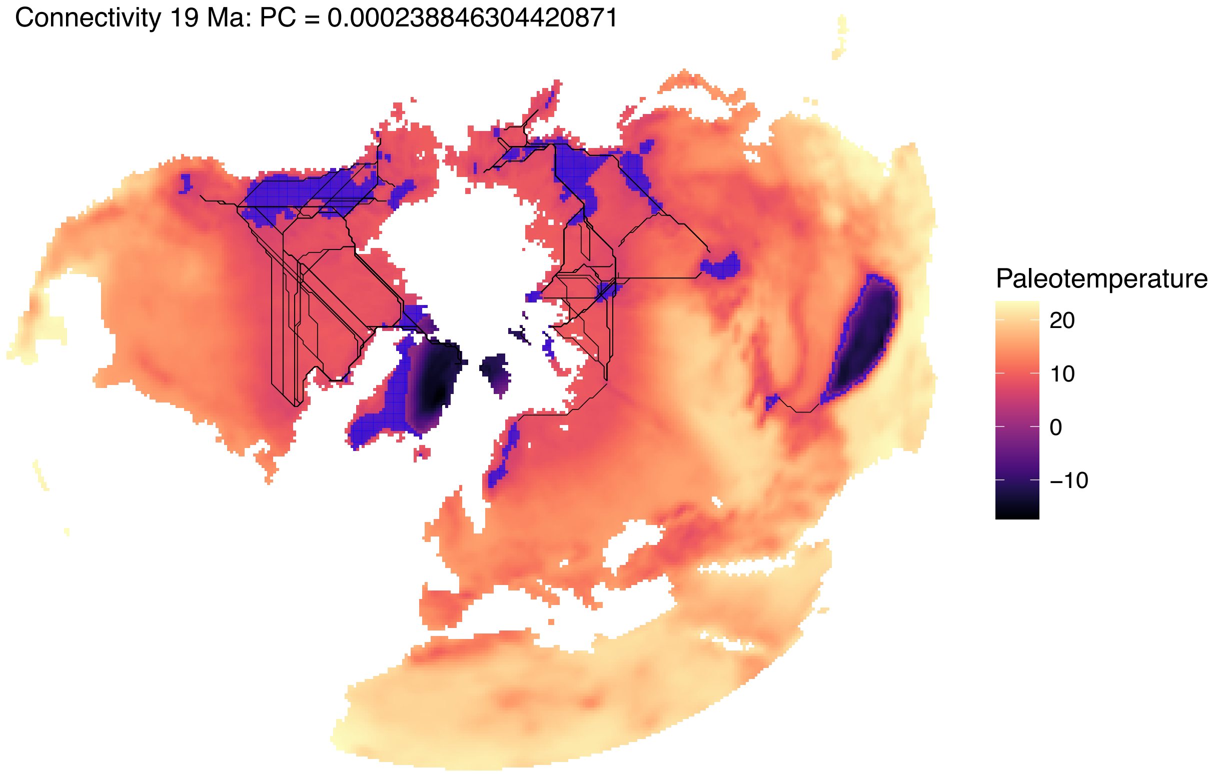

Figure S25: **Estimated distribution of arctic–alpine habitats and their connectivity across the Northern Hemisphere at 19 Ma.** Arctic-alpine habitats are shown in blue, and least cost paths connecting them are depicted as black lines. PC refers to probability of connectivity, which is a summary statistic of the global connectedness of the network defined by arctic-alpine habitat patches (nodes) and least cost paths (edges).

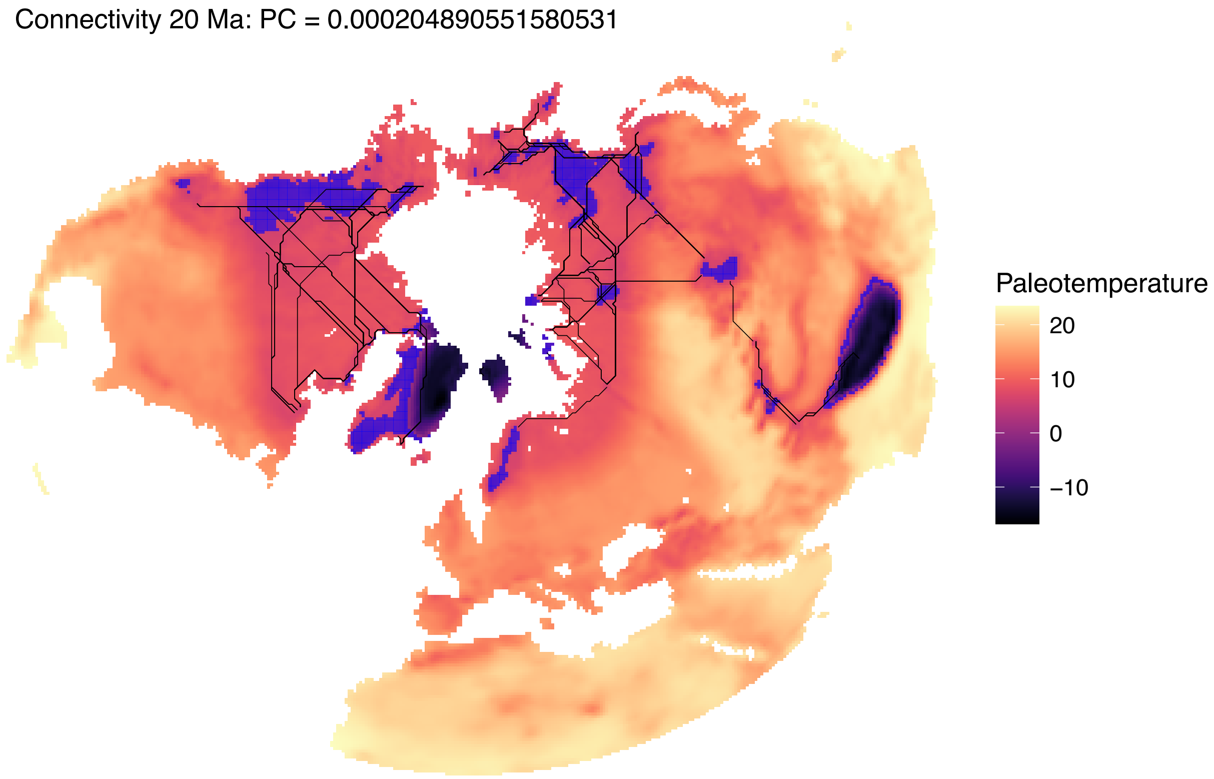

Figure S26: **Estimated distribution of arctic–alpine habitats and their connectivity across the Northern Hemisphere at 20 Ma.** Arctic-alpine habitats are shown in blue, and least cost paths connecting them are depicted as black lines. PC refers to probability of connectivity, which is a summary statistic of the global connectedness of the network defined by arctic-alpine habitat patches (nodes) and least cost paths (edges).

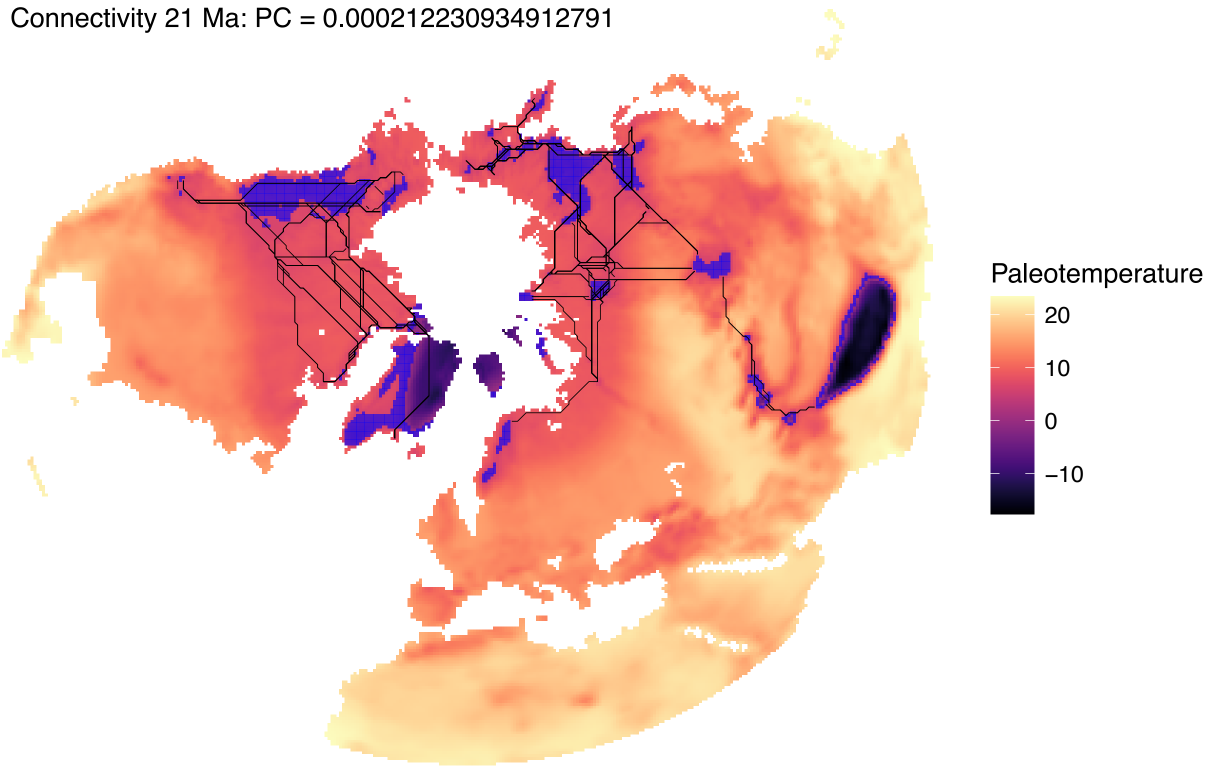

Figure S27: **Estimated distribution of arctic–alpine habitats and their connectivity across the Northern Hemisphere at 21 Ma.** Arctic-alpine habitats are shown in blue, and least cost paths connecting them are depicted as black lines. PC refers to probability of connectivity, which is a summary statistic of the global connectedness of the network defined by arctic-alpine habitat patches (nodes) and least cost paths (edges).

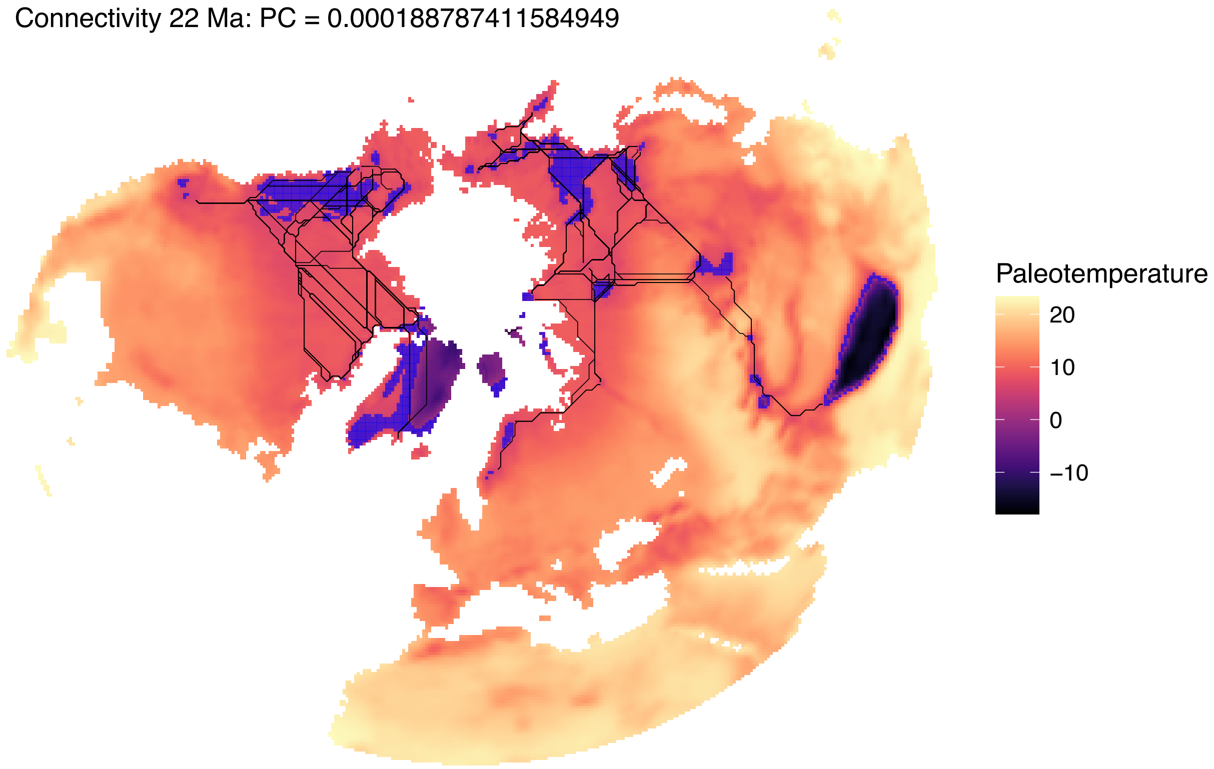

Figure S28: **Estimated distribution of arctic–alpine habitats and their connectivity across the Northern Hemisphere at 22 Ma.** Arctic-alpine habitats are shown in blue, and least cost paths connecting them are depicted as black lines. PC refers to probability of connectivity, which is a summary statistic of the global connectedness of the network defined by arctic-alpine habitat patches (nodes) and least cost paths (edges).

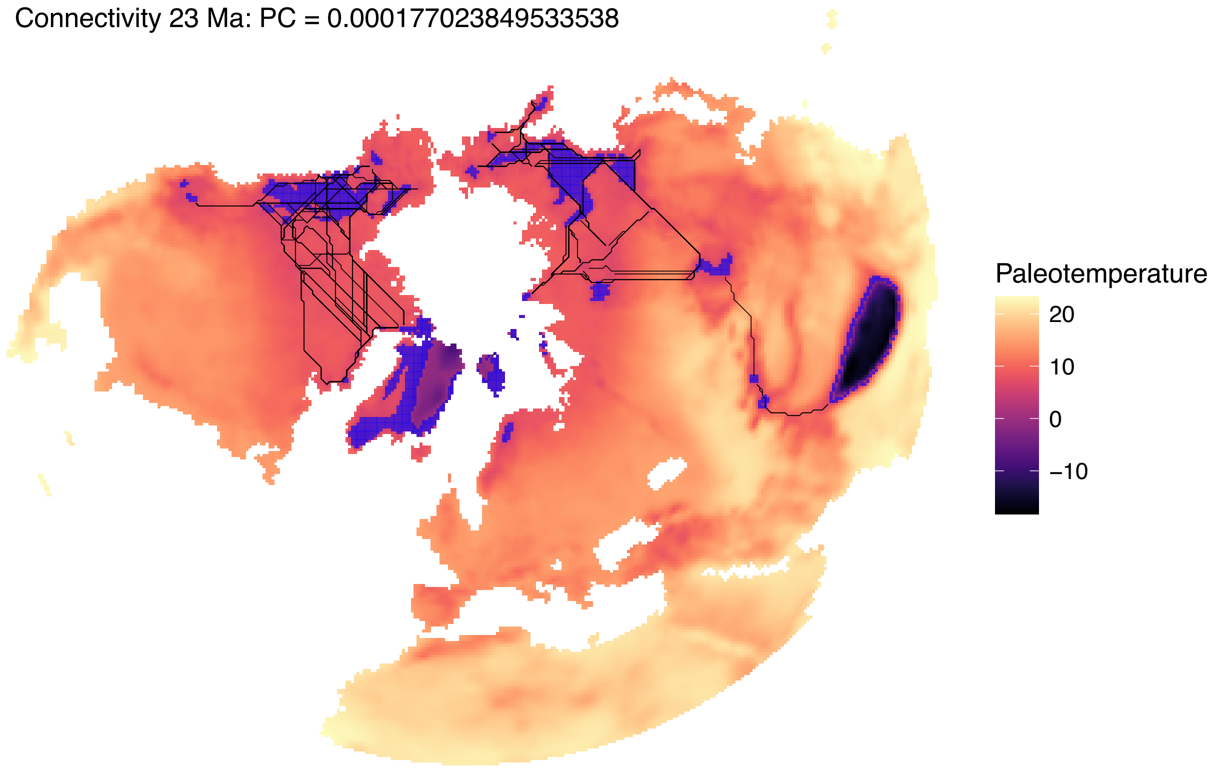

Figure S29: **Estimated distribution of arctic–alpine habitats and their connectivity across the Northern Hemisphere at 23 Ma.** Arctic-alpine habitats are shown in blue, and least cost paths connecting them are depicted as black lines. PC refers to probability of connectivity, which is a summary statistic of the global connectedness of the network defined by arctic-alpine habitat patches (nodes) and least cost paths (edges).

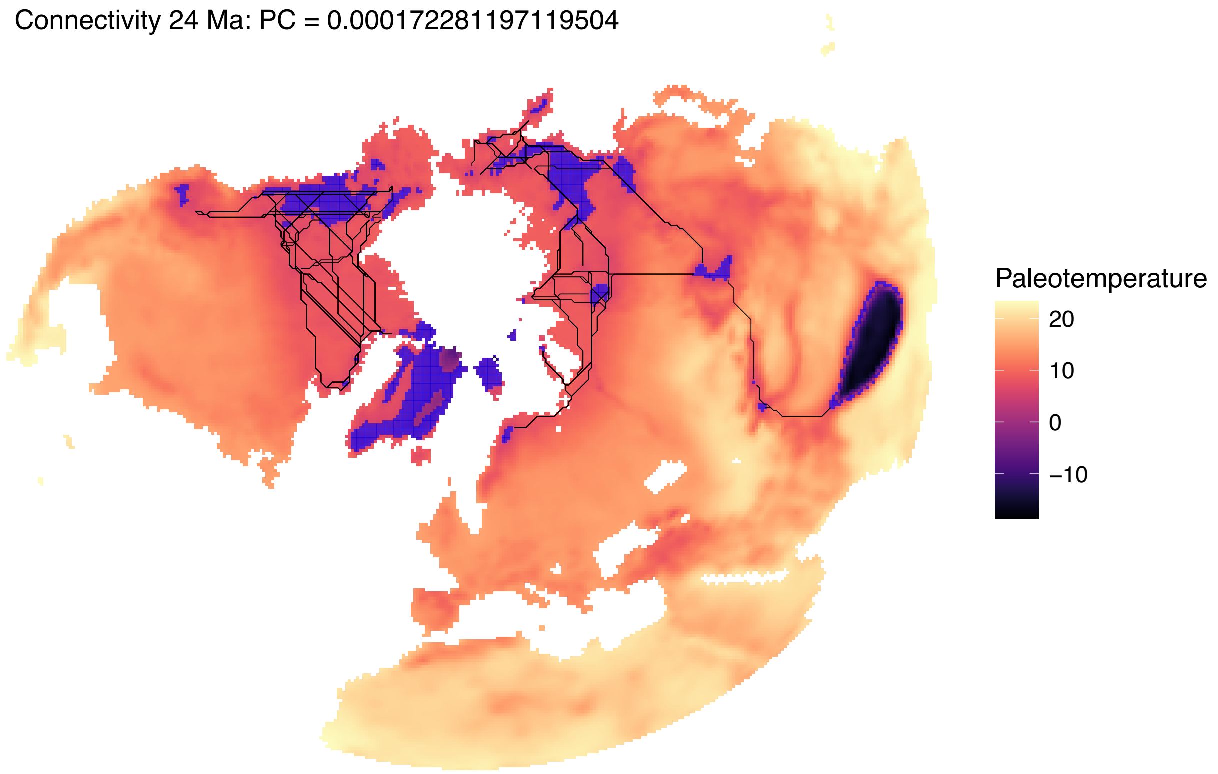

Figure S30: **Estimated distribution of arctic–alpine habitats and their connectivity across the Northern Hemisphere at 24 Ma.** Arctic-alpine habitats are shown in blue, and least cost paths connecting them are depicted as black lines. PC refers to probability of connectivity, which is a summary statistic of the global connectedness of the network defined by arctic-alpine habitat patches (nodes) and least cost paths (edges).

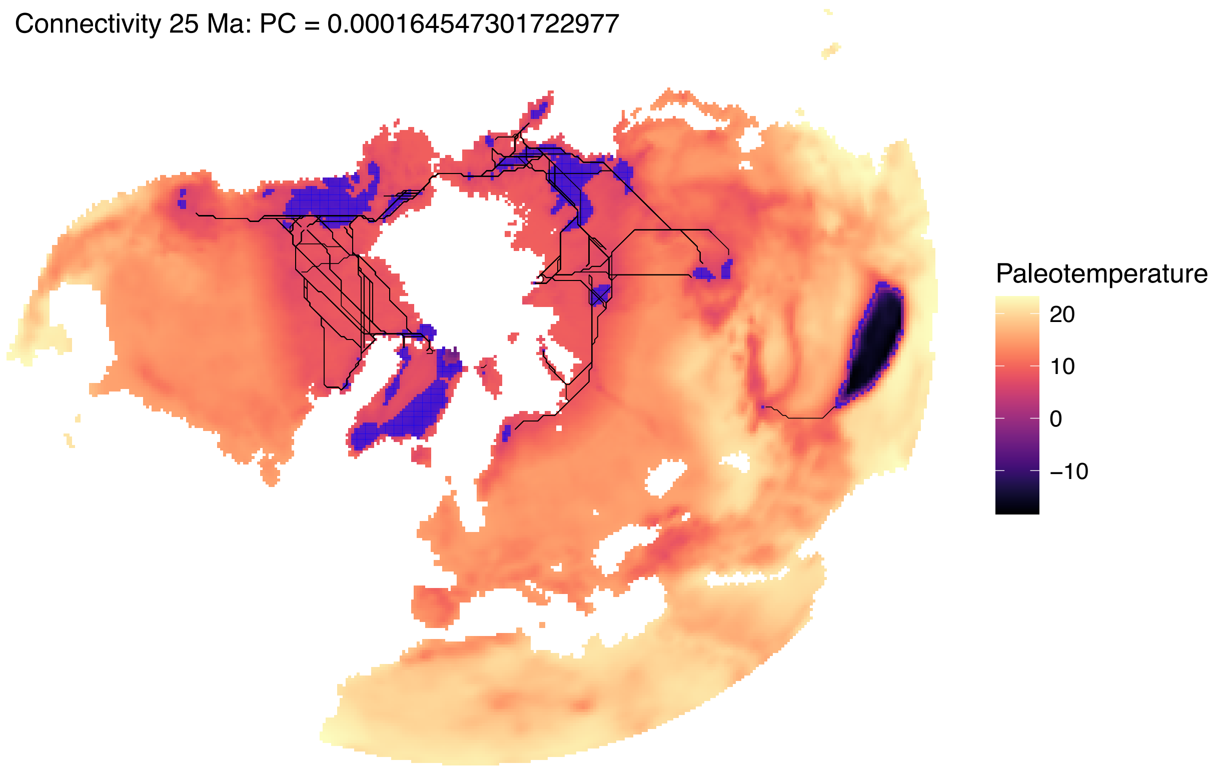

Figure S31: **Estimated distribution of arctic–alpine habitats and their connectivity across the Northern Hemisphere at 25 Ma.** Arctic-alpine habitats are shown in blue, and least cost paths connecting them are depicted as black lines. PC refers to probability of connectivity, which is a summary statistic of the global connectedness of the network defined by arctic-alpine habitat patches (nodes) and least cost paths (edges).

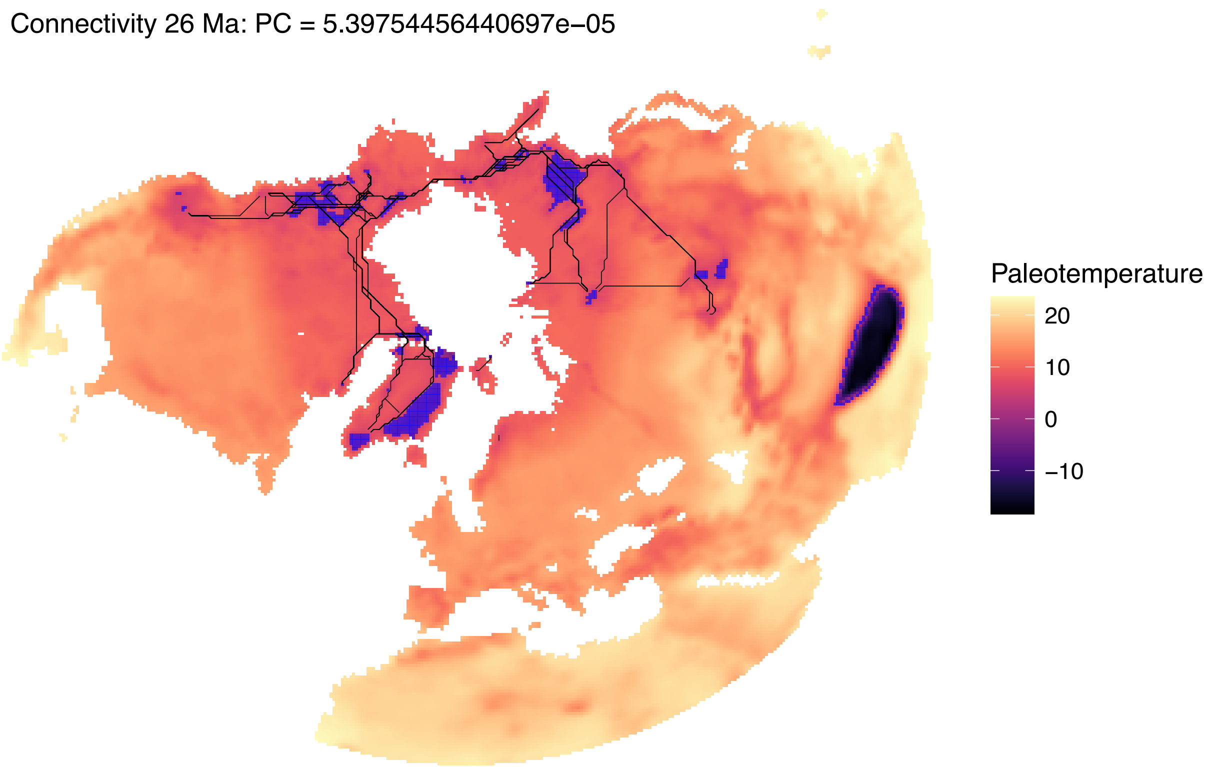

Figure S32: **Estimated distribution of arctic–alpine habitats and their connectivity across the Northern Hemisphere at 26 Ma.** Arctic-alpine habitats are shown in blue, and least cost paths connecting them are depicted as black lines. PC refers to probability of connectivity, which is a summary statistic of the global connectedness of the network defined by arctic-alpine habitat patches (nodes) and least cost paths (edges).

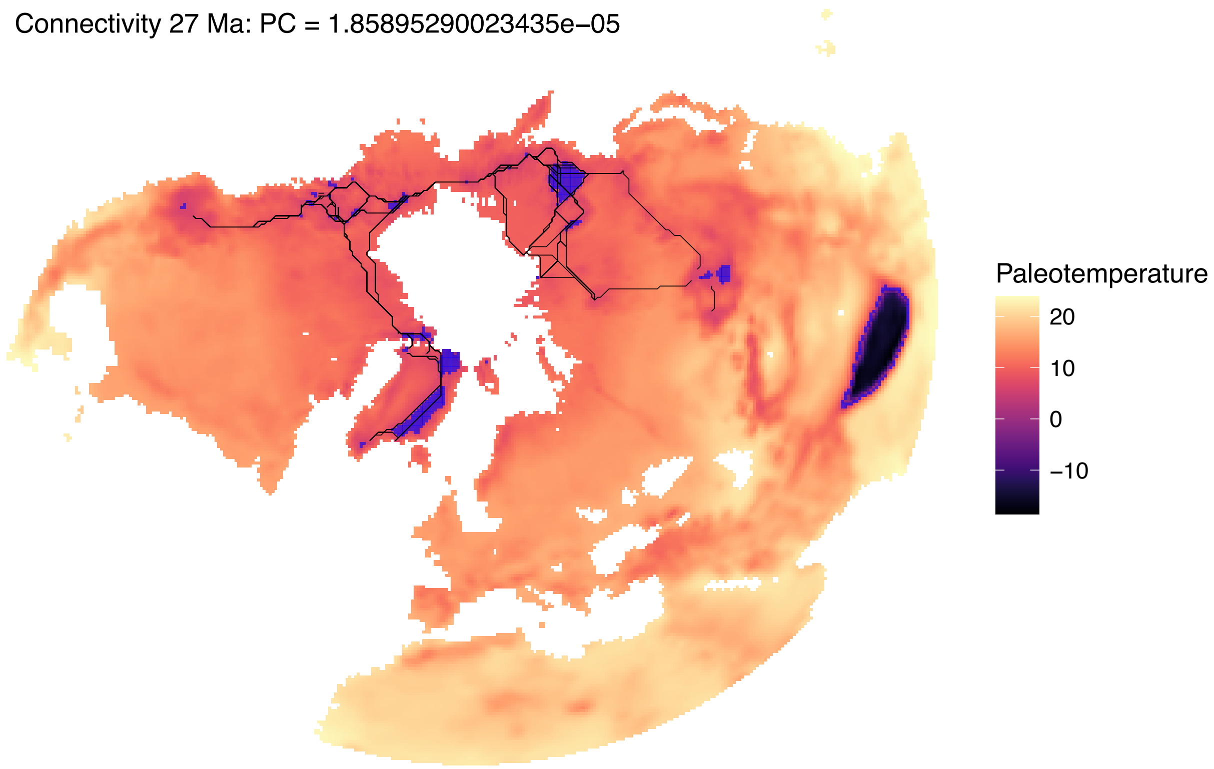

Figure S33: **Estimated distribution of arctic–alpine habitats and their connectivity across the Northern Hemisphere at 27 Ma.** Arctic-alpine habitats are shown in blue, and least cost paths connecting them are depicted as black lines. PC refers to probability of connectivity, which is a summary statistic of the global connectedness of the network defined by arctic-alpine habitat patches (nodes) and least cost paths (edges).

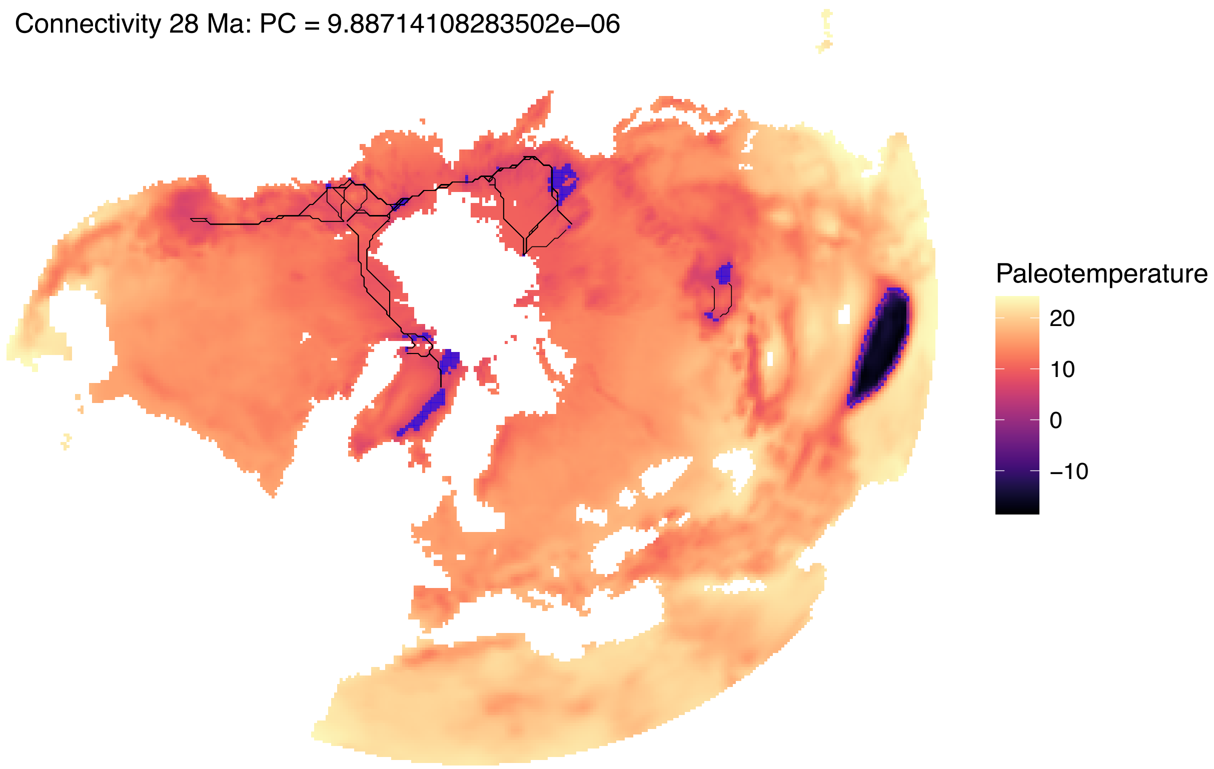

Figure S34: **Estimated distribution of arctic–alpine habitats and their connectivity across the Northern Hemisphere at 28 Ma.** Arctic-alpine habitats are shown in blue, and least cost paths connecting them are depicted as black lines. PC refers to probability of connectivity, which is a summary statistic of the global connectedness of the network defined by arctic-alpine habitat patches (nodes) and least cost paths (edges).

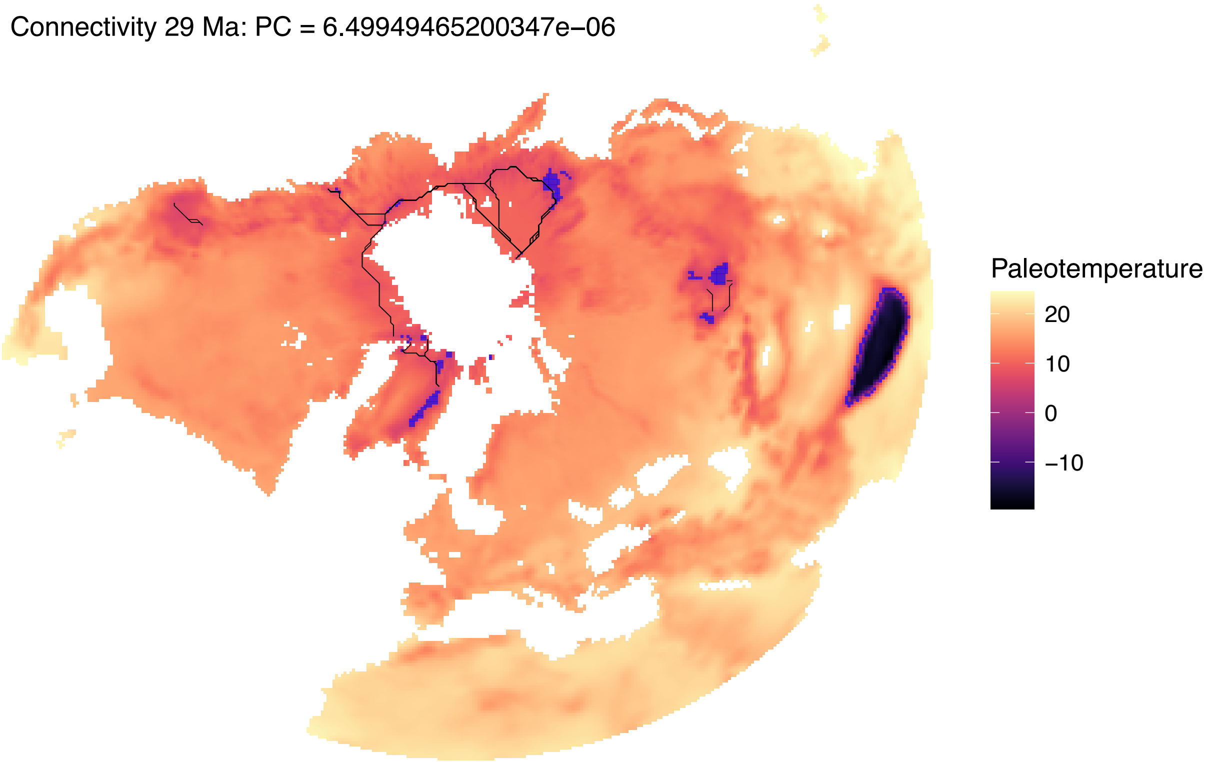

Figure S35: **Estimated distribution of arctic–alpine habitats and their connectivity across the Northern Hemisphere at 29 Ma.** Arctic-alpine habitats are shown in blue, and least cost paths connecting them are depicted as black lines. PC refers to probability of connectivity, which is a summary statistic of the global connectedness of the network defined by arctic-alpine habitat patches (nodes) and least cost paths (edges).

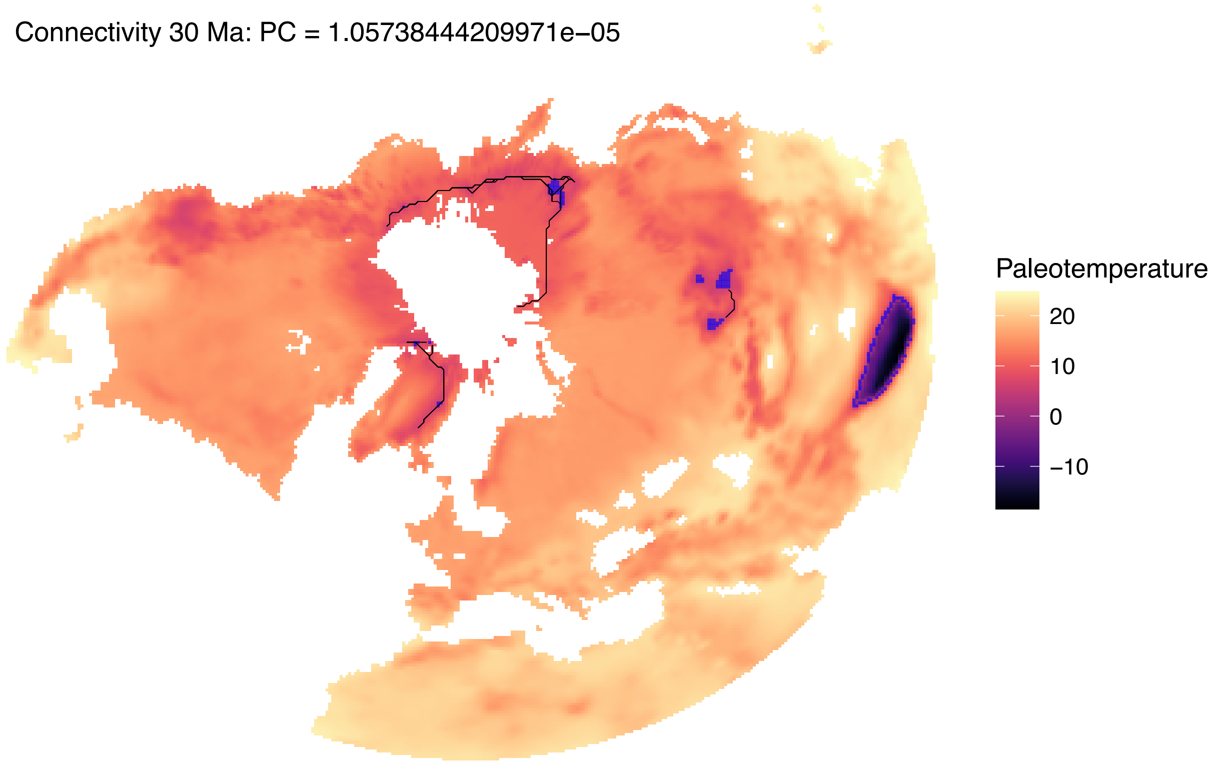

Figure S36: **Estimated distribution of arctic–alpine habitats and their connectivity across the Northern Hemisphere at 30 Ma.** Arctic-alpine habitats are shown in blue, and least cost paths connecting them are depicted as black lines. PC refers to probability of connectivity, which is a summary statistic of the global connectedness of the network defined by arctic-alpine habitat patches (nodes) and least cost paths (edges).

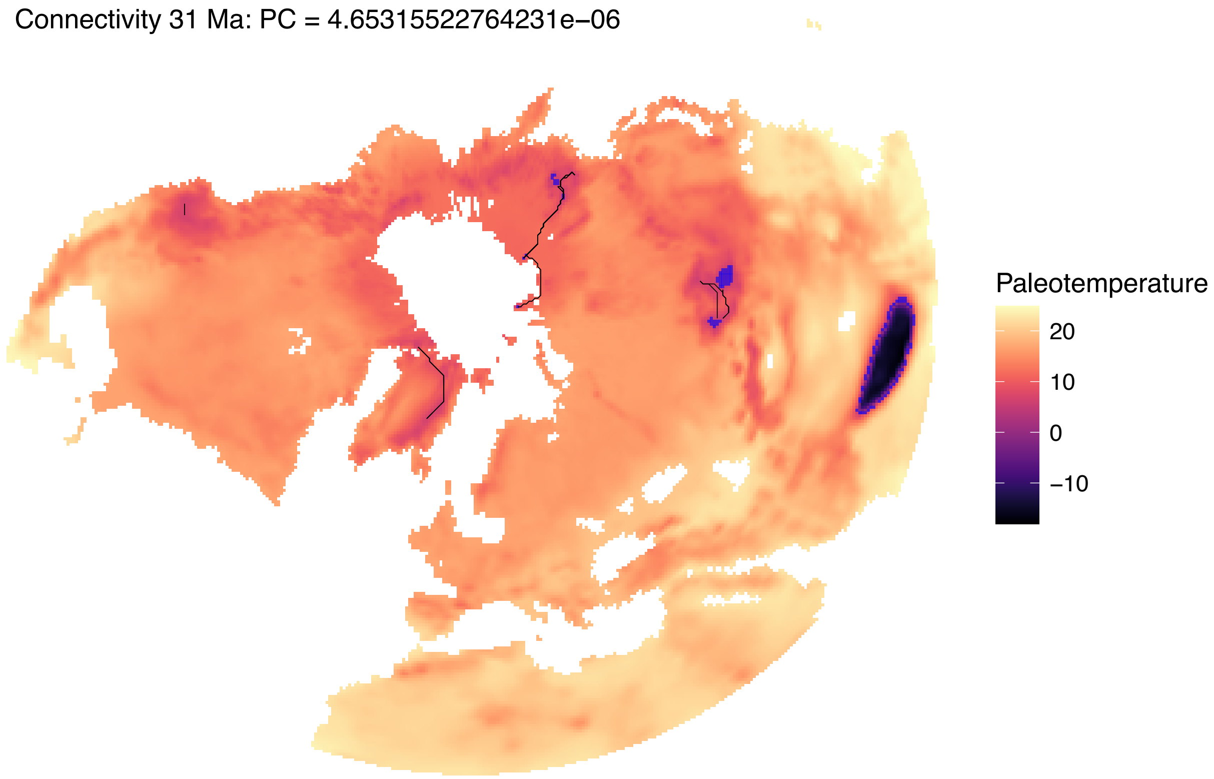

Figure S37: **Estimated distribution of arctic–alpine habitats and their connectivity across the Northern Hemisphere at 31 Ma.** Arctic-alpine habitats are shown in blue, and least cost paths connecting them are depicted as black lines. PC refers to probability of connectivity, which is a summary statistic of the global connectedness of the network defined by arctic-alpine habitat patches (nodes) and least cost paths (edges).

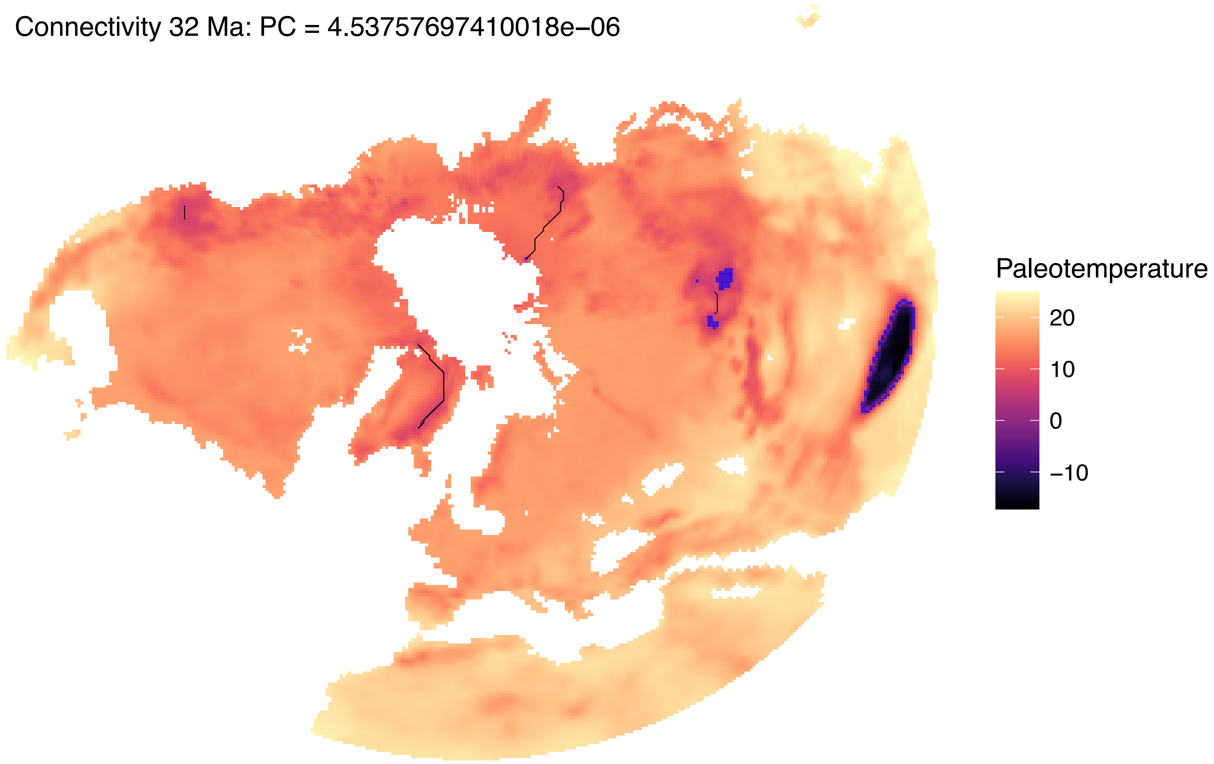

Figure S38: **Estimated distribution of arctic–alpine habitats and their connectivity across the Northern Hemisphere at 32 Ma.** Arctic-alpine habitats are shown in blue, and least cost paths connecting them are depicted as black lines. PC refers to probability of connectivity, which is a summary statistic of the global connectedness of the network defined by arctic-alpine habitat patches (nodes) and least cost paths (edges).

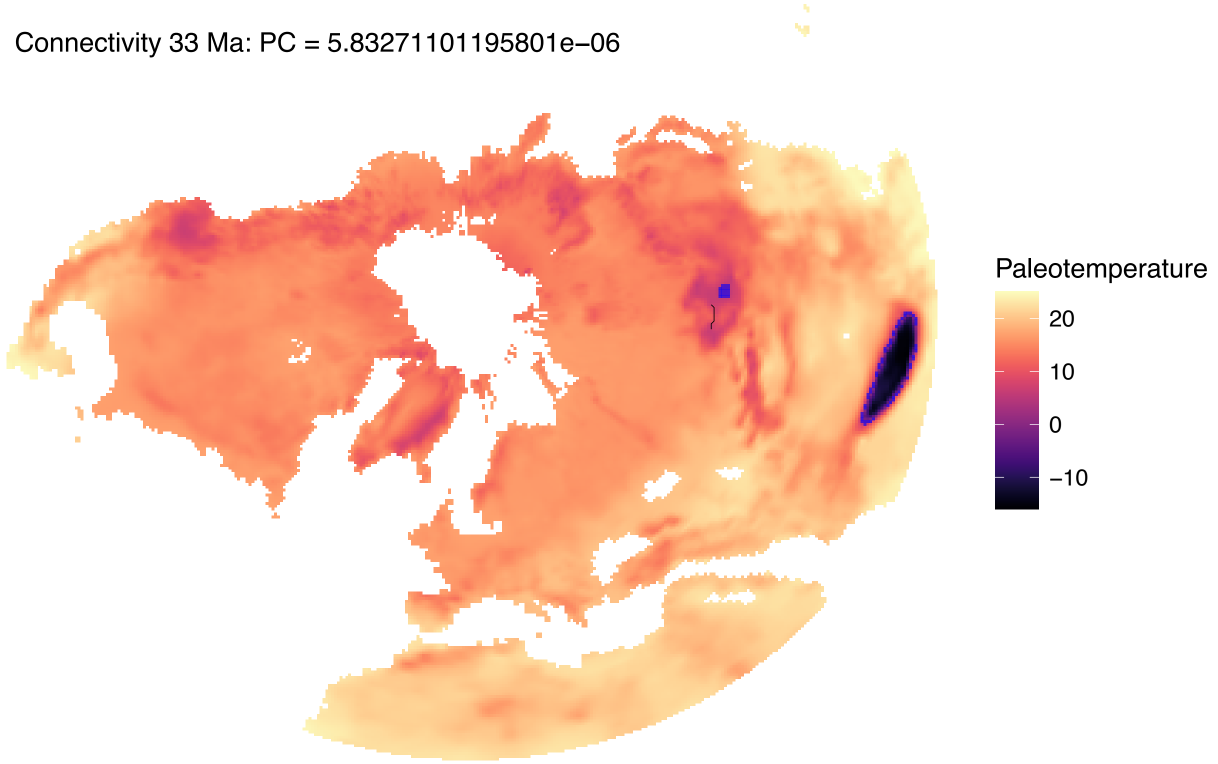

Figure S39: **Estimated distribution of arctic–alpine habitats and their connectivity across the Northern Hemisphere at 33 Ma.** Arctic-alpine habitats are shown in blue, and least cost paths connecting them are depicted as black lines. PC refers to probability of connectivity, which is a summary statistic of the global connectedness of the network defined by arctic-alpine habitat patches (nodes) and least cost paths (edges).

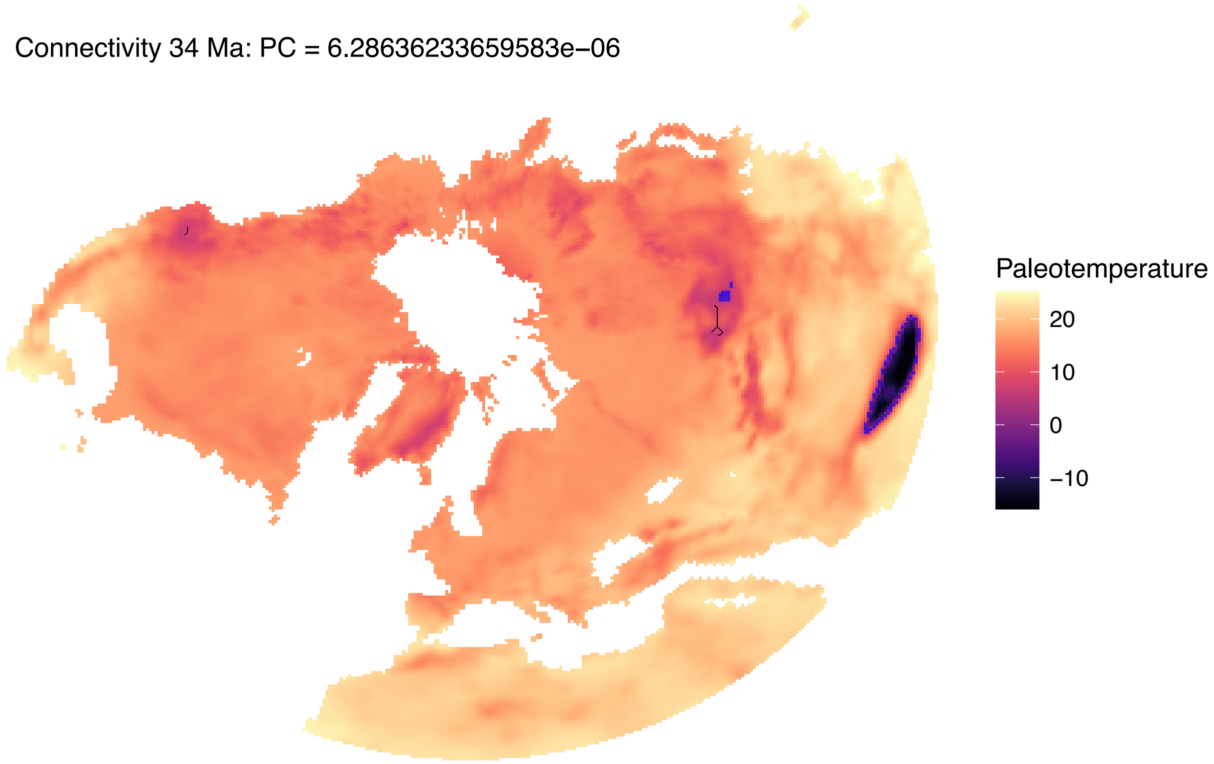

Figure S40: **Estimated distribution of arctic–alpine habitats and their connectivity across the Northern Hemisphere at 34 Ma.** Arctic-alpine habitats are shown in blue, and least cost paths connecting them are depicted as black lines. PC refers to probability of connectivity, which is a summary statistic of the global connectedness of the network defined by arctic-alpine habitat patches (nodes) and least cost paths (edges).

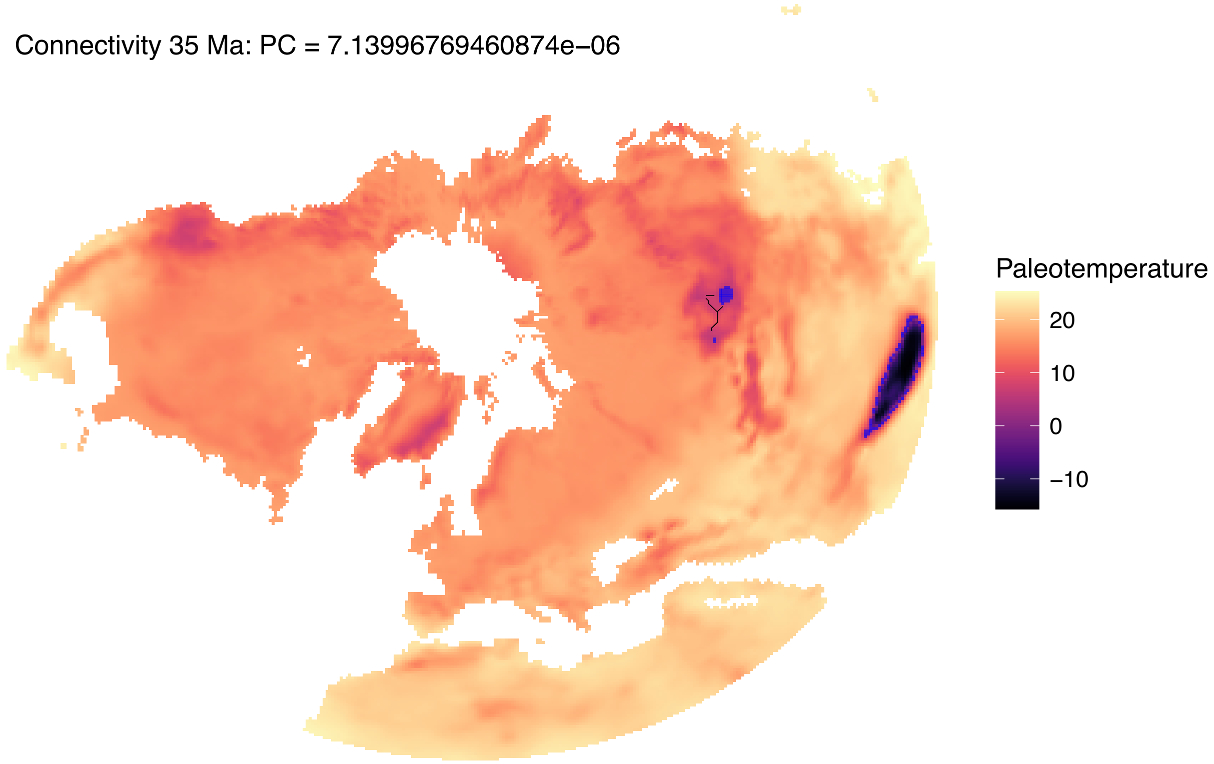

Figure S41: **Estimated distribution of arctic–alpine habitats and their connectivity across the Northern Hemisphere at 35 Ma.** Arctic-alpine habitats are shown in blue, and least cost paths connecting them are depicted as black lines. PC refers to probability of connectivity, which is a summary statistic of the global connectedness of the network defined by arctic-alpine habitat patches (nodes) and least cost paths (edges).

Table S1: **Sampling details for each clade.** Sampling fraction (SF, %) indicates the proportion of described species represented in the phylogeny. Phylogenies constructed in this study are marked *This study*; others cite the original source.

Family	Genus	Sampling	Total	SF(%)	Root age (Ma)	References
Rosaceae	*Alchemilla*	130	795	0.164	20.421	This study
Amaryllidaceae	*Allium*	482	1077	0.448	33.539	(*111*)
Caryophyllaceae	*Arenaria*, *Cerastium*, *Moehringia*, *Stellaria*, *Pseudostellaria*, *Odontostemma*	243	685	0.355	47.157	This study
Campanulaceae	*Campanula*	334	449	0.744	43.808	This study
Caraganeae	*Calophaca*, *Caragana*, *Chesneya*, *Tibetia*, *Gueldenstaedtia*	110	162	0.679	21.445	This study
Cyperaceae	*Carex*	1362	2083	0.654	37.175	(*112*)
Brassicaceae	*Draba*	217	431	0.503	14.018	(*5*)
Poaceae	*Festuca*	193	661	0.292	20.239	This study
Gentianaceae	*Gentiana*	200	341	0.587	29.761	(*113*)
Fabaceae	*Hedysarum*	102	225	0.453	12.814	(*114*)
Saxifragaceae	Heuchereae, Chrysosplenieae	216	292	0.740	55.247	(*115*)
Juncaceae	*Juncus*	109	341	0.320	41.547	This study
Juncaceae	*Luzula*	77	125	0.616	18.402	This study
Liliaceae	*Erythronium*, *Fritillaria*, *Gagea*, *Lilium*, *Tulipa*	394	636	0.619	68.152	This study
Fabaceae	*Oxytropis*	191	608	0.314	14.679	This study
Pedicularis	*Pedicularis*	339	679	0.499	29.303	(*5*)
Poaceae	*Poa*	281	578	0.486	17.389	This study
Rosaceae	*Potentilla*	236	511	0.462	24.578	This study
Primulaceae	*Androsace*, *Dionysia*, *Primula*	404	787	0.513	39.740	(*5*)
Ranunculaceae	*Ranunculus*	310	1750	0.177	29.758	This study
Crassulaceae	*Rhodiola*	51	74	0.689	14.258	(*5*)
Ericaceae	*Rhododendron*	438	1091	0.401	44.751	(*5*)
Salicaceae	*Salix*	226	471	0.480	34.272	This study
Asteraceae	*Saussurea*	166	516	0.322	9.639	(*116*)
Saxifragaceae	*Saxifraga*	340	477	0.713	49.714	(*11*)
Caryophyllaceae	*Silene*	423	904	0.468	23.992	This study
Fabaceae	*Trifolium*	215	299	0.719	20.763	This study
Asteraceae	*Artemisia*	108	499	0.216	32.649	(*43*)
Asteraceae	*Erigeron*	112	456	0.246	13.189	(*43*)
Plantaginaceae	*Veronica*	92	464	0.198	14.269	(*117*)
Poaceae	*Stipa*	46	146	0.315	12.887	This study
Iridaceae	*Crocus*	138	248	0.556	16.138	This study
Lamiaceae	*Nepeta*	102	295	0.346	17.738	This study
Lamiaceae	*Dracocephalum*	69	89	0.775	17.269	This study

Table S2: **Cumulative distinct assembly events in the boreal-arctic region over time.** Estimates are based on 1,000 replicated joint biogeographic histories that account for uncertainty in ancestral range and biome reconstructions. Median values and interquartile ranges (25th to 75th percentiles) are reported.

	Colonization			Niche expansion			*in situ* speciation			Subset speciation		
Time (Ma)	0.25 quantile	Median	0.75 quantile	0.25 quantile	Median	0.75 quantile	0.25 quantile	Median	0.75 quantile	0.25 quantile	Median	0.75 quantile
[0, 1)	328	340	353	277	290	305	152	160	168	221	231	242
[1, 2)	231	242	254	197	208	220	121	129	136	174	183	192
[2, 3)	169	179	190	142	153	162	86	92	100	127	135	142
[3, 4)	126	135	145	105	113	121	58	63	69	88	95	102
[4, 5)	96	104	114	78	85	92	36	41	46	63	69	75
[5, 6)	74	81	90	59	66	72	18	22	26	40	44	49
[6, 7)	57	64	71	47	52	58	14	18	21	29	33	37
[7, 8)	44.75	50	57	38	42	48	11	14	17	20	23	27
[8, 9)	35	40	47	30	35	40	8	11	13.25	14	16	20
[9, 10)	28	33	39	25	29	34	7	9	12	10	13	15
[10, 11)	23	27	33	21	25	29	6	8	11	8	10	13
[11, 12)	19	23	28	18	22	25	5	7	9	6	8	10
[12, 13)	15	19	24	16	19	22	4	6	8	5	7	9
[13, 14)	12	16	20	14	17	20	4	5	7	4	6	8
[14, 15)	10	13	18	12	15	18	3	5	7	4	5	7
[15, 16)	8	12	15	10	13	16	3	4	6	3	4	6
[16, 17)	7	10	14	9	11	14	2	4	5	2	3	5
[17, 18)	6	9	12	8	10	13	2	3	5	2	3	5
[18, 19)	5	7	10	7	9	11	2	3	4	1	3	4
[19, 20)	4	6	9	6	8	10	1	2	3	1	2	3
[20, 21)	3	6	8	5	7	9	1	2	3	1	2	3
[21, 22)	3	5	7	5	6	8	1	2	3	1	2	3
[22, 23)	2	4	6	4	6	8	1	1	2	1	1	2
[23, 24)	2	4	5	4	5	7	1	1	2	1	1	2
[24, 25)	2	3	5	3	5	6	1	1	2	0	1	2
[25, 26)	1	3	4	3	4	6	1	1	2	0	1	2
[26, 27)	1	2	4	3	4	5	1	1	2	0	1	2
[27, 28)	1	2	3	2	3	5	0	1	2	0	1	2
[28, 29)	1	1	3	2	3	4	0	1	2	0	1	1
[29, 30)	0	1	2	1	3	4	0	1	2	0	1	1
[30, 31)	0	1	2	1	2	3	0	0	1	0	0	1
[31, 32)	0	1	2	1	2	3	0	0	1	0	0	1
[32, 33)	0	1	1	1	2	3	0	0	1	0	0	1
[33, 34)	0	0	1	1	1	2	0	0	1	0	0	1
[34, 35)	0	0	1	0	1	2	0	0	0	0	0	0
[35, 36)	0	0	1	0	1	2	0	0	0	0	0	0
[36, 37)	0	0	1	0	1	2	0	0	0	0	0	0
[37, 38)	0	0	1	0	1	2	0	0	0	0	0	0
[38, 39)	0	0	1	0	1	1	0	0	0	0	0	0
[39, 40)	0	0	1	0	1	1	0	0	0	0	0	0
[40, 41)	0	0	0	0	1	1	0	0	0	0	0	0
[41, 42)	0	0	0	0	1	1	0	0	0	0	0	0
[42, 43)	0	0	0	0	0	1	0	0	0	0	0	0
[43, 44)	0	0	0	0	0	1	0	0	0	0	0	0
[44, 45)	0	0	0	0	0	1	0	0	0	0	0	0
[45, 46)	0	0	0	0	0	1	0	0	0	0	0	0
[46, 47)	0	0	0	0	0	1	0	0	0	0	0	0

Table S3: **Cumulative distinct assembly events in the European Alpine system over time.** Estimates are based on 1,000 replicated joint biogeographic histories that account for uncertainty in ancestral range and biome reconstructions. Median values and interquartile ranges (25th to 75th percentiles) are reported.

	Colonization			Niche expansion			*in situ* speciation			Subset speciation		
Time (Ma)	0.25 quantile	Median	0.75 quantile	0.25 quantile	Median	0.75 quantile	0.25 quantile	Median	0.75 quantile	0.25 quantile	Median	0.75 quantile
[0, 1)	152	160	168	259	273.5	288.25	104	112	120	70	76	82
[1, 2)	107	115	123	198	211	223	92	99	107	61	67	73
[2, 3)	77	84	91	156	166	177.25	78	85	93	51	56	62
[3, 4)	57	63	70	124	132	142	64	71	78	42	47	52
[4, 5)	44	49	55	98	107	115	48	54	60	35	39	44
[5, 6)	34	39	44	79	86	94	34	39	45	27	31	35
[6, 7)	27	31	36	64	70	77	25	30	35	20	24	27
[7, 8)	22	26	30	52	58	64	20	24	29	17	20	23
[8, 9)	18	21	25	42	48	53	16	19	23	14	17	20
[9, 10)	15	18	22	35	40	45	13	16	20	12	14	17
[10, 11)	12	15	19	28	33	38	10	13	16	10	12	15
[11, 12)	10	13	16	23	27	32	8	10	13	7	9	12
[12, 13)	8	11	14	19	23	27	7	9	12	7	9	11
[13, 14)	7	9	12	15	19	22	6	8	11	5	6	9
[14, 15)	5	8	10	12.75	16	19	4	6	8	4	6	7
[15, 16)	5	7	9	10	13	15	3	4	6	3	4	6
[16, 17)	4	6	8	8	10	13	2	4	5	3	4	5
[17, 18)	3	5	7	6	8	10.25	2	3	5	2	3	4
[18, 19)	3	4	6	5	7	9	1	2	4	1	2	3
[19, 20)	2	3	5	4	5	7	1	2	3	1	2	3
[20, 21)	2	3	4	3	5	6	0	1	2	1	1	2
[21, 22)	2	3	4	2	4	5	0	1	2	1	1	2
[22, 23)	1	2	4	2	3	5	0	1	2	0	1	2
[23, 24)	1	2	3	2	3	4	0	1	2	0	1	2
[24, 25)	1	2	3	1	2	3	0	0	1	0	1	1
[25, 26)	1	2	3	1	2	3	0	0	1	0	1	1
[26, 27)	1	1	2	1	1	2	0	0	1	0	1	1
[27, 28)	1	1	2	0	1	2	0	0	1	0	0	1
[28, 29)	0	1	2	0	1	2	0	0	0	0	0	1
[29, 30)	0	1	1	0	1	1	0	0	0	0	0	1
[30, 31)	0	1	1	0	0	1	0	0	0	0	0	0
[31, 32)	0	0	1	0	0	1	0	0	0	0	0	0
[32, 33)	0	0	1	0	0	1	0	0	0	0	0	0
[33, 34)	0	0	1	0	0	1	0	0	0	0	0	0
[34, 35)	0	0	0	0	0	1	0	0	0	0	0	0

Table S4: **Cumulative distinct assembly events in the Tibeto-Himalayan-Hengduan region over time.** Estimates are based on 1,000 replicated joint biogeographic histories that account for uncertainty in ancestral range and biome reconstructions. Median values and interquartile ranges (25th to 75th percentiles) are reported.

	Colonization			Niche expansion			*in situ* speciation			Subset speciation		
Time (Ma)	0.25 quantile	Median	0.75 quantile	0.25 quantile	Median	0.75 quantile	0.25 quantile	Median	0.75 quantile	0.25 quantile	Median	0.75 quantile
[0, 1)	191	200	209	331	347	361	582	594	608	155	164	173
[1, 2)	136	144	152	262	274	287	496	508	521	131	139	147
[2, 3)	101	109	117	210	220	232	395	406	418	102	109	117
[3, 4)	78	84	92	170	180	190	322	331	342	82	89	95
[4, 5)	61	66	73	138	146.5	156	256	264	274	63	69	76
[5, 6)	47	52	58	113	120	127	203	211.5	220	46	52	57
[6, 7)	36	41	46	91	98	105	158.75	166	173	35	40	44
[7, 8)	28	33	38	75	81	87	130	136	143	28	32	36
[8, 9)	23	27	32	62	67	73	99	106	112	22	26	30
[9, 10)	19	22	27	51	56	60	77	83	89	19	22	26
[10, 11)	15	19	23	42	46	50	60	65	70	15	18	21
[11, 12)	13	16	20	34	37	41	48	53	57	12	15	18
[12, 13)	11	14	17.25	27	31	35	36	40	44	11	13	16
[13, 14)	9	12	15	22	25	29	30	34	38	8	11	13
[14, 15)	7	10	13	18	20	24	24	27	31	6	8	11
[15, 16)	6	8	11	14	17	19	19	22	25	4	5	8
[16, 17)	5	7	10	11	14	16	15	18	21	3	5	7
[17, 18)	4	6	8	9	11	13	12	14	17	3	4	6
[18, 19)	3	5	7	7	9	11	11	13	15	3	4	6
[19, 20)	3	4	6	6	8	9	7	9	12	2	3	5
[20, 21)	2	4	6	5	6	8	6	8	10	2	3	5
[21, 22)	2	3	5	4	5	6	6	7	9	2	3	4
[22, 23)	1	3	4	3	4	5	5	6	8	2	2	4
[23, 24)	1	2	4	2	3	4	4	6	7	1	2	4
[24, 25)	1	2	3	1	2	4	3	5	6	1	2	3
[25, 26)	0	1	3	1	2	3	2	3	4	1	2	3
[26, 27)	0	1	2	1	1	2	1	2	3	1	2	2
[27, 28)	0	1	2	0	1	2	0	1	1	1	2	2
[28, 29)	0	1	1	0	1	2	0	0	1	1	1	1
[29, 30)	0	0	1	0	1	1	0	0	1	1	1	1
[30, 31)	0	0	1	0	0	1	0	0	0	0	0	0
[31, 32)	0	0	1	0	0	1	0	0	0	0	0	0
[32, 33)	0	0	1	0	0	1	0	0	0	0	0	0
[33, 34)	0	0	0	0	0	1	0	0	0	0	0	0
[34, 35)	0	0	0	0	0	1	0	0	0	0	0	0
[35, 36)	0	0	0	0	0	1	0	0	0	0	0	0
[36, 37)	0	0	0	0	0	1	0	0	0	0	0	0
[37, 38)	0	0	0	0	0	1	0	0	0	0	0	0
[38, 39)	0	0	0	0	0	1	0	0	0	0	0	0
[39, 40)	0	0	0	0	0	1	0	0	0	0	0	0
[40, 41)	0	0	0	0	0	1	0	0	0	0	0	0

Table S5: **Cumulative distinct assembly events in the mountains of western North America over time.** Estimates are based on 1,000 replicated joint biogeographic histories that account for uncertainty in ancestral range and biome reconstructions. Median values and interquartile ranges (25th to 75th percentiles) are reported.

	Colonization			Niche expansion			*in situ* speciation			Subset speciation		
Time (Ma)	0.25 quantile	Median	0.75 quantile	0.25 quantile	Median	0.75 quantile	0.25 quantile	Median	0.75 quantile	0.25 quantile	Median	0.75 quantile
[0, 1)	208	215	223	176	186	197	88	95	103	92	98	104
[1, 2)	135	142	149	120	129	138	73	80	87.25	72	77	83
[2, 3)	91	97	104	86	94	101	58	64	71	49	54	59
[3, 4)	62	67	73	63	70	77	41	47	53	34	38	42
[4, 5)	43	48	53	49	54	60	29	34	39	24	27	31
[5, 6)	32	36	40	38	43	48	23	27	32	16	19	22
[6, 7)	24	27	31	30	35	40	17	20	24	10	13	16
[7, 8)	17.75	21	24	24	28	33	13	16	20	6	8	11
[8, 9)	13	16	19	20	23	27	12	15	19	5	7	9
[9, 10)	10	13	15	16	20	23	8	11	15	4	5	7
[10, 11)	8	11	13	13	17	20	6	9	11	3	4	6
[11, 12)	6	9	11	11	14	17	5	7	10	2	3	4
[12, 13)	5	7	9	9	12	14	3	5	8	1	2	4
[13, 14)	4	6	8	8	10	12.25	3	5	8	1	2	3
[14, 15)	3	5	6.25	6	9	11	3	5	7	1	2	3
[15, 16)	3	4	6	6	8	10	2	4	7	1	2	3
[16, 17)	2	3	5	5	7	9	2	4	7	0	1	3
[17, 18)	2	3	4	4	6	8	2	4	6	0	1	3
[18, 19)	1	2	4	4	5	7	2	4	6	0	1	2
[19, 20)	1	2	3	3	5	7	2	4	6	0	1	2
[20, 21)	1	2	3	3	5	6	2	3	6	0	1	2
[21, 22)	0	1	3	3	4	6	2	3	6	0	1	2
[22, 23)	0	1	2	2	4	5	2	3	6	0	1	2
[23, 24)	0	1	2	2	3	5	1	3	5	0	1	2
[24, 25)	0	1	2	2	3	5	1	2	4	0	0	1
[25, 26)	0	1	1	2	3	4	0	2	3	0	0	1
[26, 27)	0	0	1	1	2	4	0	1	3	0	0	1
[27, 28)	0	0	1	1	2	3	0	1	3	0	0	1
[28, 29)	0	0	1	1	2	3	0	1	3	0	0	1
[29, 30)	0	0	1	1	2	3	0	1	2	0	0	1
[30, 31)	0	0	1	1	2	3	0	1	2	0	0	1
[31, 32)	0	0	1	1	1	3	0	0	2	0	0	1
[32, 33)	0	0	1	1	1	2	0	0	2	0	0	1
[33, 34)	0	0	0	0	1	2	0	0	2	0	0	1
[34, 35)	0	0	0	0	1	2	0	0	2	0	0	1
[35, 36)	0	0	0	0	1	2	0	0	2	0	0	1
[36, 37)	0	0	0	0	1	2	0	0	1	0	0	1
[37, 38)	0	0	0	0	1	1	0	0	1	0	0	0
[38, 39)	0	0	0	0	1	1	0	0	1	0	0	0
[39, 40)	0	0	0	0	0	1	0	0	1	0	0	0
[40, 41)	0	0	0	0	0	1	0	0	0	0	0	0
[41, 42)	0	0	0	0	0	1	0	0	0	0	0	0
[42, 43)	0	0	0	0	0	1	0	0	0	0	0	0
[43, 44)	0	0	0	0	0	1	0	0	0	0	0	0
[44, 45)	0	0	0	0	0	1	0	0	0	0	0	0

Table S6: **Cumulative distinct assembly events in the Irano-Turanian-Caucasus region over time.** Estimates are based on 1,000 replicated joint biogeographic histories that account for uncertainty in ancestral range and biome reconstructions. Median values and interquartile ranges (25th to 75th percentiles) are reported.

	Colonization			Niche expansion			*in situ* speciation			Subset speciation		
Time (Ma)	0.25 quantile	Median	0.75 quantile	0.25 quantile	Median	0.75 quantile	0.25 quantile	Median	0.75 quantile	0.25 quantile	Median	0.75 quantile
[0, 1)	206	217	229	212	222	234	84.75	92	99	51	57	62
[1, 2)	163	173	184	158	169	178	80	87	94	46	51	56
[2, 3)	124	133	143	119	127	136	57	63	70	37	42	47
[3, 4)	94	103	111	90	97	105	41	46	53	31	35	39
[4, 5)	73	79	88	70	76	82	32	37	42	24	28	32
[5, 6)	57	63	70	54	60	66	25	30	34	20	24	27
[6, 7)	44	51	57	42	47	52	16	19	23	16	18	21
[7, 8)	35	41	48	33	38	42	12	15	18	12	15	17.25
[8, 9)	28.75	34	40	27	31	35	8	11	14	10	13	15
[9, 10)	23	28	33	21	25	29	7	9	12	9	10	13
[10, 11)	19	23	28	17	21	24	4	6	9	7	9	11
[11, 12)	15	19	24	14	16	20	2	4	6	5	7	9
[12, 13)	12	16	20	11	13	16	1	2	4	4	5	7
[13, 14)	10	13	17	9	11	14	1	2	3	2	4	5
[14, 15)	8	11	15	7	9	11	1	2	3	2	3	5
[15, 16)	7	10	13	5	7	10	0	1	3	2	3	4
[16, 17)	6	8	11	4	6	8	0	1	2	1	2	3
[17, 18)	5	7	10	3	5	7	0	1	2	1	2	3
[18, 19)	4	6	8	3	4	6	0	1	2	0	1	3
[19, 20)	3	5	7	2	3.5	5	0	0	1	0	1	2
[20, 21)	2	4	6	2	3	4	0	0	1	0	1	2
[21, 22)	2	4	6	1	3	4	0	0	1	0	1	2
[22, 23)	2	3	5	1	2	3	0	0	1	0	0	1
[23, 24)	1	3	4	1	2	3	0	0	1	0	0	1
[24, 25)	1	2	4	1	2	3	0	0	1	0	0	1
[25, 26)	1	2	3	1	1	3	0	0	1	0	0	1
[26, 27)	1	2	3	0	1	2	0	0	1	0	0	1
[27, 28)	0	1	2	0	1	2	0	0	1	0	0	1
[28, 29)	0	1	2	0	1	2	0	0	0	0	0	1
[29, 30)	0	1	2	0	1	1	0	0	0	0	0	0
[30, 31)	0	1	1	0	1	1	0	0	0	0	0	0
[31, 32)	0	0	1	0	0	1	0	0	0	0	0	0
[32, 33)	0	0	1	0	0	1	0	0	0	0	0	0
[33, 34)	0	0	1	0	0	1	0	0	0	0	0	0
[34, 35)	0	0	0	0	0	1	0	0	0	0	0	0
[35, 36)	0	0	0	0	0	1	0	0	0	0	0	0
[36, 37)	0	0	0	0	0	1	0	0	0	0	0	0

Table S7: **Cumulative distinct assembly events in the Tianshan-Pamir region region over time.** Estimates are based on 1,000 replicated joint biogeographic histories that account for uncertainty in ancestral range and biome reconstructions. Median values and interquartile ranges (25th to 75th percentiles) are reported.

	Colonization			Niche expansion			*in situ* speciation			Subset speciation		
Time (Ma)	0.25 quantile	Median	0.75 quantile	0.25 quantile	Median	0.75 quantile	0.25 quantile	Median	0.75 quantile	0.25 quantile	Median	0.75 quantile
[0, 1)	235	245	254.25	114	121	129	26	30	34	37	41	45
[1, 2)	164	173	181	81	88	95	22	26	30	30	34	38
[2, 3)	119	126	135	60	66	72	19	22	26	24	27	30
[3, 4)	87	95	102	45	50	56	15	18	21	19	22	25
[4, 5)	64	70	78	35	39	44	11	13	17	12	15	18
[5, 6)	49	55	62	27	30	34	10	13	16	11	13	16
[6, 7)	38	44	50	20	23	26	6	8	10	8	10	13
[7, 8)	30	35	40	14	17	21	3	5	6	5	7	9
[8, 9)	23	28	32	11	14	16	2	3	5	4	5	7
[9, 10)	19	22	27	8	11	14	1	2	3	3	4	6
[10, 11)	15	18	22	6	9	11	1	2	3	2	3	5
[11, 12)	12	15	19	5	7	9	0	1	2	1	2	4
[12, 13)	9	12	16	4	6	7	0	1	2	1	2	3
[13, 14)	8	10	13	3	5	6	0	0	1	0	1	2
[14, 15)	6	8	11	2	4	5	0	0	1	0	1	2
[15, 16)	5	7	9	2	3	5	0	0	1	0	1	1
[16, 17)	4	6	8	1	2	4	0	0	1	0	0	1
[17, 18)	3	5	7	1	2	3	0	0	1	0	0	1
[18, 19)	2	4	6	1	2	3	0	0	0	0	0	1
[19, 20)	2	3	5	1	1	2	0	0	0	0	0	1
[20, 21)	2	3	5	0	1	2	0	0	0	0	0	1
[21, 22)	1	3	4	0	1	2	0	0	0	0	0	1
[22, 23)	1	2	4	0	1	2	0	0	0	0	0	1
[23, 24)	1	2	3	0	1	2	0	0	0	0	0	0
[24, 25)	1	2	3	0	1	1	0	0	0	0	0	0
[25, 26)	0	1	2	0	0	1	0	0	0	0	0	0
[26, 27)	0	1	2	0	0	1	0	0	0	0	0	0
[27, 28)	0	1	2	0	0	1	0	0	0	0	0	0
[28, 29)	0	1	2	0	0	1	0	0	0	0	0	0
[29, 30)	0	1	1	0	0	1	0	0	0	0	0	0
[30, 31)	0	0	1	0	0	1	0	0	0	0	0	0
[31, 32)	0	0	1	0	0	1	0	0	0	0	0	0
[32, 33)	0	0	1	0	0	1	0	0	0	0	0	0
[33, 34)	0	0	1	0	0	1	0	0	0	0	0	0

Table S8: **Consistency of automatic European alpine plant assignments with floristic validation.** Values show the number of true positives (TP), false positives (FP, overestimation), and false negatives (FN, underestimation), along with overall precision (TP / (TP+FP)), consistency (TP / Alpine), overestimation, and underestimation rates. tlh (distance to treeline) = treeline-based estimate; mcb = bioclimatic belt-based estimate. Validation columns of the alpine biome assignment are based on the independent floristic source (Flora Alpina monograph (*35*)).

Threshold	Alpine	TP	FP	FN	Precision	Consistency	Overestimation	Underestimation
25%_tlh	258	152	20	106	0.88	0.59	0.08	0.41
5%_tlh	258	214	58	44	0.79	0.83	0.22	0.17
25%_mcb	258	105	6	153	0.95	0.41	0.02	0.59
5%_mcb	258	196	34	62	0.85	0.76	0.13	0.24

REFERENCES

1. C. Rahbek, M. K. Borregaard, A. Antonelli, R. K. Colwell, B. G. Holt, D. Nogues-Bravo, C. M. Ø. Rasmussen, K. Richardson, M. T. Rosing, R. J. Whittaker, J. Fjeldså, Building mountain biodiversity: Geological and evolutionary processes. *Science* **365**, 1114–1119 (2019).

2. A. Antonelli, W. D. Kissling, S. G. A. Flantua, M. A. Bermúdez, A. Mulch, A. N. Muellner-Riehl, H. Kreft, H. P. Linder, C. Badgley, J. Fjeldså, S. A. Fritz, C. Rahbek, F. Herman, H. Hooghiemstra, C. Hoorn, Geological and climatic influences on mountain biodiversity. *Nat. Geosci.* **11**, 718–725 (2018).

3. T. M. Smiley, A. Bahadori, E. T. Rasbury, W. E. Holt, C. Badgley, Tectonic extension and paleoelevation influence mammalian diversity dynamics in the Basin and Range Province of western North America. *Sci. Adv.* **10**, eadn6842 (2024).

4. C. Badgley, T. M. Smiley, R. Terry, E. B. Davis, L. R. G. DeSantis, D. L. Fox, S. S. B. Hopkins, T. Jezkova, M. D. Matocq, N. Matzke, J. L. McGuire, A. Mulch, B. R. Riddle, V. L. Roth, J. X. Samuels, C. A. E. Strömberg, B. J. Yanites, Biodiversity and topographic complexity: Modern and geohistorical perspectives. *Trends Ecol. Evol.* **32**, 211–226 (2017).

5. W.-N. Ding, R. H. Ree, R. A. Spicer, Y.-W. Xing, Ancient orogenic and monsoon-driven assembly of the world's richest temperate alpine flora. *Science* **369**, 578–581 (2020).

6. E. Marder, T. M. Smiley, B. J. Yanites, K. Kravitz, Direct effects of mountain uplift and topography on biodiversity. *Science* **387**, 1287–1291 (2025).

7. Y. Xing, R. H. Ree, Uplift-driven diversification in the Hengduan Mountains, a temperate biodiversity hotspot. *Proc. Natl. Acad. Sci. U.S.A.* **114**, E3444–E3451 (2017).

8. S. Klaus, R. J. Morley, M. Plath, Y.-P. Zhang, J.-T. Li, Biotic interchange between the Indian subcontinent and mainland Asia through time. *Nat. Commun.* **7**, 12132 (2016).

9. C. D. Bacon, D. Silvestro, C. Jaramillo, B. T. Smith, P. Chakrabarty, A. Antonelli, Biological evidence supports an early and complex emergence of the Isthmus of Panama. *Proc. Natl. Acad. Sci. U.S.A.* **112**, 6110–6115 (2015).

10. B. G. Kuhnhäuser, C. D. Bates, J. Dransfield, C. Geri, A. Henderson, S. Julia, J. Y. Lim, R. J. Morley, H. Rustiami, R. J. Schley, S. Bellot, G. Chomicki, W. L. Eiserhardt, S. J. Hiscock, W. J. Baker, Island geography drives evolution of rattan palms in tropical Asian rainforests. *Science* **387**, 1204–1209 (2025).

11. T. Carruthers, M. S. Moerland, J. Ebersbach, A. Favre, R. A. Folk, J. A. Hawkins, A. N. Muellner-Riehl, M. Röser, D. E. Soltis, N. Tkach, W. J. Baker, J. M. de Vos, W. L. Eiserhardt, Repeated upslope biome shifts in *Saxifraga* during late-Cenozoic climate cooling. *Nat. Commun.* **15**, 1100 (2024).

12. O. Hagen, L. Vaterlaus, C. Albouy, A. Brown, F. Leugger, R. E. Onstein, C. N. de Santana, C. R. Scotese, L. Pellissier, Mountain building, climate cooling and the richness of cold-adapted plants in the Northern Hemisphere. *J. Biogeogr.* **46**, 1792–1807 (2019).

13. C. Körner, "The alpine life zone," in *Alpine Plant Life: Functional Plant Ecology of High Mountain Ecosystems* (Springer Nature, 3rd ed., 2021), pp. 23–51.

14. R. Testolin, F. Attorre, P. Borchardt, R. F. Brand, H. Bruelheide, M. Chytrá, M. De Sanctis, J. Dolezal, M. Finckh, S. Haider, A. Hemp, U. Jandt, M. Kessler, A. Y. Korolyuk, J. Lenoir, N. Makunina, G. P. Malanson, D. B. Montesinos-Tubée, J. Noroozi, A. Nowak, R. K. Peet, G. Peyre, F. M. Sabatini, J. Šibík, P. Sklenář, S. P. Sylvester, K. Vassilev, R. Virtanen, W. Willner, S. K. Wiser, E. G. Zibzeev, B. Jiménez-Alfaro, Global patterns and drivers of alpine plant species richness. *Glob. Ecol. Biogeogr.* **30**, 1218–1231 (2021).

15. L. Ding, P. Kapp, F. Cai, C. N. Garzione, Z. Xiong, H. Wang, C. Wang, Timing and mechanisms of Tibetan Plateau uplift. *Nat. Rev. Earth Environ.* **3**, 652–667 (2022).

16. G. Zhang, Y. Tian, R. Li, X. Shen, Z. Zhang, X. Sun, D. Chen, Progressive tectonic evolution from crustal shortening to mid-lower crustal expansion in the southeast Tibetan Plateau: A synthesis of structural and thermochronological insights. *Earth Sci. Rev.* **226**, 103951 (2022).

17. F. Liu, M. Wang, H. Liu, R. Ni, The Cenozoic spatiotemporal exhumation of the SE Tibetan Plateau: Insight from the data mining and modeling of low-temperature thermochronology. *Front. Earth Sci.* **11**, 1164733 (2023).

18. Z. Song, S. Wan, Z. Yu, M. Yu, C. Colin, Y. Tang, J. Zhang, H. Jin, D. Zhao, X. Shi, A. Li, The major uplift in Himalayas was no earlier than the Miocene: Evidence from marine sediment record in the Bay of Bengal. *Palaeogeogr. Palaeoclimatol. Palaeoecol.* **648**, 112275 (2024).

19. J. Kuhlemann, W. Frisch, B. Szekely, I. Dunkl, M. Kazmer, Post-collisional sediment budget history of the Alps: Tectonic versus climatic control. *Int. J. Earth Sci.* **91**, 818–837 (2002).

20. J. Kuhlemann, Paleogeographic and paleotopographic evolution of the Swiss and Eastern Alps since the Oligocene. *Global Planet. Change* **58**, 224–236 (2007).

21. E. Krsnik, K. Methner, M. Campani, S. Botsyun, S. G. Mutz, T. A. Ehlers, O. Kempf, J. Fiebig, F. Schlunegger, A. Mulch, Miocene high elevation in the Central Alps. *Solid Earth* **12**, 2615–2631 (2021).

22. T. F. Schildgen, D. Cosentino, B. Bookhagen, S. Niedermann, C. Yıldırım, H. Echtler, H. Wittmann, M. R. Strecker, Multi-phased uplift of the southern margin of the Central Anatolian plateau, Turkey: A record of tectonic and upper mantle processes. *Earth Planet. Sci. Lett.* **317-318**, 85–95 (2012).

23. Y. Rolland, Caucasus collisional history: Review of data from East Anatolia to West Iran. *Gondw. Res.* **49**, 130–146 (2017).

24. J. Mosar, T. Kangarli, M. Bochud, U. A. Glasmacher, A. Rast, M.-F. Brunet, M. Sosson, Cenozoic-recent tectonics and uplift in the Greater Caucasus: A perspective from Azerbaijan. *Geol. Soc. Spec. Publ.* **340**, 261–280 (2010).

25. R. Lanari, A. Boutoux, C. Faccenna, F. Herman, S. D. Willett, P. Ballato, Cenozoic exhumation in the Mediterranean and the Middle East. *Earth Sci. Rev.* **237**, 104328 (2023).

26. P. Molin, A. Sembroni, P. Ballato, C. Faccenna, The uplift of an early stage collisional plateau unraveled by fluvial network analysis and river longitudinal profile inversion: The case of the Eastern Anatolian Plateau. *Tectonics* **42**, e2022TC007737 (2023).

27. S. Yu, W. Chen, N. J. Evans, B. I. A. McInnes, J. Yin, J. Sun, J. Li, B. Zhang, Cenozoic uplift, exhumation and deformation in the north Kuqa Depression, China as constrained by (U-Th)/He thermochronometry. *Tectonophysics* **630**, 166–182 (2014).

28. J. P. Colgan, C. D. Henry, Rapid middle Miocene collapse of the Mesozoic orogenic plateau in north-central Nevada. *Int. Geol. Rev.* **51**, 920–961 (2009).

29. C. P. Chamberlain, H. T. Mix, A. Mulch, M. T. Hren, M. L. Kent-Corson, S. J. Davis, T. W. Horton, S. A. Graham, The Cenozoic climatic and topographic evolution of the western North American Cordillera. *Am. J. Sci.* **312**, 213–262 (2012).

30. A. Bahadori, W. E. Holt, Geodynamic evolution of southwestern North America since the Late Eocene. *Nat. Commun.* **10**, 5213 (2019).

31. A. Bahadori, W. E. Holt, R. Feng, J. Austermann, K. M. Loughney, T. Salles, L. Moresi, R. Beucher, N. Lu, L. M. Flesch, C. M. Calvelage, E. T. Rasbury, D. M. Davis, A. R. Potochnik, W. B. Ward, K. Hatton, S. S. B. Haq, T. M. Smiley, K. M. Wooton, C. Badgley, Coupled influence of tectonics, climate, and surface processes on landscape evolution in southwestern North America. *Nat. Commun.* **13**, 4437 (2022).

32. T. Westerhold, N. Marwan, A. J. Drury, D. Liebrand, C. Agnini, E. Anagnostou, J. S. K. Barnet, S. M. Bohaty, D. De Vleeschouwer, F. Florindo, T. Frederichs, D. A. Hodell, A. E. Holbourn, D. Kroon, V. Lauretano, K. Littler, L. J. Lourens, M. Lyle, H. Pälike, U. Röhl, J. Tian, R. H. Wilkens, P. A. Wilson, J. C. Zachos, An astronomically dated record of Earth's climate and its predictability over the last 66 million years. *Science* **369**, 1383–1387 (2020).

33. L. M. Wootton, F. C. Boucher, C. Pouchon, C. Roquet, E. Coissac, J. Renaud, I. G. Alsos, P. G. Valla, L. Husson, M. Bernet, C. Perrier, R. Douzet, M. Rome, J.-G. Valay, A. Alberti, F. Denoeud, N. E. Zimmermann, P. Wincker, W. Thuiller, S. Lavergne, The late rise of sky-island vegetation in the European Alps. *Nat. Plants* **11**, 1142–1153 (2025).

34. B. Xu, Z.-M. Li, H. Sun, Plant diversity and floristic characters of the alpine subnival belt flora in the Hengduan Mountains, SW China. *J. Syst. Evol.* **52**, 271–279 (2014).

35. D. Aeschimann, K. Lauber, D. M. Moser, J.-P. Theurillat, *Flora alpina: Atlas des 4.500 plantes vasculaires des Alpes Photographies en couleurs de Konrad Lauber et croquis d'André Michel* (Belin, 2004).

36. H. Qian, R. E. Ricklefs, W. Thuiller, Evolutionary assembly of flowering plants into sky islands. *Nat. Ecol. Evol.* **5**, 640–646 (2021).

37. S. Manafzadeh, Y. M. Staedler, E. Conti, Visions of the past and dreams of the future in the Orient: The Irano-Turanian region from classical botany to evolutionary studies. *Biol. Rev.* **92**, 1365–1388 (2017).

38. W. Ding, D. Silvestro, R. E. Onstein, M. Wu, Z. Zhou, Y. Xing, The stepwise rise of angiosperm-dominated terrestrial ecosystems. *Biol. Rev.* **100**, 2131–2149 (2025).

39. T. D. Herbert, K. T. Lawrence, A. Tzanova, L. C. Peterson, R. Caballero-Gill, C. S. Kelly, Late Miocene global cooling and the rise of modern ecosystems. *Nat. Geosci.* **9**, 843–847 (2016).

40. S. A. Elias, B. Crocker, The Bering Land Bridge: A moisture barrier to the dispersal of steppe-tundra biota? *Quat. Sci. Rev.* **27**, 2473–2483 (2008).

41. A. Graham, The role of land bridges, ancient environments, and migrations in the assembly of the North American flora. *J. Syst. Evol.* **56**, 405–429 (2018).

42. M. Hoffmann, K. von Hagen, E. Hörandl, M. Röser, N. Tkach, Sources of the Arctic Flora: Origins of arctic species in *Ranunculus* and related genera. *Int. J. Plant Sci.* **171**, 90–106 (2010).

43. J. Zhang, X.-Q. Li, H.-W. Peng, L. Hai, A. S. Erst, F. Jabbour, R. del C. Ortiz, F.-C. Xia, P. S. Soltis, D. E. Soltis, W. Wang, Evolutionary history of the Arctic flora. *Nat. Commun.* **14**, 4021 (2023).

44. S. Li, R. A. Spicer, T. Su, Z. Zhou, C. Deng, An updated chronostratigraphic framework for the Cenozoic sediments of southeast margin of the Tibetan Plateau: Implications for regional tectonics. *Global Planet. Change* **236**, 104436 (2024).

45. F. Wu, X. Fang, Y. Yang, G. Dupont-Nivet, J. Nie, F. Fluteau, T. Zhang, W. Han, Reorganization of Asian climate in relation to Tibetan Plateau uplift. *Nat. Rev. Earth Environ.* **3**, 684–700 (2022).

46. A. Farnsworth, D. J. Lunt, S. A. Robinson, P. J. Valdes, W. H. G. Roberts, P. D. Clift, P. Markwick, T. Su, N. Wrobel, F. Bragg, S.-J. Kelland, R. D. Pancost, Past East Asian monsoon evolution controlled by paleogeography, not CO_2. *Sci. Adv.* **5**, eaax1697 (2019).

47. F. Schlunegger, E. Kissling, Slab rollback orogeny in the Alps and evolution of the Swiss Molasse basin. *Nat. Commun.* **6**, 8605 (2015).

48. M. Steinthorsdottir, H. K. Coxall, A. M. de Boer, M. Huber, N. Barbolini, C. D. Bradshaw, N. J. Burls, S. J. Feakins, E. Gasson, J. Henderiks, A. E. Holbourn, S. Kiel, M. J. Kohn, G. Knorr, W. M. Kürschner, C. H. Lear, D. Liebrand, D. J. Lunt, T. Mörs, P. N. Pearson, M. J. Pound, H. Stoll, C. A. E. Strömberg, The miocene: The future of the past. *Paleoceanogr. Paleoclimatology* **36**, e2020PA004037 (2021).

49. J. W. Kadereit, The role of *in situ* species diversification for the evolution of high vascular plant species diversity in the European Alps–A review and interpretation of phylogenetic studies of the endemic flora of the Alps. *Perspect. Plant Ecol. Evol. Syst.* **26**, 28–38 (2017).

50. F. Mouthereau, O. Lacombe, J. Vergés, Building the Zagros collisional orogen: Timing, strain distribution and the dynamics of Arabia/Eurasia plate convergence. *Tectonophysics* **532-535**, 27–60 (2012).

51. M. Bullen, D. Burbank, J. Garver, Building the Northern Tien Shan: Integrated thermal, structural, and topographic constraints. *J. Geol.* **111**, 149–165 (2003).

52. Y. Jiang, H. Lu, R. Yang, L. Pang, R. Jiao, Y. Wang, J. Pang, Y. Li, Two-stage exhumation, uplift, and basinward propagation of the Tian Shan during the late Cenozoic. *Earth Sci. Rev.* **256**, 104868 (2024).

53. Y. Wang, J. Zhang, X. Huang, Z. Wang, Cenozoic exhumation of the Tianshan as constrained by regional low-temperature thermochronology. *Earth Sci. Rev.* **237**, 104325 (2023).

54. J. Chang, S. Glorie, N. Qiu, K. Min, Y. Xiao, W. Xu, Late Miocene (10.0~6.0 Ma) rapid exhumation of the Chinese South Tianshan: Implications for the timing of aridification in the Tarim Basin. *Geophys. Res. Lett.* **48**, e2020GL090623 (2021).

55. J. Li, W. Liang, Y. Wang, S. Song, Q. Shen, S. Yuan, Pulsed uplift of the South Tianshan since the Late Miocene indicated by the linear inversion on river longitudinal profiles. *Geomorphology* **466**, 109448 (2024).

56. S. D. Willett, Late neogene erosion of the Alps: A climate driver? *Annu. Rev. Earth Planet. Sci.* **38**, 411–437 (2010).

57. F. Herman, D. Seward, P. G. Valla, A. Carter, B. Kohn, S. D. Willett, T. A. Ehlers, Worldwide acceleration of mountain erosion under a cooling climate. *Nature* **504**, 423–426 (2013).

58. B. J. Yanites, T. A. Ehlers, Global climate and tectonic controls on the denudation of glaciated mountains. *Earth Planet. Sci. Lett.* **325-326**, 63–75 (2012).

59. V. K. Pedersen, D. L. Egholm, Glaciations in response to climate variations preconditioned by evolving topography. *Nature* **493**, 206–210 (2013).

60. J. V. Matthews, L. E. Ovenden, Late tertiary plant macrofossils from localities in Arctic/Subarctic North America: A review of the data. *Arctic* **43**, 364–392 (1990).

61. C. Pouchon, A. Fernández, J. M. Nassar, F. Boyer, S. Aubert, S. Lavergne, J. Mavárez, Phylogenomic analysis of the explosive adaptive radiation of the *Espeletia* Complex (Asteraceae) in the Tropical Andes. *Syst. Biol.* **67**, 1041–1060 (2018).

62. S. G. Flantua, A. O'Dea, R. E. Onstein, C. Giraldo, H. Hooghiemstra, The flickering connectivity system of the north Andean páramos. *J. Biogeogr.* **46**, 1808–1825 (2019).

63. H. H. Birks, The Late-Quaternary history of arctic and alpine plants. *Plant Ecol. Divers.* **1**, 135–146 (2008).

64. W.-N. Ding, R. H. Ree, M. May, P. Brun, O. Hagen, D. N. Karger, A. Skeels, L. Pellissier, Y.-W. Xing, N. Zimmermann, The asynchronous rise of Northern Hemisphere alpine floras

reveals general responses of biotic assembly to orogeny and climate change [Dataset]. Dryad (2025); https://doi.org/10.5061/dryad.8931zcs30.

65. J. Liu, R. I. Milne, G.-F. Zhu, R. A. Spicer, M. C. Wambulwa, Z.-Y. Wu, D. E. Boufford, Y.-H. Luo, J. Provan, T.-S. Yi, J. Cai, H. Wang, L.-M. Gao, D.-Z. Li, Name and scale matter: Clarifying the geography of Tibetan Plateau and adjacent mountain regions. *Global Planet. Change* **215**, 103893 (2022).

66. H. Bruelheide, J. Dengler, B. Jiménez-Alfaro, O. Purschke, S. M. Hennekens, M. Chytrá, V. D. Pillar, F. Jansen, J. Kattge, B. Sandel, I. Aubin, I. Biurrun, R. Field, S. Haider, U. Jandt, J. Lenoir, R. K. Peet, G. Peyre, F. M. Sabatini, M. Schmidt, F. Schrodt, M. Winter, S. Aćić, E. Agrillo, M. Alvarez, D. Ambarlı, P. Angelini, I. Apostolova, M. A. S. Arfin Khan, E. Arnst, F. Attorre, C. Baraloto, M. Beckmann, C. Berg, Y. Bergeron, E. Bergmeier, A. D. Bjorkman, V. Bondareva, P. Borchardt, Z. Botta-Dukát, B. Boyle, A. Breen, H. Brisse, C. Byun, M. R. Cabido, L. Casella, L. Cayuela, T. Černá, V. Chepinoga, J. Csiky, M. Curran, R. Ćušterevska, Z. Dajić Stevanović, E. De Bie, P. de Ruffray, M. De Sanctis, P. Dimopoulos, S. Dressler, R. Ejrnæs, M. A. E.-R. M. El-Sheikh, B. Enquist, J. Ewald, J. Fagúndez, M. Finckh, X. Font, E. Forey, G. Fotiadis, I. García-Mijangos, A. L. de Gasper, V. Golub, A. G. Gutierrez, M. Z. Hatim, T. He, P. Higuchi, D. Holubová, N. Hölzel, J. Homeier, A. Indreica, D. Işık Gürsoy, S. Jansen, J. Janssen, B. Jedrzejek, M. Jiroušek, N. Jürgens, Z. Kacki, A. Kavgacı, E. Kearsley, M. Kessler, I. Knollová, V. Kolomiychuk, A. Korolyuk, M. Kozhevnikova, Ł. Kozub, D. Krstonošić, H. Kühl, I. Kühn, A. Kuzemko, F. Küzmič, F. Landucci, M. T. Lee, A. Levesley, C.-F. Li, H. Liu, G. Lopez-Gonzalez, T. Lysenko, A. Macanović, P. Mahdavi, P. Manning, C. Marcenò, V. Martynenko, M. Mencuccini, V. Minden, J. E. Moeslund, M. Moretti, J. V. Müller, J. Munzinger, U. Niinemets, M. Nobis, J. Noroozi, A. Nowak, V. Onyshchenko, G. E. Overbeck, W. A. Ozinga, A. Pauchard, H. Pedashenko, J. Peñuelas, A. Pérez-Haase, T. Peterka, P. Petřík, O. L. Phillips, V. Prokhorov, V. Rašomavičius, R. Revermann, J. Rodwell, E. Ruprecht, S. Rūsiņa, C. Samimi, J. H. Schaminée, U. Schmiedel, J. Šibík, U. Šilc, V. Škvorc, A. Smyth, T. Sop, D. Sopotlieva, B. Sparrow, Z. Stančić, J.-C. Svenning, G. Swacha, Z. Tang, I. Tsiripidis, P. D. Turtureanu, E. Uğurlu, D. Uogintas, M. Valachovič, K. A. Vanselow, Y. Vashenyak, K. Vassilev, E. Vélez-Martin, R. Venanzoni, A. C. Vibrans, C. Violle, R. Virtanen, H. von Wehrden, V. Wagner, D. A. Walker, D. Wana, E. Weiher, K.

Wesche, T. Whitfeld, W. Willner, S. Wiser, T. Wohlgemuth, S. Yamalov, G. Zizka, A. Zverev, sPlot - A new tool for global vegetation analyses. *J. Veg. Sci.* **30**, 161–186 (2019).

67. Y. Chauvier, O. Hagen, C. Albouy, P. Descombes, F. Fopp, M. Nobis, P. Brun, L. Lyu, L. Pellissier, K. Csilléry, Gbif.Range: An R Package to generate species range maps based on ecoregions and a user-friendly GBIF wrapper. EnviDat (2022); www.doi.org/10.16904/envidat.352.

68. A. Zizka, D. Silvestro, T. Andermann, J. Azevedo, C. Duarte Ritter, D. Edler, H. Farooq, A. Herdean, M. Ariza, R. Scharn, S. Svantesson, N. Wengström, V. Zizka, A. Antonelli, CoordinateCleaner: Standardized cleaning of occurrence records from biological collection databases. *Methods Ecol. Evol.* **10**, 744–751 (2019).

69. R. Testolin, F. Attorre, B. Jiménez-Alfaro, Global distribution and bioclimatic characterization of alpine biomes. *Ecography* **43**, 779–788 (2020).

70. C. Körner, J. Paulsen, E. M. Spehn, A definition of mountains and their bioclimatic belts for global comparisons of biodiversity data. *Alp. Bot.* **121**, 73–78 (2011).

71. D. N. Karger, M. Kessler, O. Conrad, P. Weigelt, H. Kreft, C. König, N. E. Zimmermann, Why tree lines are lower on islands–Climatic and biogeographic effects hold the answer. *Glob. Ecol. Biogeogr.* **28**, 839–850 (2019).

72. S. Höhna, M. J. Landis, T. A. Heath, B. Boussau, N. Lartillot, B. R. Moore, J. P. Huelsenbeck, F. Ronquist, RevBayes: Bayesian phylogenetic inference using graphical models and an interactive model-specification language. *Syst. Biol.* **65**, 726–736 (2016).

73. W. A. Freyman, S. Höhna, Cladogenetic and anagenetic models of chromosome number evolution: A Bayesian model averaging approach. *Syst. Biol.* **67**, 195–215 (2018).

74. W. A. Freyman, S. Höhna, Stochastic character mapping of state-dependent diversification reveals the tempo of evolutionary decline in self-compatible onagraceae lineages. *Syst. Biol.* **68**, 505–519 (2019).

75. M. R. May, X. Meyer, TensorPhylo RevBayes plugin. Bitbucket (2022); https://bitbucket.org/mrmay/tensorphylo/src/master.

76. J.-C. Foltête, G. Vuidel, P. Savary, C. Clauzel, Y. Sahraoui, X. Girardet, M. Bourgeois, Graphab: An application for modeling and managing ecological habitat networks. *Softw. Impact.* **8**, 100065 (2021).

77. J.-C. Foltête, C. Clauzel, G. Vuidel, A software tool dedicated to the modelling of landscape networks. *Environ. Model. Software* **38**, 316–327 (2012).

78. S. Saura, L. Pascual-Hortal, A new habitat availability index to integrate connectivity in landscape conservation planning: Comparison with existing indices and application to a case study. *Landsc. Urban Plan.* **83**, 91–103 (2007).

79. L. Liu, L. Liu, J. P. Morgan, Y.-G. Xu, L. Chen, New constraints on Cenozoic subduction between India and Tibet. *Nat. Commun.* **14**, 1963 (2023).

80. M. H. Darin, P. J. Umhoefer, S. N. Thomson, Rapid Late Eocene exhumation of the Sivas Basin (Central Anatolia) driven by initial Arabia-Eurasia Collision. *Tectonics* **37**, 3805–3833 (2018).

81. M. Coiro, J. A. Doyle, J. Hilton, How deep is the conflict between molecular and fossil evidence on the age of angiosperms? *New Phytol.* **223**, 83–99 (2019).

82. G. J. Jordan, M. K. Macphail, A Middle-Late Eocene inflorescence of Caryophyllaceae from Tasmania, Australia. *Am. J. Bot.* **90**, 761–768 (2003).

83. H.-T. Li, T.-S. Yi, L.-M. Gao, P.-F. Ma, T. Zhang, J.-B. Yang, M. A. Gitzendanner, P. W. Fritsch, J. Cai, Y. Luo, H. Wang, M. van der Bank, S.-D. Zhang, Q.-F. Wang, J. Wang, Z.-R. Zhang, C.-N. Fu, J. Yang, P. M. Hollingsworth, M. W. Chase, D. E. Soltis, P. S. Soltis, D.-Z. Li, Origin of angiosperms and the puzzle of the Jurassic gap. *Nat. Plants* **5**, 461–470 (2019).

84. A. B. Doweld, Silene novorossica, a new name for fossil *Silene mirabilis* (Caryophyllaceae). *Ann. Bot. Fenn.* **53**, 113–114 (2016).

85. D. H. Mai, Die oberoligozänen Floren am Nordrand der sächsischen Lausitz. *Palaeontogr. Abt. B* **1–6**, 1–124 (1997).

86. J. Van der Burgh, Miocene floras in the lower Rhenish Basin and their ecological interpretation. *Rev. Palaeobot. Palynol.* **52**, 299–366 (1987).

87. Y. Wu, H.-L. You, X.-Q. Li, Dinosaur-associated Poaceae epidermis and phytoliths from the Early Cretaceous of China. *Natl. Sci. Rev.* **5**, 721–727 (2018).

88. S. Y. Smith, M. E. Collinson, D. A. Simpson, P. J. Rudall, F. Marone, M. Stampanoni, Elucidating the affinities and habitat of ancient, widespread Cyperaceae: *Volkeria messelensis* gen. et sp. nov., a fossil mapanioid sedge from the Eocene of Europe. *Am. J. Bot.* **96**, 1506–1518 (2009).

89. J. G. Conran, J. M. Bannister, D. E. Lee, R. J. Carpenter, E. M. Kennedy, T. Reichgelt, R. E. Fordyce, An update of monocot macrofossil data from New Zealand and Australia. *Bot. J. Linn. Soc.* **178**, 394–420 (2015).

90. D. Silvestro, C. D. Bacon, W. Ding, Q. Zhang, P. C. J. Donoghue, A. Antonelli, Y. Xing, Fossil data support a pre-Cretaceous origin of flowering plants. *Nat. Ecol. Evol.* **5**, 449–457 (2021).

91. H. D. Mai, Die untermiozän Floren aus der Spremberger Folge und dem 2. Flözhorizont in der Lausitz. Teil I. Farnpflanzen. Koniferen und Monokotyledonen. *Palaeontographica Abteilung B* **250**, 1–76 (1999).

92. W. Wang, L. Lin, X.-G. Xiang, R. del C. Ortiz, Y. Liu, K.-L. Xiang, S.-X. Yu, Y.-W. Xing, Z.-D. Chen, The rise of angiosperm-dominated herbaceous floras: Insights from Ranunculaceae. *Sci. Rep.* **6**, 27259 (2016).

93. L. Zhang, X. Zhu, Y. Zhao, J. Guo, T. Zhang, W. Huang, J. Huang, Y. Hu, C.-H. Huang, H. Ma, Phylotranscriptomics resolves the phylogeny of pooideae and uncovers factors for their adaptive evolution. *Mol. Biol. Evol.* **39**, msac026 (2022).

94. D. W. Edelman, “The Eocene Germer Basin Flora of South-Central Idaho,” thesis, University of Idaho (1975).

95. H. D. Mai, Die mittelmiozänen und obermiozänen Floren aus der Meuroer und Raunoer Folge in der Lausitz. Teil II: Dicotyledonen. *Palaeontographica Abteilung B* **257**, 35–174 (2001).

96. J. A. Wolfe, H. E. Schorn, “Taxonomic revision of the Spermatopsida of the Oligocene Creede flora, southern Colorado” (Bulletin Series 1923, US Geological Survey, 1990).

97. M. Töpel, A. Antonelli, C. Yesson, B. Eriksen, Past climate change and plant evolution in Western North America: A case study in Rosaceae. *PLOS ONE* **7**, e50358 (2012).

98. C. Dobeš, J. Paule, A comprehensive chloroplast DNA-based phylogeny of the genus *Potentilla* (Rosaceae): Implications for its geographic origin, phylogeography and generic circumscription. *Mol. Phylogen. Evol.* **56**, 156–175 (2010).

99. M. L. DeVore, S. M. Moore, K. B. Pigg, W. C. Wehr, Fossil Neviusia Leaves (Rosaceae: Kerrieae) from the Lower-Middle Eocene of Southern British Columbia. *Rhodora* **106**, 197–209 (2004).

100. M. Lavin, P. S. Herendeen, M. F. Wojciechowski, Evolutionary rates analysis of leguminosae implicates a rapid diversification of lineages during the tertiary. *Syst. Biol.* **54**, 575–594 (2005).

101. N. Azani, A. Bruneau, M. F. Wojciechowski, S. Zarre, Miocene climate change as a driving force for multiple origins of annual species in *Astragalus* (Fabaceae, Papilionoideae). *Mol. Phylogen. Evol.* **137**, 210–221 (2019).

102. G. Mansion, G. Parolly, A. A. Crowl, E. Mavrodiev, N. Cellinese, M. Oganesian, K. Fraunhofer, G. Kamari, D. Phitos, R. Haberle, G. Akaydin, N. Ikinci, T. Raus, T. Borsch, How to handle speciose clades? Mass taxon-sampling as a strategy towards illuminating the natural history of *Campanula* (Campanuloideae). *PLOS ONE* **7**, e50076 (2012).

103. K. E. Jones, N. Korotkova, J. Petersen, T. Henning, T. Borsch, N. Kilian, Dynamic diversification history with rate upshifts in Holarctic bell-flowers (*Campanula* and allies). *Cladistics* **33**, 637–666 (2017).

104. T. J. Givnish, A. Zuluaga, I. Marques, V. K. Y. Lam, M. S. Gomez, W. J. D. Iles, M. Ames, D. Spalink, J. R. Moeller, B. G. Briggs, S. P. Lyon, D. W. Stevenson, W. Zomlefer, S. W. Graham, Phylogenomics and historical biogeography of the monocot order Liliales: Out of Australia and through Antarctica. *Cladistics* **32**, 581–605 (2016).

105. J. S. Kim, J.-H. Kim, Updated molecular phylogenetic analysis, dating and biogeographical history of the lily family (Liliaceae: Liliales). *Bot. J. Linn. Soc.* **187**, 579–593 (2018).

106. S. Manchester, W. Judd, B. Handley, Foliage and fruits of early poplars (Salicaceae: *Populus*) from the Eocene of Utah, Colorado, and Wyoming. *Int. J. Plant Sci.* **167**, 897–908 (2006).

107. M. E. Collinson, The early fossil history of Salicaceae: A brief review. *Proc. R. Soc. Edinb., Sect. B, Biol.* **98**, 155–167 (1992).

108. J. P. Rose, C.-L. Xiang, K. J. Sytsma, B. T. Drew, A timeframe for mint evolution: Towards a better understanding of trait evolution and historical biogeography in Lamiaceae. *Bot. J. Linn. Soc.* **200**, 15–38 (2022).

109. J. P. Rose, J. Wiese, N. Pauley, T. Dirmenci, F. Celep, C.-L. Xiang, B. T. Drew, East Asian-North American disjunctions and phylogenetic relationships within subtribe Nepetinae (Lamiaceae). *Mol. Phylogen. Evol.* **187**, 107873 (2023).

110. E. M. Joyce, D. M. Crayn, V. K. Y. Lam, W. K. Gerelle, S. W. Graham, L. Nauheimer, Evolution of *Geosiris* (Iridaceae): Historical biogeography and plastid-genome evolution in a genus of non-photosynthetic tropical rainforest herbs disjunct across the Indian Ocean. *Aust. Syst. Bot.* **31**, 504–522 (2018).

111. T.-S. Han, Q.-J. Zheng, R. E. Onstein, B. M. Rojas-Andrés, F. Hauenschild, A. N. Muellner-Riehl, Y.-W. Xing, Polyploidy promotes species diversification of *Allium* through ecological shifts. *New Phytol.* **225**, 571–583 (2020).

112. S. Martín-Bravo, P. Jiménez-Mejías, T. Villaverde, M. Escudero, M. Hahn, D. Spalink, E. H. Roalson, A. L. Hipp, the Global Carex Group, C. Benítez-Benítez, L. P. Bruederle, E. Fitzek, B. A. Ford, K. A. Ford, M. Garner, S. Gebauer, M. H. Hoffmann, X.-F. Jin, I. Larridon, E. Lévéillé-Bourret, Y.-F. Lu, M. Luceño, E. Maguilla, J. I. Márquez-Corro, M. Míguez, R. Naczi, A. A. Reznicek, J. R. Starr, A tale of worldwide success: Behind the scenes of *Carex* (Cyperaceae) biogeography and diversification. *J. Syst. Evol.* **57**, 695–718 (2019).

113. A. Favre, I. Michalak, C.-H. Chen, J.-C. Wang, J. S. Pringle, S. Matuszak, H. Sun, Y.-M. Yuan, L. Struwe, A. N. Muellner-Riehl, Out-of-Tibet: The spatio-temporal evolution of *Gentiana* (Gentianaceae). *J. Biogeogr.* **43**, 1967–1978 (2016).

114. I. Juramurodov, D. Makhmudjanov, P.-L. Liu, Z. Yusupov, E. Nikitina, T. Deng, K. Tojibaev, H. Sun, Phylogenetic relationships and biogeography in *Hedysarum* (Hedysareae, Fabaceae) with a focus on Central Asian taxa. *Taxon* **72**, 1262–1284 (2023).

115. R. A. Folk, R. L. Stubbs, M. E. Mort, N. Cellinese, J. M. Allen, P. S. Soltis, D. E. Soltis, R. P. Guralnick, Rates of niche and phenotype evolution lag behind diversification in a temperate radiation. *Proc. Natl. Acad. Sci. U.S.A.* **116**, 10874–10882 (2019).

116. X. Zhang, J. B. Landis, Y. Sun, H. Zhang, N. Lin, T. Kuang, X. Huang, T. Deng, H. Wang, H. Sun, Macroevolutionary pattern of *Saussurea* (Asteraceae) provides insights into the drivers of radiating diversification. *Proc. R. Soc. Lond. Ser. B. Biol. Sci.* **288**, 20211575 (2021).

117. M. Doostmohammadi, F. Bordbar, D. C. Albach, M. Mirtadzadini, Phylogeny and historical biogeography of *Veronica* subgenus pentasepalae (Plantaginaceae): Evidence for its origin and subsequent dispersal. *Biology* **11**, 639 (2022).
